# A curved host and second guest cooperatively inhibit the dynamic motion of corannulene

**DOI:** 10.1038/s41467-021-24344-w

**Published:** 2021-07-02

**Authors:** Yang Yang, Tanya K. Ronson, Zifei Lu, Jieyu Zheng, Nicolas Vanthuyne, Alexandre Martinez, Jonathan R. Nitschke

**Affiliations:** 1 Department of Chemistry, University of Cambridge, Cambridge, UK; 2 School of Chemistry and Materials Science, Jiangsu Normal University, Xuzhou, China; 3 Aix Marseille Univ, CNRS, Centrale Marseille, iSm2, Marseille, France

**Keywords:** Inorganic chemistry, Molecular capsules, Self-assembly

## Abstract

Biomolecular systems show how host–guest binding can induce changes in molecular behavior, which in turn impact the functions of the system. Here we report an artificial host–guest system where dynamic adaptation during guest binding alters both host conformation and guest dynamics. The self-assembled cage host employed here possesses concave walls and a chirotopic cavity. Complementarity between the curved surfaces of fullerenes and the inner surface of the host cavity leads the host to reconfigure stereochemically in order to bind these guests optimally. The curved molecule corannulene undergoes rapid bowl-to-bowl inversion at room temperature. Its inversion barrier is increased upon binding, however, and increased further upon formation of a ternary complex, where corannulene and a cycloalkane are both bound together. The chiral nature of the host also leads to clear differences in the NMR spectra of ternary complexes involving corannulene and one or the other enantiomer of a chiral guest, which enables the determination of enantiomeric excess by NMR.

## Abstract (web summary)

The preparation of artificial host–guest systems that display dynamic adaptation during guest binding is challenging. Here the authors report a chiral self-assembled tetrahedral cage featuring curved walls that reconfigures stereochemically to fit fullerene guests, regulates corannulene inversion, and enables the determination of co-guest enantiomeric excess by NMR spectroscopy.

## Introduction

Recognition and binding processes involving biomolecules, such as enzyme–substrate, antibody–antigen, and protein–protein, are integral to living systems. In many cases, the conformations and shapes of biomacromolecules can change to fit the target substrate, in a process known as induced fit^1^, or an auxiliary substance may bind to a host–guest complex to form a ternary complex, thus regulating or modulating the initial interaction^2^. Artificial molecular systems that mimic the sophisticated processes of induced fit and property regulation via ternary complex formation are challenging to design, but important in producing complex and functional systems of molecules^3–5^, and in understanding the molecular basis of binding in relevant biological processes.

Curved aromatic molecules are well-suited to investigations into complex host–guest phenomena, as their interactions principally involve dispersion forces. These include fullerenes^6^, which have promising applications as light-harvesting materials^7^, and corannulene, a bowl-shaped hydrocarbon that can be considered a sub-unit of a fullerene. Corannulene undergoes rapid bowl inversion at room temperature, with an energy barrier calculated to be 11.5 kcal mol^−1^ ^8,9^. Stabilization of the planar transition state to corannulene inversion has been achieved by threading this molecule through a cationic macrocycle^10^, and through compression within an anthracene-walled host^11^. Manipulation of corannulene to slow its inversion rate, in contrast, is a harder goal, having only been achieved through chemical modifications^12^.

Metal-organic cages provide confined inner phases that are usefully distinct from the outside environment^13^. These cavities can be used for the stabilization of labile species^14,15^, separations^16–18^, and catalysis^19–24^. Principles have been developed to enable the selective binding of specific guests, or classes of guests, from among many chemically similar prospective guests in solution^25–30^. These principles guide the design of artificial hosts to maximize host–guest interactions, with greater shape complementarity often leading to additional selectivity and affinity^31,32^.

In this work, we employ these ideas to prepare a host with curved walls, designed to interact with the curved π-conjugated surfaces of fullerenes and corannulene. Fullerenes induce a configurational change in the host, highlighting the impact of the guest on the host. The dynamic inversion of corannulene is restricted inside the host, demonstrating host impact on the guest. Further, co-guests form ternary complexes, resulting in further modulation of the behavior of both guests inside the cage. The corannulene guest also reports on the stereochemistry of a chiral co-guest.

## Results

### Synthesis and characterization of **1**

**Fig. 1:**
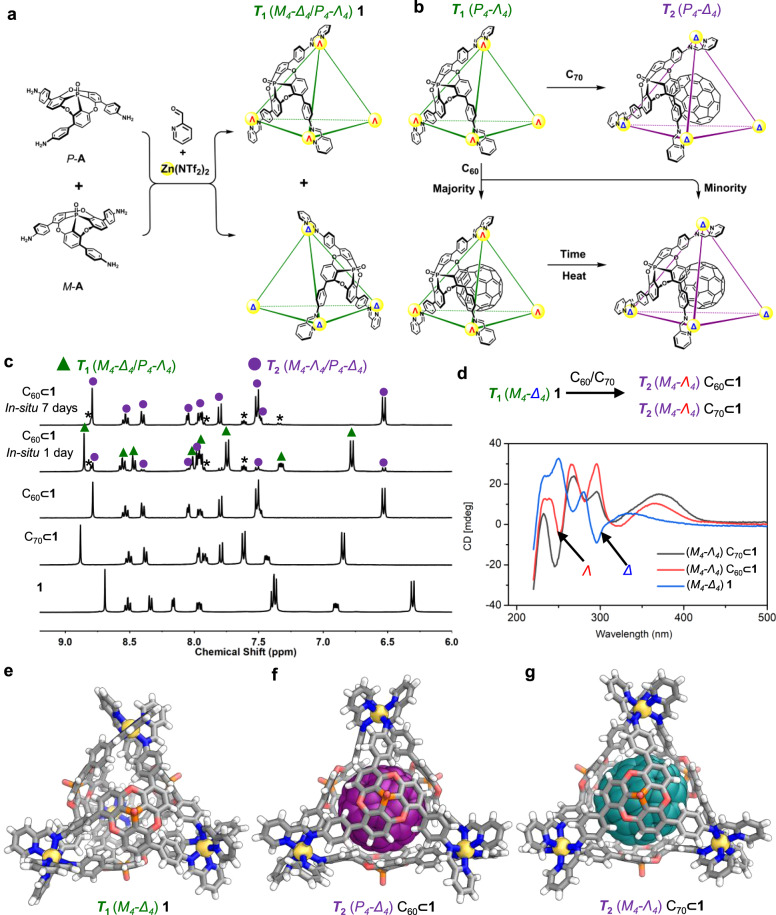
Synthesis of cage 1 and induced fit of C_60_ and C_70_ guests. **a** The self-sorting preparation of cage **1** from chiral subcomponent** A** as a single pair of enantiomers. **b** The conversion of cage diastereomer *T*_1_ into *T*_2_ upon fullerene binding. **c** ^1^H NMR spectra of **1** and its C_70_ and C_60_ adducts, showing the evolution of the C_60_ adduct from the *T*_1_ configuration (green triangles) into the *T*_2_ (violet circles) over 7 days; residual 2-formylpyridine signals are labeled with asterisks. **d** CD spectra of 0.03 mM acetonitrile solutions of the host and host–guest complexes prepared with enantiopure subcomponent *M*-**A** (spectra for assemblies with subcomponent *P*-**A** are shown in Supplementary Fig. 84). **g** X-ray crystal structures showing **1** in its preferred *T*_1_ configuration (**e**) in the absence of a fullerene guest; and the *T*_2_ configuration it adopts when binding (**f**) C_60_ or (**g**) C_70_, respectively.

Subcomponent **A** was designed around a phosphangulene core (Fig. 1a), which was selected due to its chirality, large concave surface, and bowl-shaped conformation that does not invert at room temperature^33–35^. Cage **1**, with a spherical interior cavity, was obtained via the subcomponent self-assembly of **A** with 2-formylpyridine and zinc(II) bis(trifluoromethylsulfonyl)imide (Zn(NTf_2_)_2_) in acetonitrile.

The self-assembly of cage **1** from racemic **A** gives rise to a large set of stereochemical possibilities, as each metal center may adopt *Δ* or *Λ* handedness, each ligand may possess *M* or *P* helicity. The observation of only one set of ligand proton signals in the ^1^H NMR spectrum of **1** allows us to infer that self-assembly occurred stereoselectively in two ways. First, the two ligand enantiomers narcissistically self-sorted (Fig. 1a, c), such that each cage incorporated either only *M* ligands or only *P* ligands. Second, the *M* or *P* chirality of the ligands within a cage dictated the *Δ* or *Λ* configuration of all of its metal centers. Thus, out of the two possible diastereomeric pairs of enantiomers of cage **1**, labeled *T*_1_ (*M*_*4*_−*Δ*_*4*_, *P*_*4*_−*Λ*_*4*_) and *T*_2_ (*M*_*4*_−*Λ*_*4*_, *P*_*4*_−*Δ*_*4*_)^36^, only one was observed. The X-ray crystal structure of **1** showed that **1** adopted exclusively the *T*_1_ configuration (Fig. 1e).

### Impact of guests on the host

Cage **1** was anticipated to bind C_60_ and C_70_ through the curved π surfaces of its **A** residues^34^, in a similar fashion to Sygula’s “buckycatcher”^37^. When **1** was prepared in the presence of C_70_ or C_60_, new ^1^H NMR signals were observed, corresponding to the host–guest complex. Only one such set of signals was observed in the case of C_70_, whereas two sets of signals were observed for C_60_, suggesting the presence of two diastereomeric host configurations (Fig. 1c). After heating the reaction mixture at 70 °C for one week, the initial major set of signals disappeared, and the minor set increased to become the only product. X-ray crystallography revealed that **1** bound both C_60_ and C_70_ in the *T*_2_ configuration (Fig. 1f, g).

Based upon X-ray data, the VOIDOO program^38^ was used to calculate the volume of **1** in the absence of a fullerene guest (Fig. 1e), and as its C_60_ (Fig. 1f) and C_70_ (Fig. 1g) adducts, providing volumes of 490 Å^3^, 718 Å^3^ and 925 Å^3^, respectively (Supplementary Fig. 85). The cavity size of **1** in the *T*_1_ configuration is much smaller than that of the *T*_2_ configuration adopted for the fullerene adducts. Larger C_70_ appears only to fit in the *T*_2_ diastereomer, whereas smaller C_60_ can be accommodated in both configurations. We infer that the *T*_1_ diastereomer of C_60_⊂**1** is kinetically favored, but that the *T*_2_ adduct is favored thermodynamically, based upon the initial formation of the former, and its transformation into the latter upon heating (Fig. 1c). Intriguingly, within the chirotopic^39^ cavity of **1**, the local symmetry of encapsulated C_70_ is broken from *D*_5h_ to *D*_5_, resulting in the splitting of its two highest-intensity ^13^C NMR peaks into two sets of signals (Supplementary Fig. 26)^40^.

Racemic triamine **A** was resolved into two enantiomers by chiral HPLC (Supplementary Section 8), enabling the construction of enantiopure (*P*_*4*_−*Λ*_*4*_)**1** and (*M*_*4*_−*Δ*_*4*_)**1**. The transformation of **1** from its *T*_1_ configuration to *T*_2_ following guest uptake was confirmed by CD studies of enantiopure **1** and its corresponding fullerene host–guest complexes (Fig. 1d and Supplementary Fig. 84). The CD signals around 240–320 nm, corresponding to π–π* transitions, are correlated with the handedness of the metal vertices^41,42^. The signals of C_60_⊂**1** and C_70_⊂**1** at this region are similar in magnitude, but with opposite signs to those of fullerene-free **1** for complexes prepared from a given enantiomer of **A** (Fig. 1d). The differences in size and chirality between the empty host and host–guest complexes illustrate that the cages can reconfigure stereochemically to adapt to large guests with curved surfaces.

### Impact of the host on guests

**Fig. 2:**
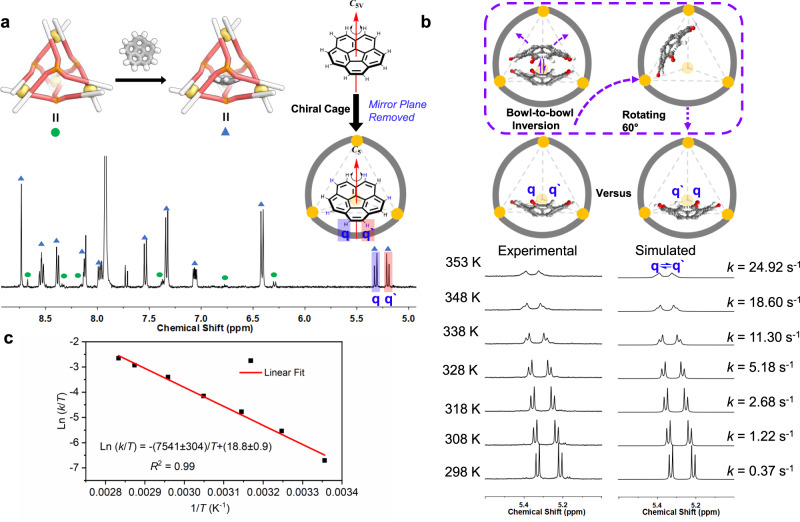
Encapsulation of corannulene and its dynamic inversion. **a** ^1^H NMR spectrum (CD_3_CN, 400 MHz, 298 K) of **1** mixed with excess corannulene. Green circles correspond to **1** and light-blue triangles to corannulene⊂**1**. **b** Schematic illustration of the exchange of adjacent protons H_q_ and H_q′_ during corannulene inversion, signals of encapsulated corannulene in variable temperature ^1^H NMR spectra (CD_3_CN, 500 MHz) of cage **1** with corannulene guests, and the corresponding simulated spectra obtained by line-shape analysis. After inversion, H_q_ (red) has exchanged with H_q′_ (white) relative to the host. The rolling of the corannulene without inversion within the four ligand walls will not exchange protons H_q_ and H_q′_. **c** Eyring plot for the inversion of corannulene inside of **1**.

Its concave ligands also enable **1** to encapsulate the bowl-shaped molecule corannulene selectively from among a series of polyaromatic hydrocarbons (Supplementary Section 2.5). Corannulene also experienced the chirotopic environment inside the cage, with its single-peak ^1^H NMR spectrum splitting into two coupled signals. This observation is consistent with a local desymmetrization from the *C*_5v_ point symmetry of free corannulene to the *C*_5_ symmetry imposed by a chiral environment (Fig. 2a)^43^. NMR integration indicated that only one corannulene was encapsulated. We infer **1** to remain in its *T*_1_ configuration upon corannulene binding, based upon the NMR similarity to the free cage and crystallographic evidence, as noted below. The binding constant for corannulene within **1** was determined to be *K*_a_ = (1.1 ± 0.1) × 10^3^ M^−1^ (Supplementary Section 3.1).

Cage **1** was also observed to bind pyrene, in a strongly cooperative fashion, such that only free **1** and (pyrene)_2_⊂**1** were observed by ^1^H NMR. Competitive binding experiments indicated a higher affinity of **1** for corannulene over pyrene, which may result from the better fit of the bowl-shaped corannulene to the concave ligands (Supplementary Fig. 42). Planar coronene, which is structurally similar to curved corannulene, was not encapsulated. The slightly larger size may not be a decisive factor for the non-encapsulation of coronene here since **1** can adjust its configuration for large guests such as C_70_. As other cages that encapsulate corannulene were also observed to bind coronene^11,16,44,45^, the binding of corannulene but not coronene within **1** highlights the key role of shape complementarity in its binding preferences.

The chirotopic cavity of **1** enables the study of guest dynamics that would otherwise be concealed by symmetry^46^. The inversion of free corannulene in the solution cannot be studied by NMR, as its hydrogen atoms are all symmetry-equivalent^9^. Within **1**, however, corannulene shows two sets of proton signals, because the adjacent protons H_q_ and H_q′_ (Fig. 2b) are diastereotopic in a chiral space. When corannulene inversion occurs, H_q_ and H_q′_ exchange, as observed by EXSY NMR (Supplementary Fig. 82). VT ^1^H NMR showed that the signals of the encapsulated corannulene broaden with increased temperature while the signals for the host remain sharp, indicating bowl-to-bowl inversion becomes faster at higher temperatures (Fig. 2b). The exchange rate constants *k* were obtained through line-shape analyzes (Supplementary Section 4). Based on an Eyring plot (Fig. 2c), the activation energy *ΔG*^‡^ (298 K) was determined to be 17.9 ± 0.3 kcal mol^−1^, which is notably higher than the experimentally-determined value for a monosubstituted corannulene (10.3 kcal mol^−1^ at 206 K)^8^ and the extrapolated value for free corannulene (11.5 kcal mol^−1^ at 298 K)^9^.

Macrocyclic ExBox^4+^ was found to accelerate the bowl-to-bowl inversion of corannulene bound within it, by stabilizing the planar transition state^10^. We infer that cage **1**, in contrast, acted to stabilize the ground state of curved corannulene through binding to the curved inner surface of the cage. Thus, encapsulation inside **1** raises the barrier to corannulene inversion, provided the transition state is not also stabilized.

### Heterotropic guest interactions inside the cage

Corannulene may be considered an elementary subunit of a fullerene, albeit with reduced curvature and size. Since corannulene only forms a 1:1 host–guest complex with **1**, we infer that space remains to accommodate a second guest. Cycloalkanes (C_n_H_2n_) with five to eight carbons were found to be suitable second guests, forming ternary complexes. The encapsulation of a second guest was signaled by shifts in the ^1^H NMR spectra (Supplementary Section 2.6).

**Fig. 3:**
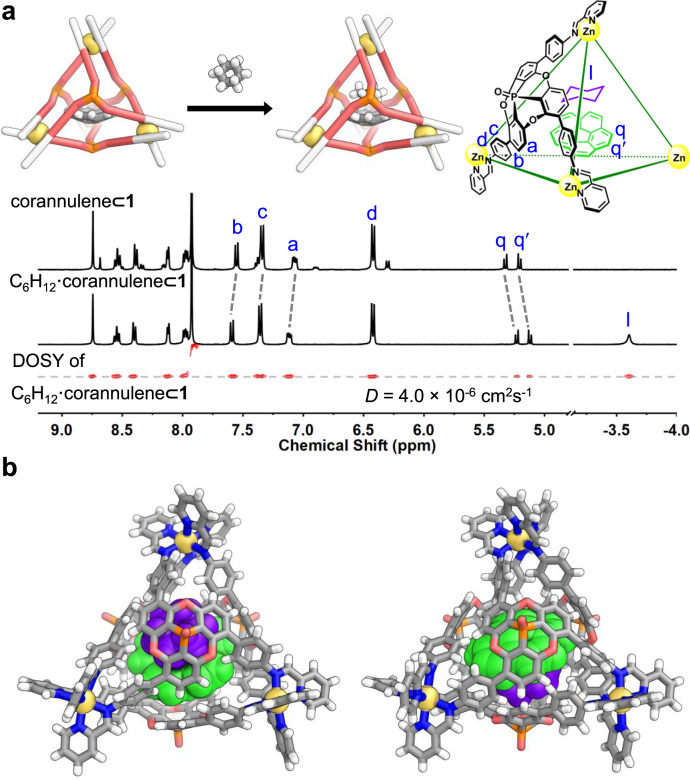
Ternary complex formation. **a** Scheme of the binding of corannulene and cyclohexane within **1**, and comparison of the ^1^H NMR spectra (CD_3_CN, 400 MHz, 298 K) of corannulene⊂**1** and C_6_H_12_•corannulene⊂**1**, together with the ^1^H DOSY spectrum of C_6_H_12_•corannulene⊂**1**. **b** Two views of the cationic part of the crystal structure of C_6_H_12_•corannulene⊂**1**, showing cyclohexane in purple and corannulene in green.

Ternary complexes were observed to form for guests that did not bind in the absence of corannulene, such as cyclohexane. Upon addition of excess cyclohexane to a solution of **1** containing corannulene, a new singlet was observed at −3.58 ppm for encapsulated cyclohexane, upfield shifted by 5.05 ppm compared to the free guest (Fig. 3a). The encapsulated corannulene was also shifted further upfield, and host signals shifted downfield, as compared with corannulene⊂**1**. DOSY NMR indicated the same diffusion coefficient for cage **1** and its corannulene and C_6_H_12_ guests (Fig. 3a). A ^1^H NOESY experiment indicated spatial proximity between the second guest and the encapsulated corannulene (Supplementary Fig. 49). The formation of C_6_H_12_•corannulene⊂**1** was also evidenced by ESI-MS. The binding constants *K*_a1_ (for corannulene) and *K*_a2_ (for cycloalkanes) are shown in Supplementary Table 1.

The X-ray crystal structure of C_6_H_12_•corannulene⊂**1** (Fig. 3b) further verified the formation of the ternary complex, with the host in the smaller *T*_1_ configuration. Although the guests exhibited evidence of thermal motion, the positions and orientations of both guests were clear, with cyclohexane nestled inside the concavity of corannulene, which in turn stacked with the concave cage ligand.

In the presence of a co-encapsulated cycloalkane, corannulene inversion was no longer observed by signal coalescence in the NMR even at 348 K (Supplementary Figs. 77–80). Line shape analysis (Supplementary Fig. 81) indicated an energetic barrier (Δ*G*^‡^) to corannulene inversion at least 3.7 kcal mol^−1^ greater for C_8_H_16_•corannulene⊂**1** than for corannulene⊂**1** at 348 K, cumulative with the 6 kcal mol^−1^ barrier increase due to encapsulation within **1** (Fig. 2b). The second guest within the corannulene bowl takes up space, further inhibiting inversion and reinforcing the effect of the concave ligands of **1** in stabilizing the bowl state of corannulene.

The inversion of cyclohexane from one chair conformation to the other was also suppressed in C_6_H_12_•corannulene⊂**1**. Upon lowering the temperature to 243 K, two separate broad peaks for the equatorial and axial protons of encapsulated cyclohexane were observed, which collapsed into a single peak and sharpened above 253 K (Supplementary Fig. 76). A ring-flip barrier of 9.70 kcal mol^−1^ for cyclohexane was reported at 206.5 K^47^, whereas its *ΔG*^‡^ of inversion inside C_6_H_12_•corannulene⊂**1** was extrapolated to be 10.65 ± 0.03 kcal mol^−1^ at this temperature^47^. Co-encapsulation within a single host thus slows down the dynamic motion of both guests, with each guest having an influence upon the dynamics of the other within the host–guest–guest system.

### Stereochemical communication between guests

Enantiopure *R*-3-methyl-2-butanol (*R*-**MB**) also bound within **1** together with corannulene, requiring this co-guest to bind (Supplementary Section 2.7). The signals of encapsulated corannulene are sensitive to the stereochemistry of the second guest, splitting into two sets due to the formation of the diastereomers *R*-**MB**•corannulene⊂(*M*_*4*_−*Δ*_*4*_)**1** and *R*-**MB**•corannulene⊂(*P*_*4*_−*Λ*_*4*_)**1** (Supplementary Fig. 64). Two well-separated sets of signals of equal intensity were observed for the encapsulated *R*-**MB**, with upfield guest shifts together with a DOSY NMR spectrum providing evidence of encapsulation (Supplementary Fig. 65). No difference in the integrated intensities of the host–guest complexes were observed, indicating that the two enantiomeric complexes corannulene⊂(*M*_*4*_−*Δ*_*4*_)**1** and corannulene⊂(*P*_*4*_−*Λ*_*4*_)**1** do not discriminate when binding the enantiomers of **MB**.

**Fig. 4:**
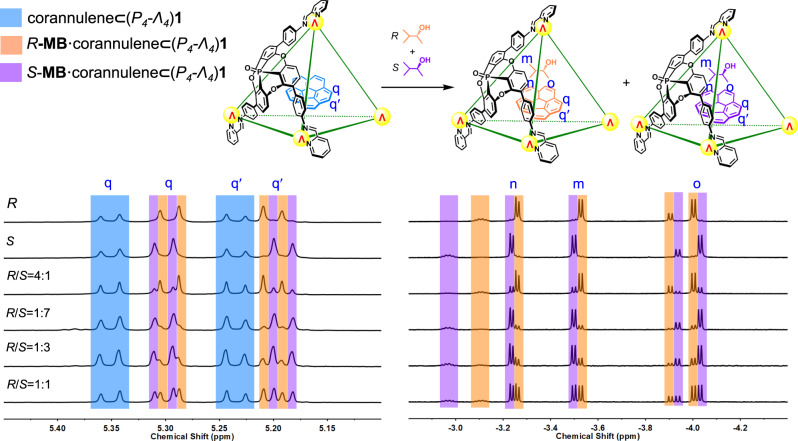
Stereochemical information reported by corannulene and 3-methyl-2-butanol enantiomeric excess (ee) determinations by NMR. Partial ^1^H NMR spectra (CD_3_CN, 500 MHz, 298 K) of solutions of enantiopure cage (*P*_*4*_−*Λ*_*4*_)**1** together with corannulene and different ratios of *R*/*S*-3-methyl-2-butanol (*R*/*S*-**MB**) showing the bound guest signals. The peaks of corannulene⊂(*P*_*4*_−*Λ*_*4*_)**1**, *R*-**MB**•corannulene⊂(*P*_*4*_−*Λ*_*4*_)**1** and *S*-**MB**•corannulene⊂(*P*_*4*_−*Λ*_*4*_)**1** are marked in light blue, orange, and violet, respectively.

Enantiopure cage (*P*_*4*_−*Λ*_*4*_)**1** together with corannulene and racemic **MB** produced similar ^1^H NMR spectra, corroborating non-enantioselective guest binding (Fig. 4). The aromatic shielding effects of corannulene⊂**1** shift the proton signals of the bound guests upfield by more than 5 ppm, into the spectral region below 0 ppm, without interfering signals. The ratio between *R*-**MB** and *S*-**MB** bound inside corannulene⊂**1** is inferred to reflect the ratio between these enantiomers in solution, due to the lack of enantioselectivity in binding. These two factors enable corannulene⊂**1** to be used as an NMR spectroscopic probe for the direct determination of the enantiomeric excess (ee) of chiral **MB**.

To support this hypothesis, **MB** mixtures with different degrees of ee were combined with (*P*_4_−*Λ*_4_)**1** and corannulene (Supplementary Section 5). The integrations of the proton signals of encapsulated **MB** reflected well the ratios between the *R* and *S* enantiomers of the guest (Supplementary Table 2). The *R*/*S* ratios were also reflected in the integrals of the signals of the encapsulated corannulene (Fig. 4). All three elements of the system—the two guests and the host—thus collectively constructed and reported upon the chirotopic cavity environment.

The concave panels of host **1** enabled it to bind fullerenes well, reconfiguring stereochemically in order to optimize host–guest contact. The chirotopic space within **1** enabled the exploration of its stabilization of the bowl-shaped ground state of corannulene, which in turn served to “pad” the cavity in order to optimize the binding of cycloalkanes in ternary complexes. Ternary complexes involving chiral guests can report upon the guest’s stereochemistry. The ability of the guests to influence each other’s dynamics suggests novel applications where guest motions might be geared together—for example, the ring-flipping of corannulene might compress a second guest, thus accelerating a reaction with an unfavorable volume of activation, such as an intramolecular cycloaddition.

## Supplementary information

Supplementary Information

## Data Availability

The authors declare that all data supporting the findings of this study are included within the article and its Supplementary Information, and are also available from the authors upon request. Crystallographic data for the structures reported in this paper have been deposited at the Cambridge Crystallographic Data Center, under the deposition numbers 2068668 (**1**), 2068666 (C_60_⊂**1**), 2068667 (C_70_⊂**1**), and 2068665 (C_6_H_12_•corannulene⊂**1**). Copies of these data can be obtained free of charge via www.ccdc.cam.ac.uk/data_request/cif.
