# Supplementary material for: A curved host and second guest cooperatively inhibit the dynamic motion of corannulene
Source: Nat Commun. 2021 Jul 2;12:4079. doi: 10.1038/s41467-021-24344-w (PMC8253762; doi:10.1038/s41467-021-24344-w)
Supplement: Supplementary file 1 — Supplementary Information [file 41467_2021_24344_MOESM1_ESM.pdf]

Supplementary Information for

A curved host and second guest cooperatively inhibit the dynamic motion of corannulene

Yang Yang,[1,2] Tanya K. Ronson,[1] Zifei Lu,[1] Jieyu Zheng,[1] Nicolas Vanthuyne,[3] Alexandre Martinez,[3] Jonathan R. Nitschke[1,*]

1. Department of Chemistry, University of Cambridge, Lensfield Road, Cambridge CB2 1EW, UK.
2. School of Chemistry and Materials Science, Jiangsu Normal University, Xuzhou 221116, China
3. Aix Marseille Univ, CNRS, Centrale Marseille, iSm2, Marseille, France

[*]Corresponding Author: jrn34@cam.ac.uk

Table of Contents

1. **Materials and instrumentation**..2

2. **Synthesis and characterization**..3

   2.1 Synthesis of subcomponent A..3

   2.2 Synthesis of Cage **1**..5

   2.3 Host-guest complex with C_{60}..10

   2.4 Host-guest complex with C_{70}..16

   2.5 Host-guest chemistry of **1** with polyaromatic hydrocarbons..22

   2.6 Host-guest chemistry of **1** with corannulene and cycloalkanes as second guests..30

   2.7 Host-guest chemistry of **1** with corannulene and chiral 3-methyl-2-butanol as the second guest 42

3. **Determination of the binding Constants *K*a.**..44

   3.1 Binding constant for corannulene and pyrene..44

   3.2 Binding constant for hetero-guest systems..47

4. **Dynamic studies of the guests inside cage 1**..51

5. **Enantiomeric excess value determinations by NMR**..58

6. **Circular Dichroism studies**..61

7. **Volume calculations**..62

8. **Chiral resolution of subcomponent A and assignment of the configurations**..63

   8.1 Analytical chiral HPLC separation for compound **A**..63

   8.2 Preparative separation for compound **A**:..64

   8.3 Optical rotations..65

   8.4 Electronic Circular Dichroism..66

   8.5 Absolute configuration determination by comparison of calculated and experimental ECD spectra..67

9. **X-ray Crystallography**..70

1. Materials and instrumentation

Unless otherwise specified, all reagents were purchased from commercial sources and used as received. NMR spectra were recorded using Bruker Avance 500 MHz cryoprobe (^{13}C NMR, VT NMR), Bruker Avance 500 MHz TCI-ATM cryoprobe (^{1}H NMR for *e.e.* determination, NOESY) or 400 MHz Avance III HD (^{1}H NMR, VT NMR, EXSY) in deuterated acetonitrile and are reported relative to residual solvent signals. Chemical shifts for ^{1}H, ^{13}C, ^{31}P and ^{19}F NMR are reported in ppm on the δ scale; ^{1}H and ^{13}C were referenced to the residual solvent peak. Coupling constants (J) are reported in Hz.

DOSY experiments were performed on a 400 MHz Avance III HD smart probe spectrometer. Maximum gradient strength was 6.57 G/cmA. The standard Bruker pulse program, ledbpgp2s, employing a stimulated echo and longitudinal eddy-current delay (LED) using bipolar gradient pulses for diffusion was utilized. Rectangular gradients were used with a total duration of 1.5 ms. Gradient recovery delays were 1200 μs. Individual rows of the S4 quasi-2D diffusion databases were phased and baseline corrected. The EXSY experiments were performed on a 400 MHz Avance III HD smart probe spectrometer. The standard Bruker pulse program, noesygpphpp, was applied. The mixing time D8 was adjusted to be 300 ms.

Circular Dichroism was performed on an Applied-Photophysics Chirascan CD spectrometer using a 1 mm path-length cuvette. Experiments were recorded at 298 K, maintained with a Peltier temperature control. Measurements were background subtracted from blank solvent in an identical cuvette. The sample concentrations were adjusted to maintain a HV below 700 V. A minimum sample integration time of 1 second was used.

Low resolution electrospray ionization mass spectrometry was undertaken on a Micromass Quattro LC mass spectrometer (cone voltage 10-30 eV; desolvation temp. 40 °C; ionization temp. 40 °C) infused from a Harvard syringe pump at a rate of 10 $\mu L\cdot min^{-1}$. High-resolution ESI mass spectra were obtained using a Thermo Scientific LTQ Orbitrap XL hybrid ion trap-orbitrap mass spectrometer.

2. Synthesis and characterization

2.1 Synthesis of subcomponent A.

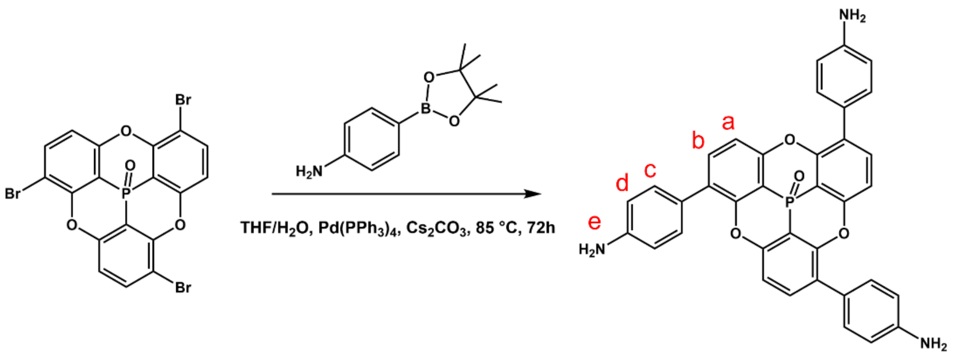

The precursor *rac*-tribromophosphangulene was synthesized based on reported methods.[1] A suspension of *rac*-tribromophosphangulene (200 mg, 0.360 mmol, 1 equiv), 4-aminophenylboronic acid pinacol ester (316 mg, 1.44 mmol, 4 equiv) and Cs_2CO_3 (352 mg, 1.08 mmol, 3 equiv) in THF/H_2O (3:1, 150 mL) was degassed with nitrogen for 15 minutes. $Pd(PPh_3)_4$ (83 mg, 0.072 mmol, 0.20 equiv) was added and the mixture was heated at 85 °C for 72 hours under nitrogen. After cooling to room temperature, the mixture was extracted with 100 mL dichloromethane 3 times. The combined organic layers were washed with brine, and dried over $MgSO_4$ and the solvent was evaporated. The crude product was purified by flash column, with mixed solvent dichloromethane/ethanol (100:1) as eluent. About 95 mg of ligand **A** was obtained. Yield: 44.5%. ^{1}H NMR (400 MHz, 298 K, $CDCl_3$, ppm): δ = 7.46 (d, J = 8.0 Hz, 3H, H_b), 7.40 (d, J = 8.0 Hz, 6H, H_c), 7.10 (dd, J_{H-H} = 8.0 Hz, J_{H-P} = 4.8 Hz, 3H, H_a), 7.78 (d, J = 8.0 Hz, 6H, H_d), 5.30 (s, 4H, H_e). ^{31}P NMR (162 MHz, 298 K, $CDCl_3$, ppm): δ = -46.14. ^{13}C NMR (101 MHz, 298 K, $CDCl_3$, ppm): δ = 158.2, 155.5, 145.9,133.4, 130.0, 128.8, 125.1, 114.9, 114.6,110.4, 109.3. HR-MS found: 594.1602 $[M+H]^+$; $C_{36}H_{25}N_3O_4P$ requires: 594.1577.

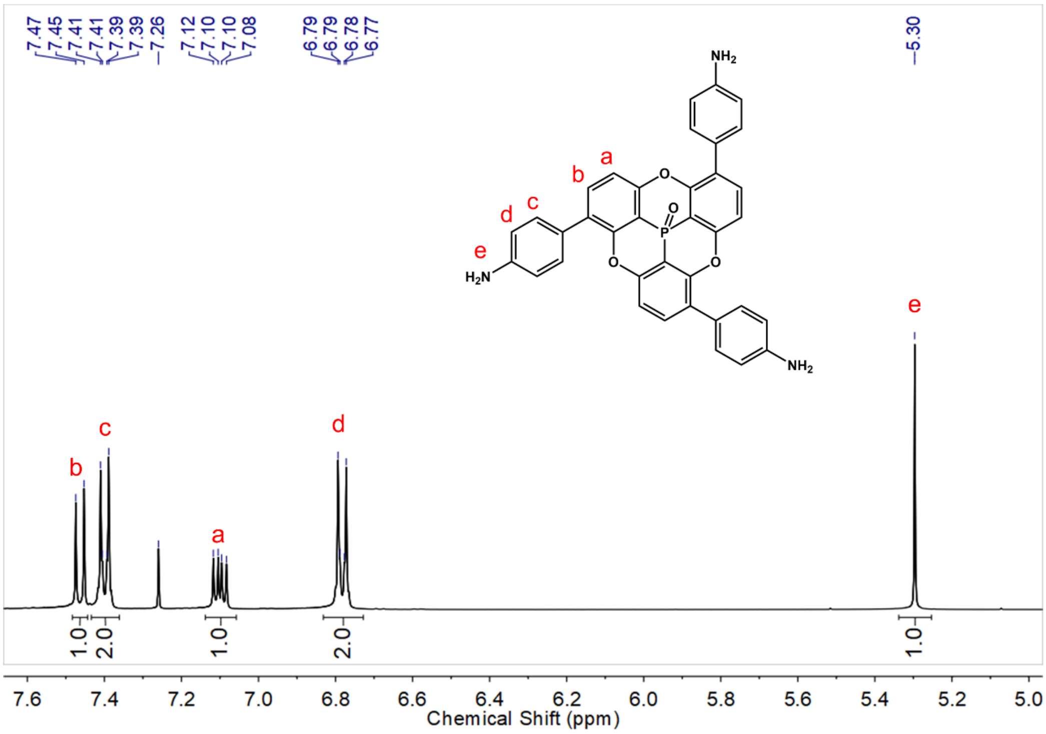

Supplementary Figure 1. ^{1}H NMR spectrum of **A** in $CDCl_3$ (400 MHz, 298 K).

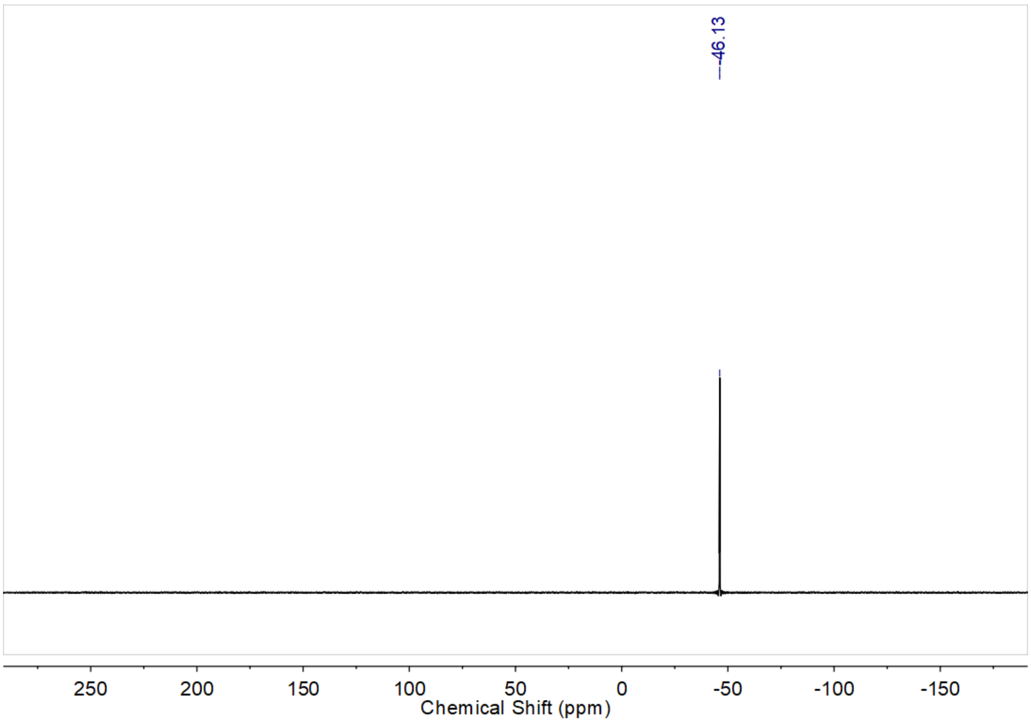

Supplementary Figure 2. ^{31}P NMR spectrum of **A** in $CDCl_3$ (298 K).

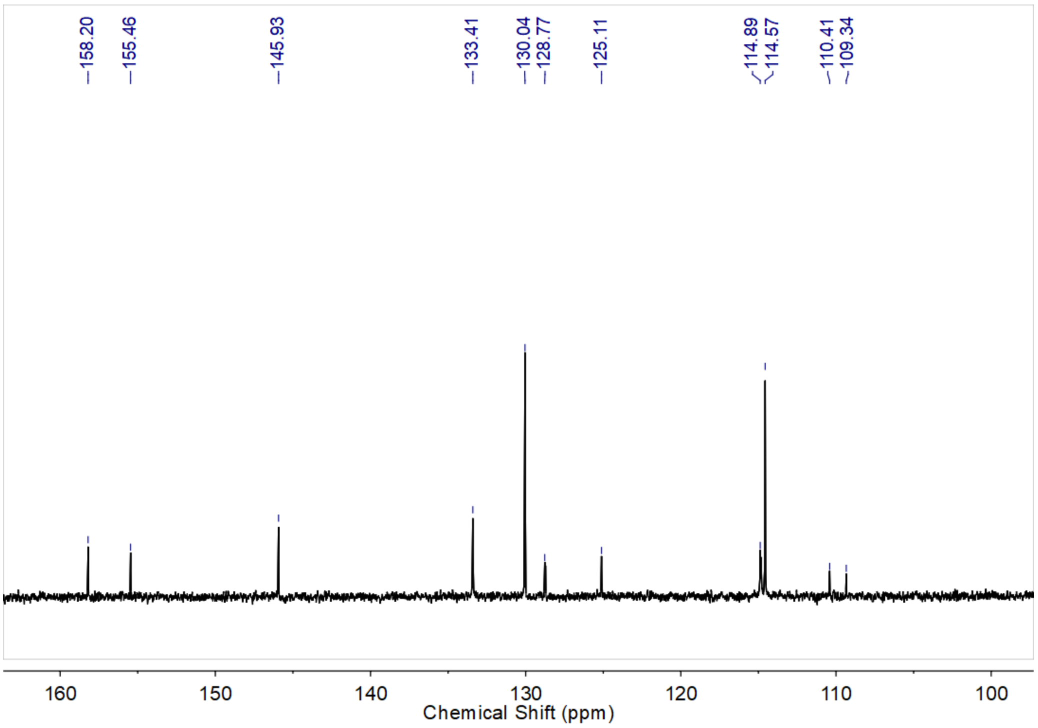

Supplementary Figure 3. ^{13}C NMR spectrum of **A** in $CDCl_3$ (298 K).

2.2 Synthesis of Cage 1

Zinc(II) triflimide (21 mg, 0.034 mmol), subcomponent **A** (20mg, 0.034 mmol) and 2-formylpyridine (10 µL, 0.105 mmol) were dissolved in acetonitrile (5 mL). The reaction mixture was stirred at 70 °C overnight. The volume was reduced to 1 mL *in vacuo*. To the solution was added excess diethyl ether to precipitate the yellow crude product. The crude product was dissolved in 0.5 mL acetonitrile and precipitated by addition of excess diethyl ether again. The precipitate was further washed with diethyl ether. The product was dried *in vacuo*. Yield ca. 40 mg, 80%. The syntheses of P_4-Λ_4 **1** and M_4-Δ_4 **1** are the same as cage **1**, except for the use of the first eluted (*P*) or second eluted (*M*) enantiomer of subcomponent **A** instead of racemic **A**. ^{1}H NMR (400 MHz, 298 K, CD_3CN, ppm): δ = 8.69 (s, H = 24, H_e), 8.51(t, J = 7.6 Hz, H = 24, H_g), 8.17 (d, J = 7.6 Hz, H = 24, H_f), 8.16 (d, J = 4.8 Hz, H = 24, H_i), 7.96 (dd, J_1 = 7.6 Hz, J_2 =5.2 Hz, H = 24, H_h), 7.34-7.41 (m, H = 72, H_b and H_c), 6.90 (dd, $J_{H\text{-}H}$ = 8.8 Hz, $J_{P\text{-}H}$ = 4.8 Hz, H = 24, H_a), 6.31 (d, J = 8.8 Hz, H = 48, H_d). ^{31}P NMR (162M, 298 K, CD_3CN, ppm): δ = -47.52. ^{19}F NMR (376.5 Hz, 298 K, CD_3CN, ppm): δ = -81.12, -82.83. ^{13}C NMR (125 MHz, 298 K, CD_3CN, ppm):165.1, 159.4, 155.6, 149.6, 147.5, 146.5, 142.8, 135.7, 135.0, 130.9(d), 128.4(d), 121.2,

121.0,118.6, 115.5, 110.6, 109.8. ESI-MS (m/z): 463.1 ([**1**-8NTf$_2$]$^{8+}$), 569.2 ([**1**-7Ntf$_2$]$^{7+}$), 710.6 ([**1**-6Ntf$_2$]$^{6+}$), 908.9 ([**1**-5Ntf$_2$]$^{5+}$).

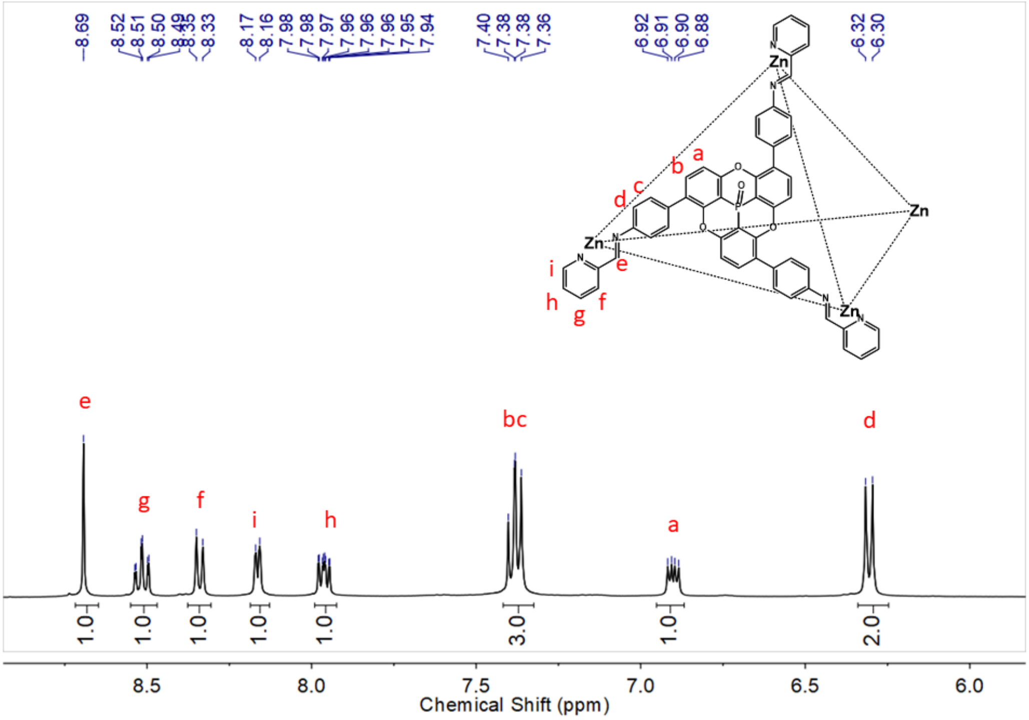

Supplementary Figure 4. ^{1}H NMR spectrum of **1** in CD_3CN (400 MHz, 298 K).

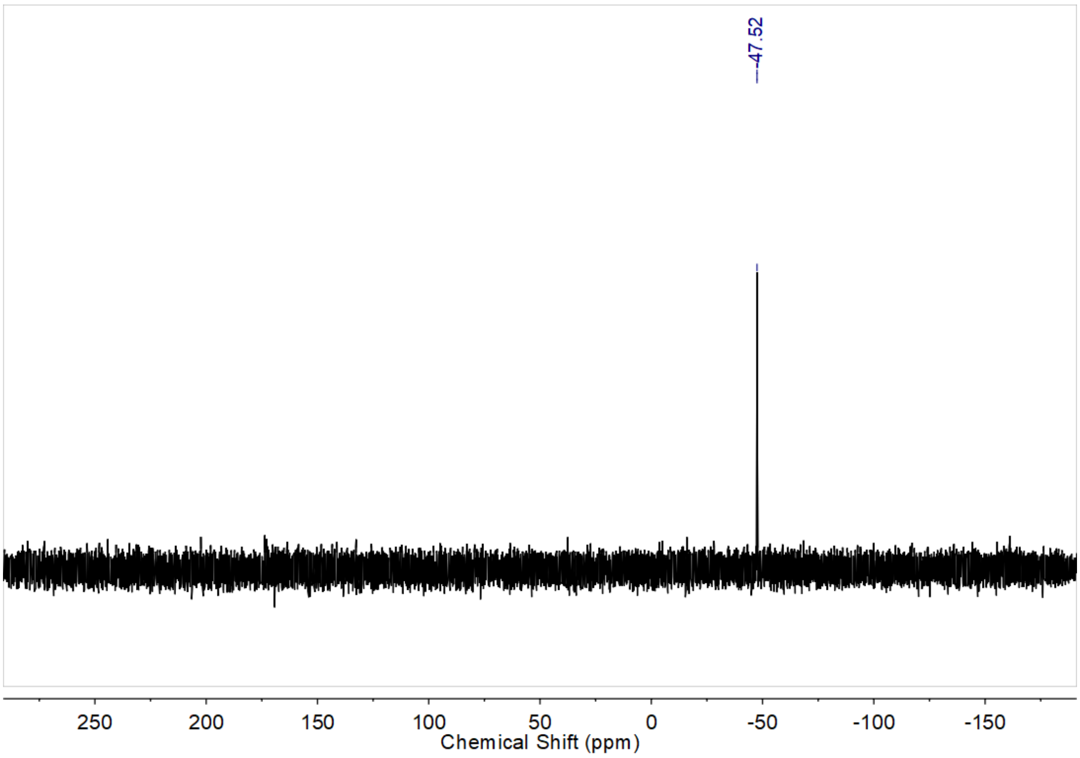

Supplementary Figure 5. ^{31}P NMR spectrum of **1** in CD_3CN (298 K).

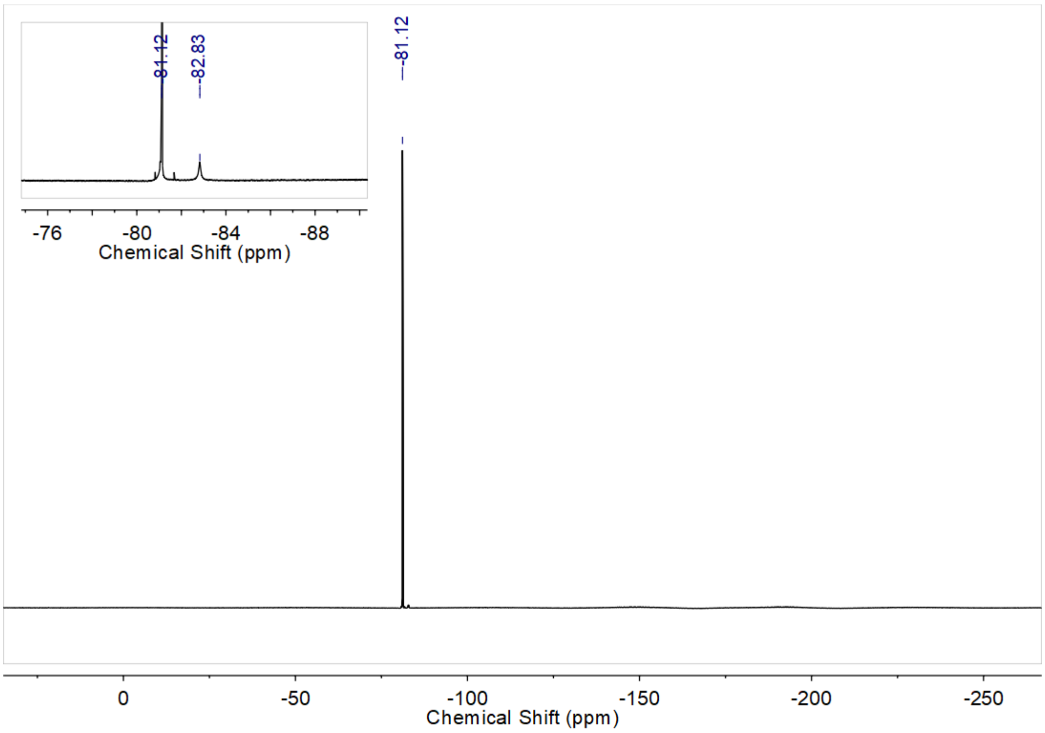

Supplementary Figure 6. ^{19}F NMR spectrum of **1** in CD_3CN (298 K) with inset showing the peak for encapsulated triflimide.

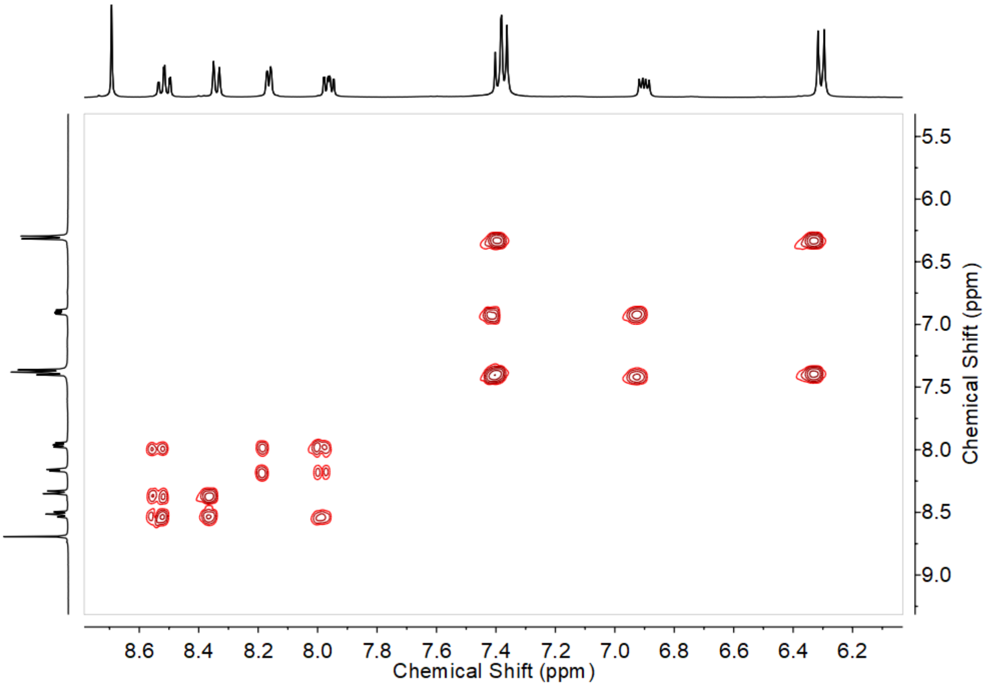

Supplementary Figure 7. ^{1}H-^{1}H COSY spectrum of **1** in CD_3CN (400 MHz, 298 K).

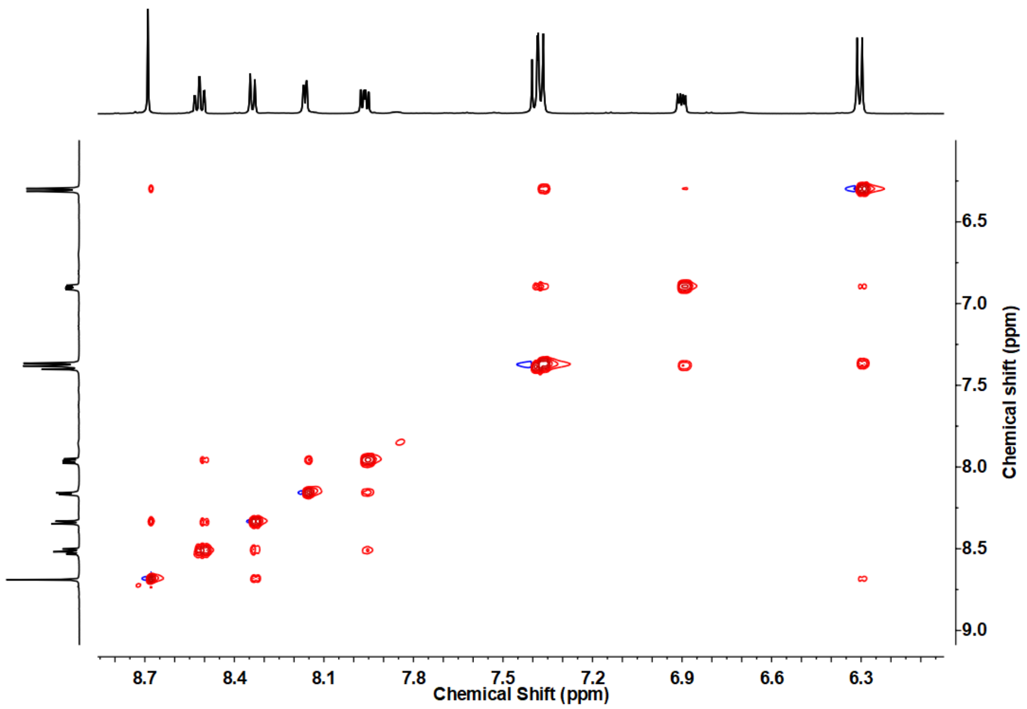

Supplementary Figure 8. ^{1}H-^{1}H NOESY spectrum of **1** in CD_3CN (500 MHz, 298 K).

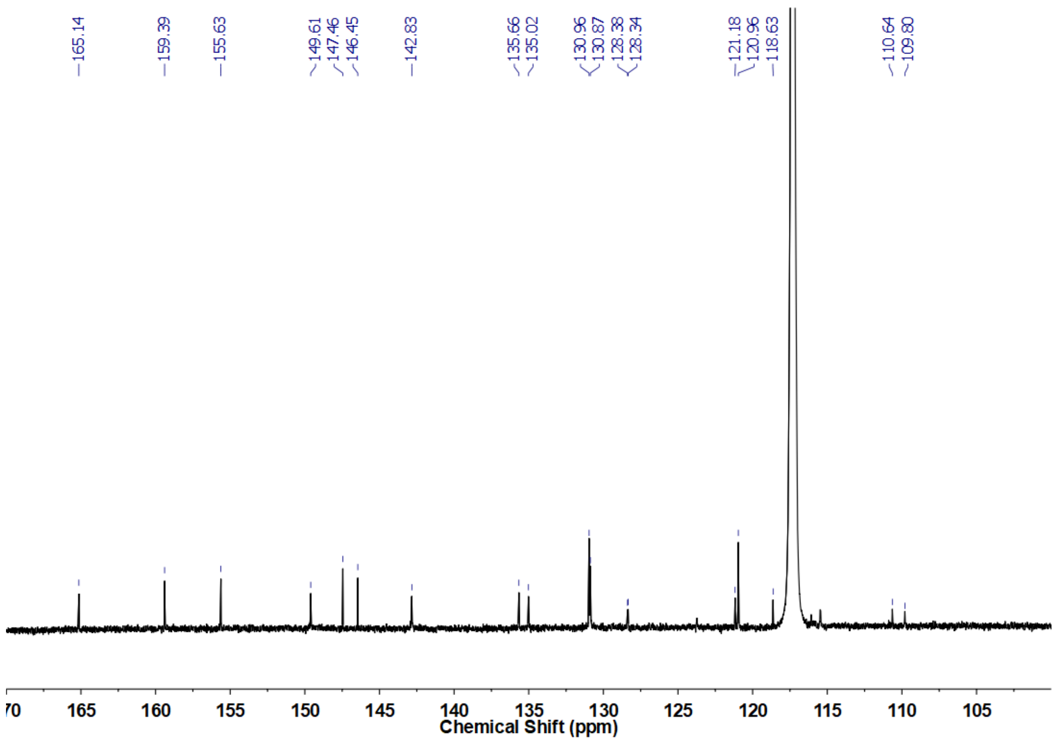

Supplementary Figure 9. ^{13}C NMR spectrum of **1** in CD_3CN (298 K).

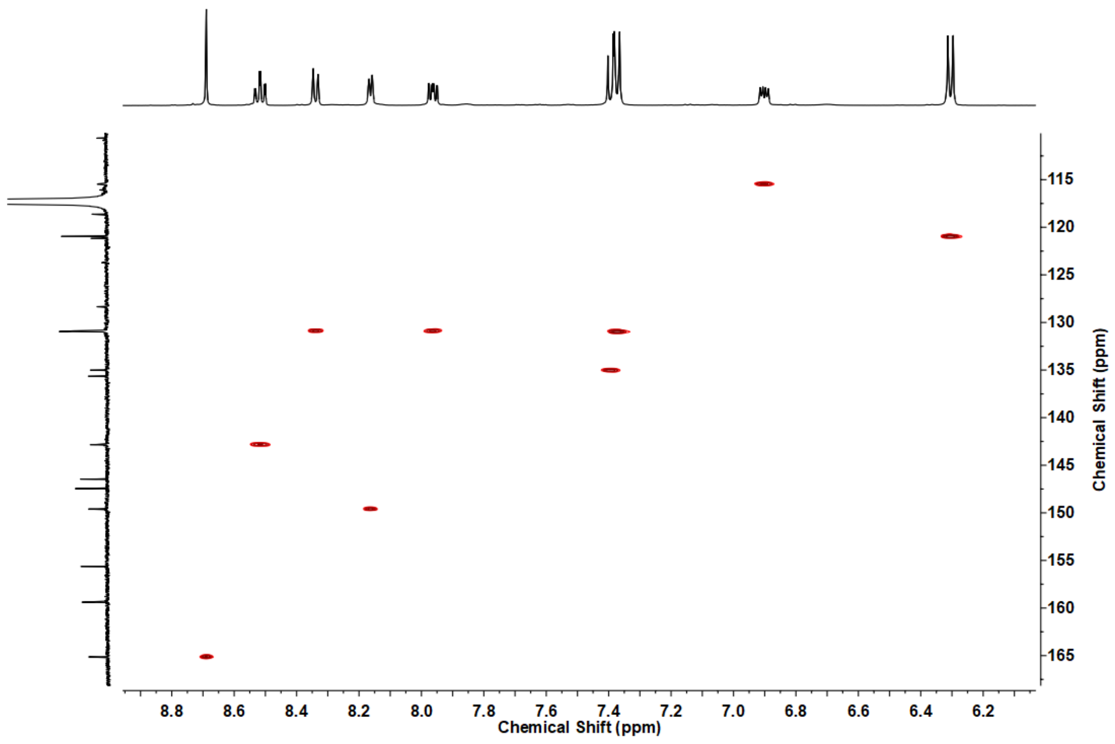

Supplementary Figure 10. ^{1}H-^{13}C HSQC spectrum of **1** in CD_3CN (298 K).

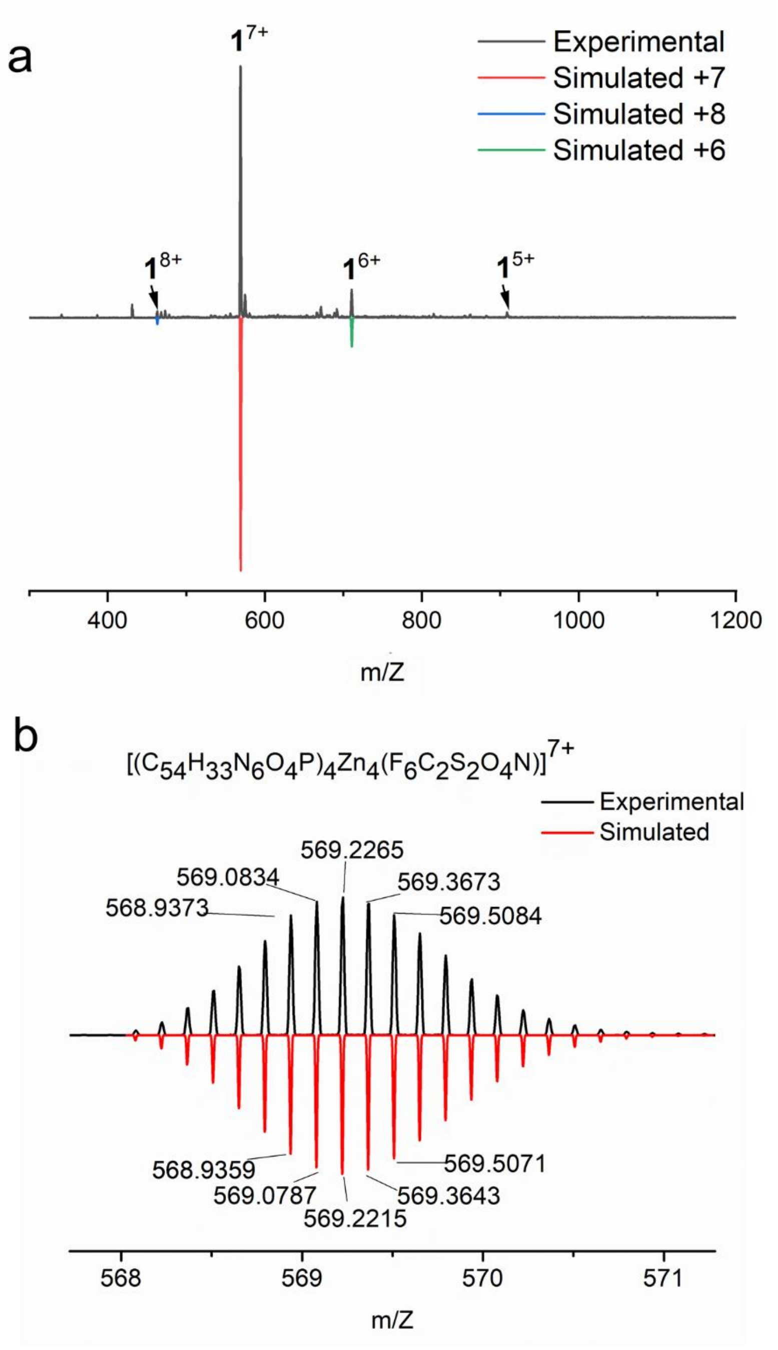

Supplementary Figure 11. ESI-MS spectra of **1**. a) Low-resolution ESI-MS spectrum of **1**. The coloured peaks are simulated isotopic ion peaks of **1** with loss of corresponding numbers of counter ions. b) The +7 peaks of high-resolution ESI-MS of **1**.

2.3 Host-guest complex with C_{60}.

In situ assembly: Zinc(II) triflimide (2.1 mg, 0.0034 mmol), subcomponent **A** (2.0 mg, 0.0034 mmol) and 2-formylpyridine (1.0 μL, 0.0105 mmol) and fullerene C_{60} (3.0 mg, 0.00417 mmol) were mixed in

CD_3CN (0.5 mL). The mixture was kept at 70 °C overnight. The sample was centrifuged and filtered to remove the insoluble excess C_{60}. The sample was kept at 70 °C for a further week and monitored by ^1H NMR. After this time the sample contained only the $\boldsymbol{T}_2$ configuration. Diethyl ether was layered on the sample in an NMR tube. After two weeks crystals formed. The NMR yield was 100%, and the isolated yield for the crystals was ca. 2.5 mg, 45%. ^1H NMR (500 MHz, 298 K, CD_3CN, ppm): δ = 8.79 (s, H = 24, H_e), 8.54(td, J_1 = 8.0 Hz, J_2 = 1.5, H = 24, H_g), 8.40 (d, J = 7.5 Hz, H = 24, H_f), 8.05 (d, J = 5.0 Hz, H = 24, H_i), 7.96 (dd, J_1 = 7.5 Hz, J_2 =5.0 Hz, H = 24, H_h), 7.80 (d, J = 8.5 Hz, H = 48, H_b), 7.47-7.53 (m, H = 72, H_c and H_a), 6.53 (d, J = 8.5 Hz, H = 48, H_d). ^{13}C NMR (125 MHz, 298 K, CD_3CN, ppm): 164.9, 158.2, 155.4, 149.7, 147.0, 146.2, 142.9, 140.6 (encapsulated C_{60}), 135.0, 134.7, 131.4, 131.0, 130.6, 127.9, 121.6, 121.2, 118.7, 116.0, 109.9, 109.1. ESI-MS (m/z): 553.0 ($[C_{60}\subset\mathbf{1}\text{-}8NTf_2]^{8+}$), 672.1 ($[C_{60}\subset\mathbf{1}\text{-}7Ntf_2]^{7+}$), 830.8 ($[C_{60}\subset\mathbf{1}\text{-}6Ntf_2]^{6+}$), 1052.9 ($[C_{60}\subset\mathbf{1}\text{-}5Ntf_2]^{5+}$).

Reaction of C_{60} with pre-formed cage **1**: 2 mg cage **1** was mixed with 1.5 mg fullerene C_{60} in 0.5 mL CD_3CN. The sample was kept at 70 °C for 72 days and monitored by ^1H NMR.

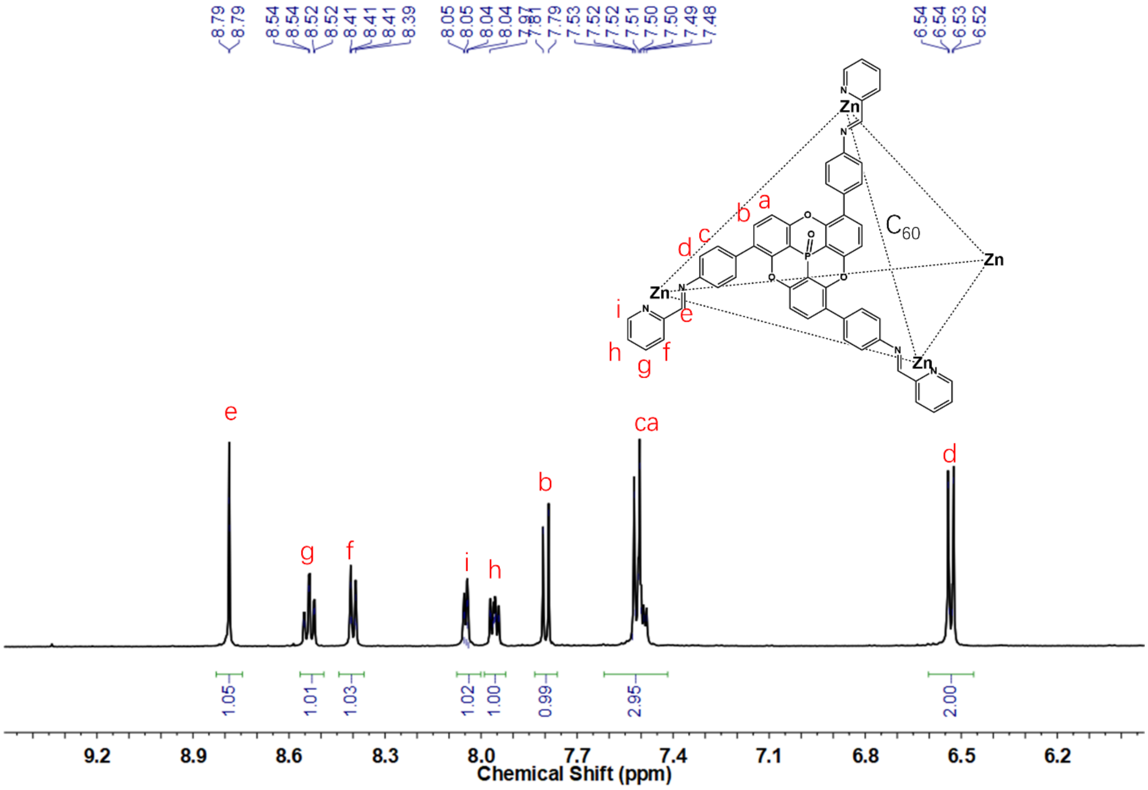

Supplementary Figure 12. ^1H NMR spectrum of $C_{60}\subset\mathbf{1}$ in CD_3CN (500 MHz, 298 K).

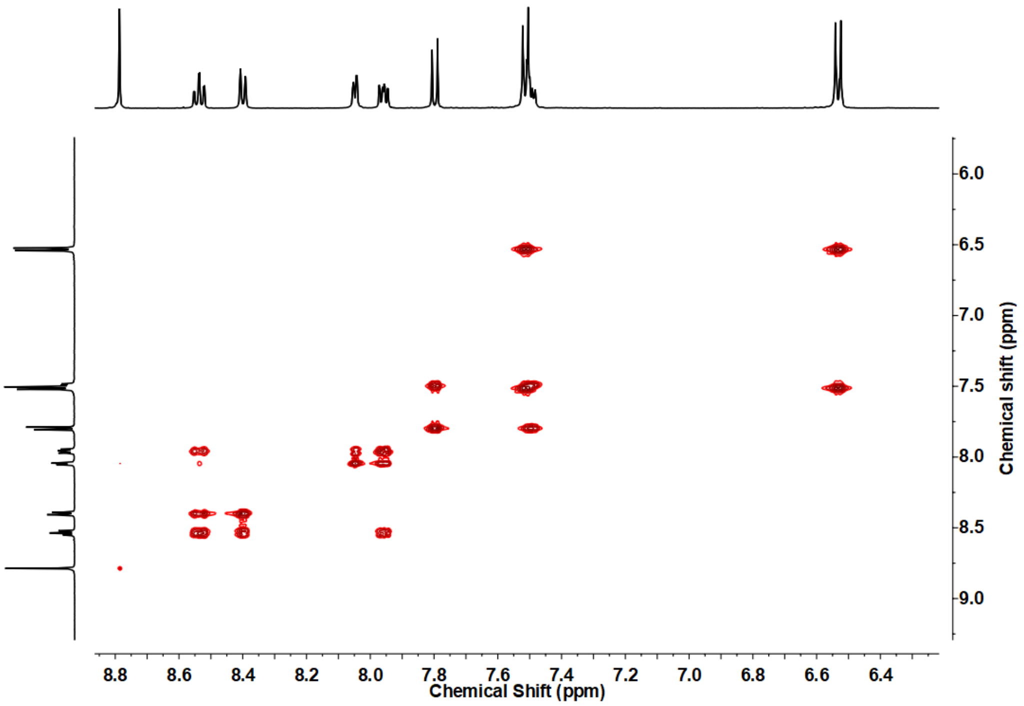

Supplementary Figure 13. ^{1}H-^{1}H COSY spectrum of $C_{60}\subset$**1** in CD_3CN (298 K).

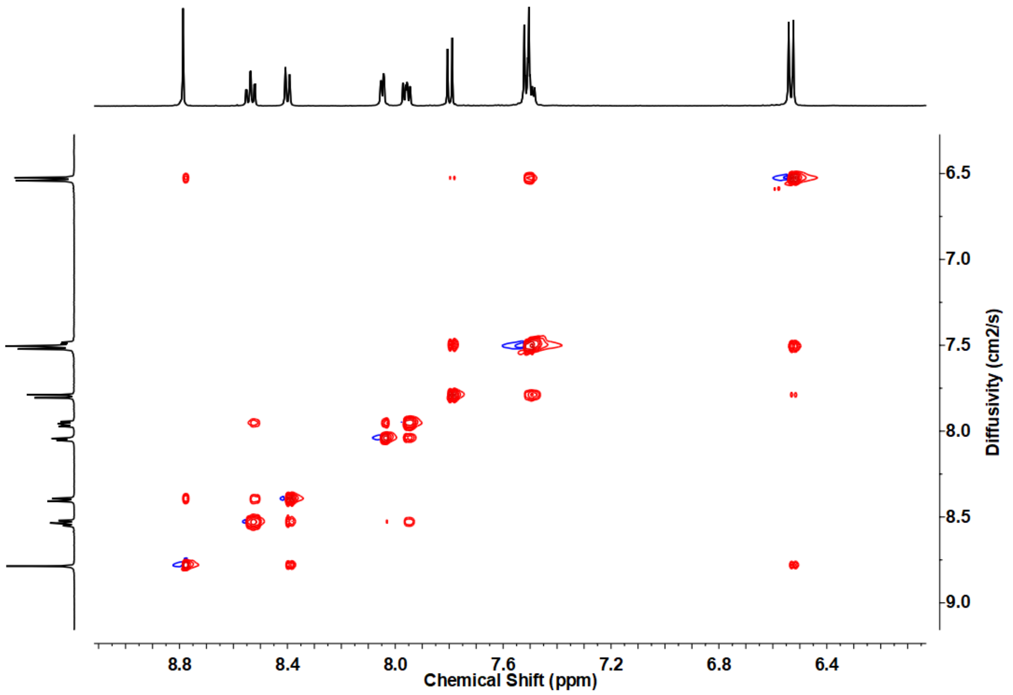

Supplementary Figure 14. ^{1}H-^{1}H NOESY spectrum of $C_{60}\subset$**1** in CD_3CN (298 K).

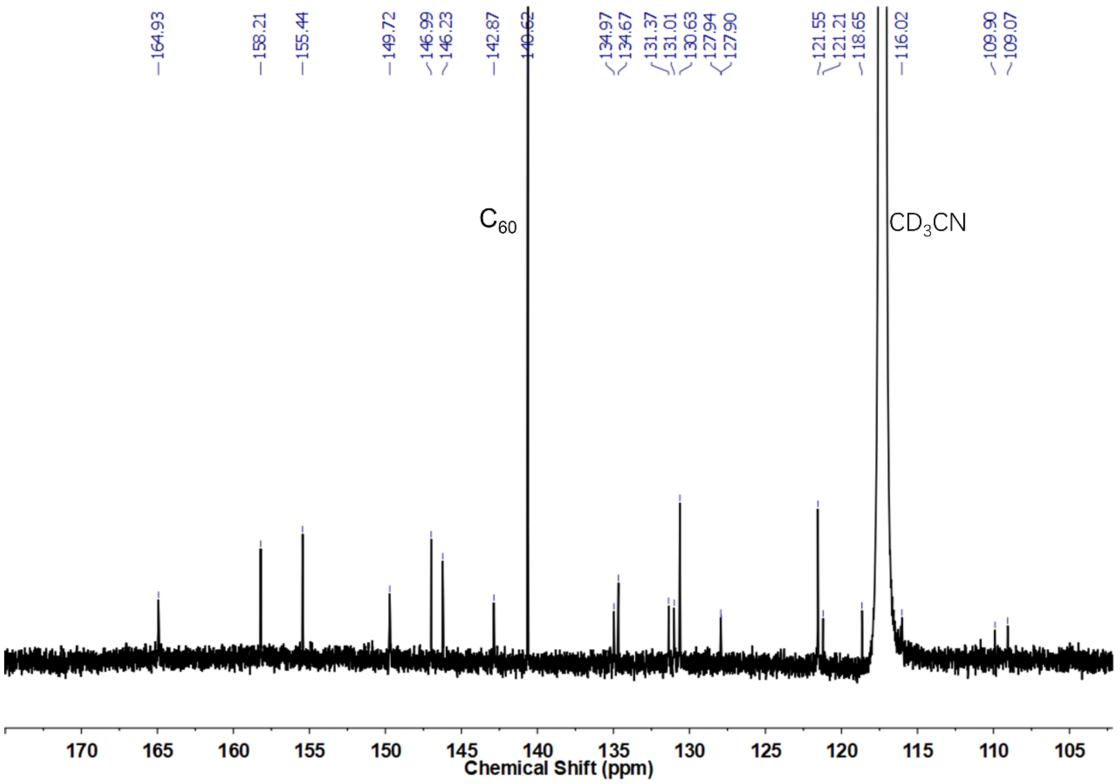

Supplementary Figure 15. ^{13}C spectrum of C_{60}⊂**1** in CD_3CN (298 K).

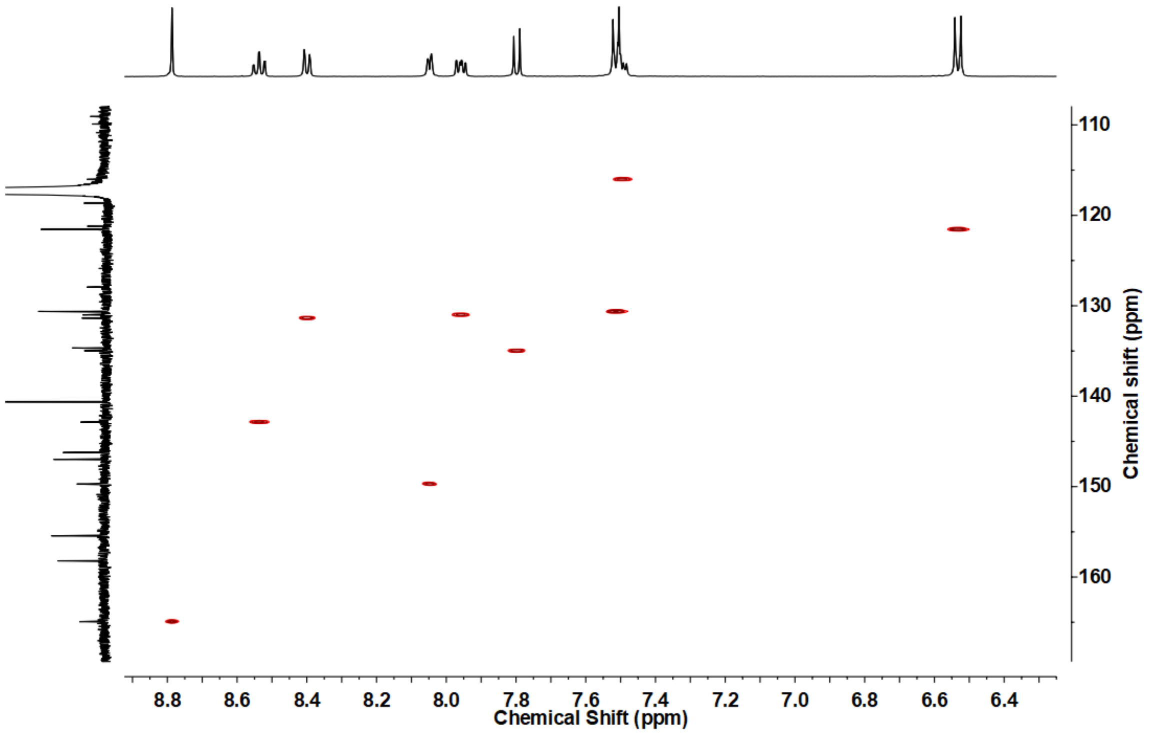

Supplementary Figure 16. ^{1}H-^{13}C HSQC spectrum of C_{60}⊂**1** in CD_3CN (298 K).

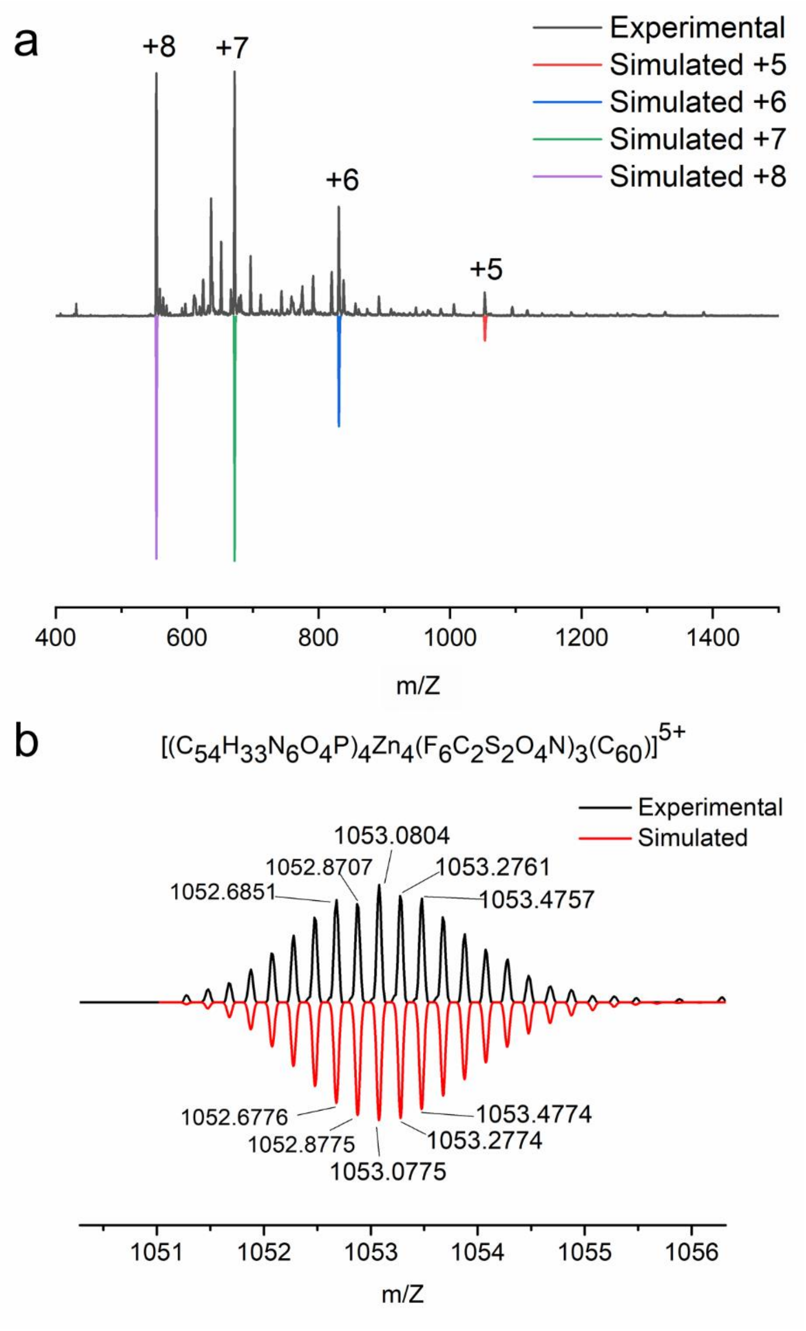

Supplementary Figure 17. ESI-MS spectra of C_{60}⊂**1**. a) Low-resolution ESI-MS spectrum of C_{60}⊂**1**. The coloured peaks are simulated isotopic ion peaks of C_{60}⊂**1** with loss of corresponding numbers of counter ions. b) The +5 peaks of high-resolution ESI-MS of C_{60}⊂**1**.

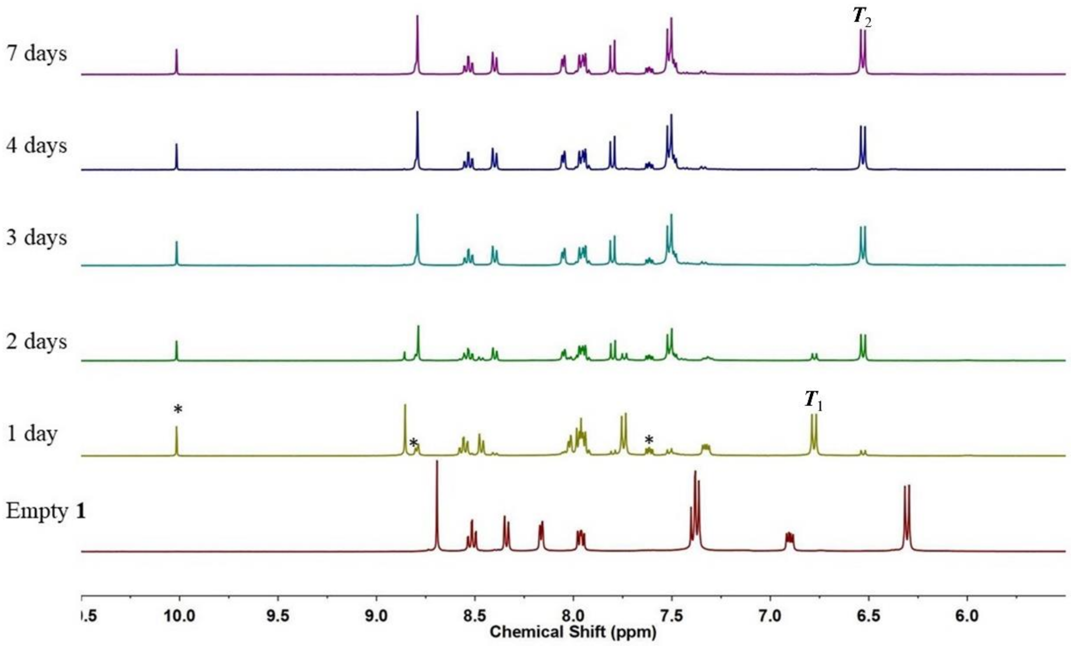

Supplementary Figure 18. Time-dependent ^{1}H NMR spectra of *in situ* assembly of sub-components with C_{60} as guest and excess 2-formylpyridine. The reaction was kept at 70°C except during NMR measurements. The empty cage **1** is shown for reference. The asterisks (*) represent excess 2-formylpyridine.

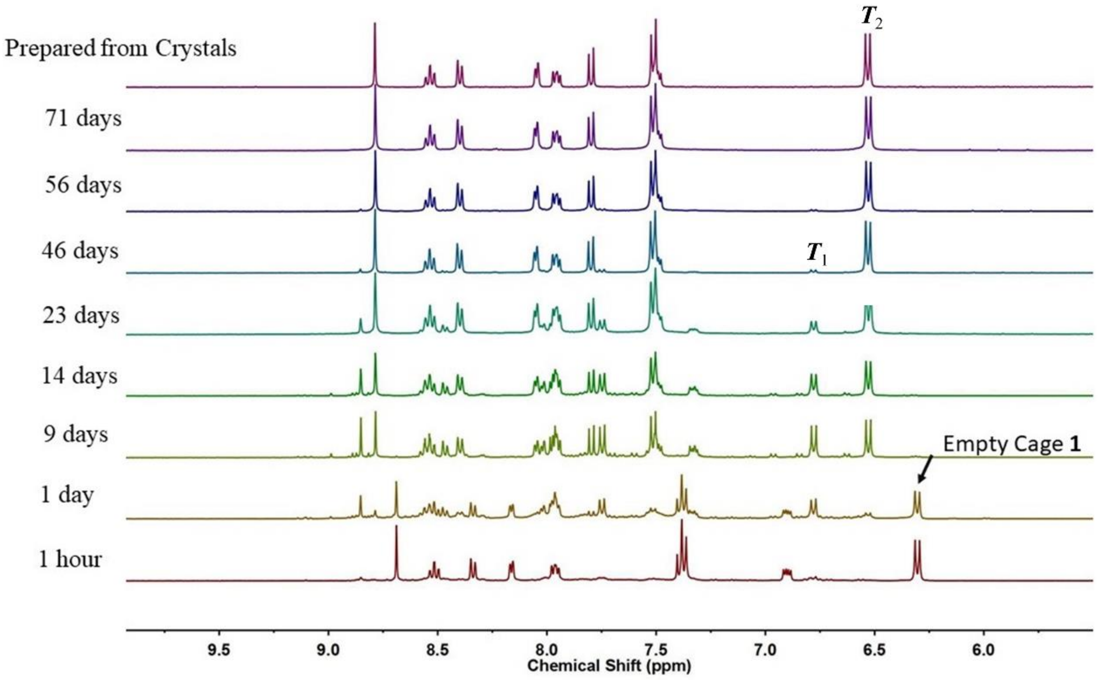

Supplementary Figure 19. Time-dependent ^{1}H NMR spectra of the reaction solution of preformed Cage **1** with C_{60}. The reaction was kept at 70°C except during NMR measurement. In comparison to

the *in situ* assembly, the preformed cages reacting with C_{60} required a longer time for encapsulation and stereoisomeric transformation.

2.4 Host-guest complex with C_{70}.

In situ assembly: Zinc(II) triflimide (2.1 mg, 0.0034 mmol), subcomponent **A** (2.0mg, 0.0034 mmol) and 2-formylpyridine (1.0 µL, 0.0105 mmol) and fullerene C_{70} (3.0 mg, 0.00357 mmol) were mixed in CD_3CN (0.5 mL). The mixture was kept at 70 °C for 24 hours. The sample was centrifuged and filtered to remove the insoluble excess C_{70}. The sample was checked by ^{1}H NMR. Diethyl ether was layered on the sample in NMR tube. After two weeks crystals formed on the tube walls. The NMR yield was 100%, and the isolated yield of the crystals was ca. 3.5 mg, 61%. ^{1}H NMR (500 MHz, 298 K, CD_3CN, ppm): δ = 8.88 (s, H = 24, H_e), 8.51(td, J_1 = 7.5 Hz, J_2 = 1.5, H = 24, H_g), 8.38 (d, J = 8.0 Hz, H = 24, H_f), 7.97 (d, J = 5.0 Hz, H = 24, H_i), 7.92 (dd, J_1 = 7.5 Hz, J_2 =5.0 Hz, H = 24, H_h), 7.62 (d, J = 8.5 Hz, H = 48, H_b), 7.44 (dd, $J_{H\text{-}H}$ = 8.5 Hz, $J_{P\text{-}H}$ = 4.5 Hz, H = 24, H_a), 6.85 (d, J = 9.0 Hz, H = 48, H_d). ^{13}C NMR (125 MHz, 298 K, CD_3CN, ppm): 164.6, 158.4, 155.3, 149.5, 148.1 (encapsulated C_{70a}), 146.6, 146.2, 146.6(encapsulated C_{70c}), 146.5 (encapsulated $C_{70c`}$), 144.80 (encapsulated $C_{70b}$), 143.0 (encapsulated $C_{70d}$), 142.9 (encapsulated $C_{70d`}$), 142.78, 131.41, 131.00, 130.90,128.41,127.54, 121.80, 121.20, 118.65, 116.58,110.00, 109.15. ESI-MS (m/z): 568.1 ([C_{70}⊂**1**-8NTf$_2$]$^{8+}$), 689.3 ([C_{70}⊂**1**-7NTf$_2$]$^{7+}$), 850.8 ([C_{70}⊂**1**-6NTf$_2$]$^{6+}$), 1077.2 ([C_{70}⊂**1**-5NTf$_2$]$^{5+}$).

Reaction of C_{70} with pre-formed cage **1**: 2 mg cage **1** was mixed with 1.5 mg fullerene C_{70} in 0.5 mL CD_3CN. The sample was kept at 70 °C for 9 days and monitored by ^{1}H NMR.

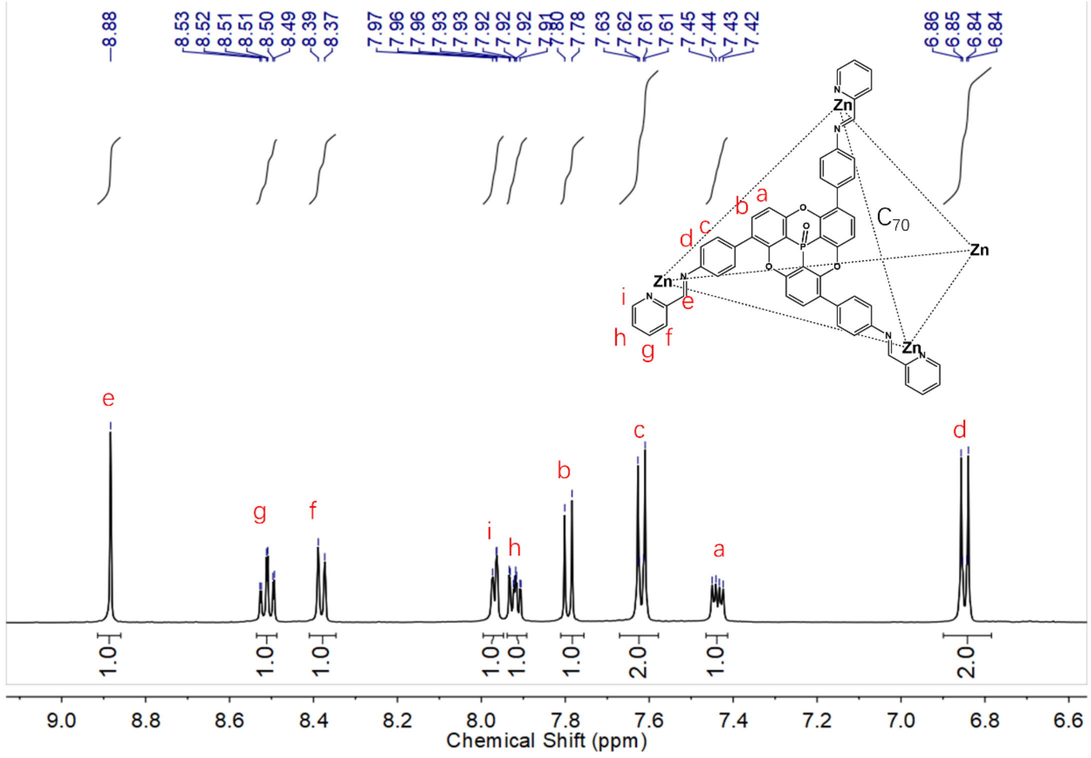

Supplementary Figure 20. ^{1}H NMR spectrum of C_{70}⊂**1** in CD_3CN (500 MHz, 298 K).

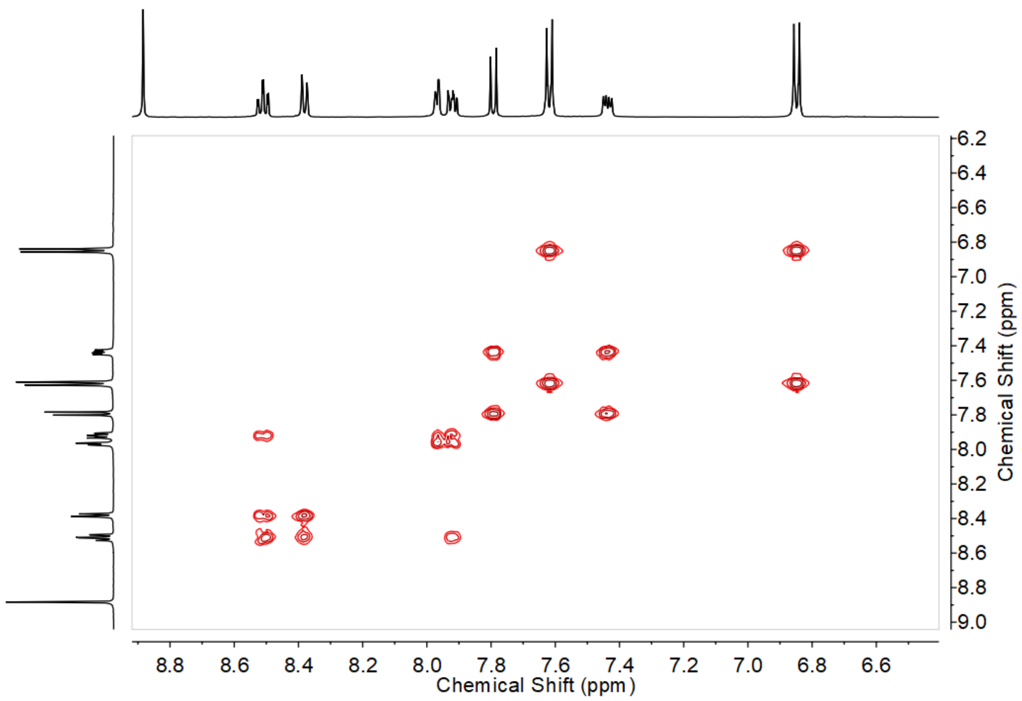

Supplementary Figure 21. ^{1}H-^{1}H COSY spectrum of C_{70}⊂**1** in CD_3CN (298 K).

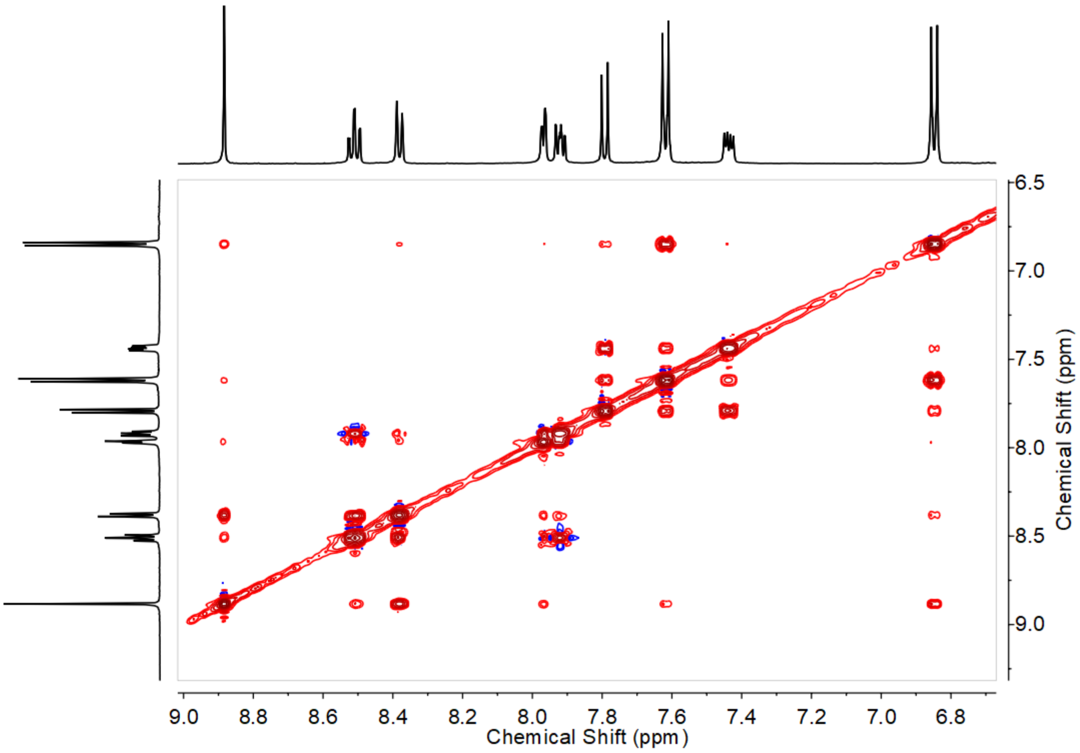

Supplementary Figure 22. ^{1}H-^{1}H NOESY spectrum of C_{70}⊂**1** in CD_3CN (298 K).

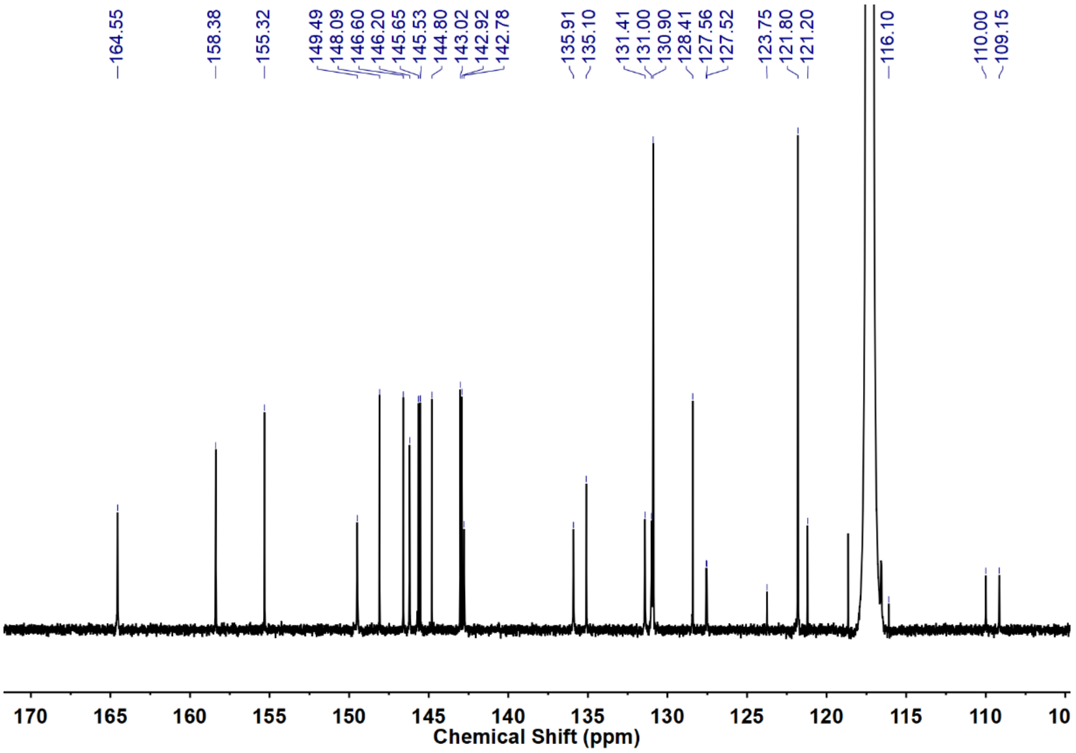

Supplementary Figure 23. ^{13}C spectrum of $C_{70}\subset$**1** in CD_3CN (298 K).

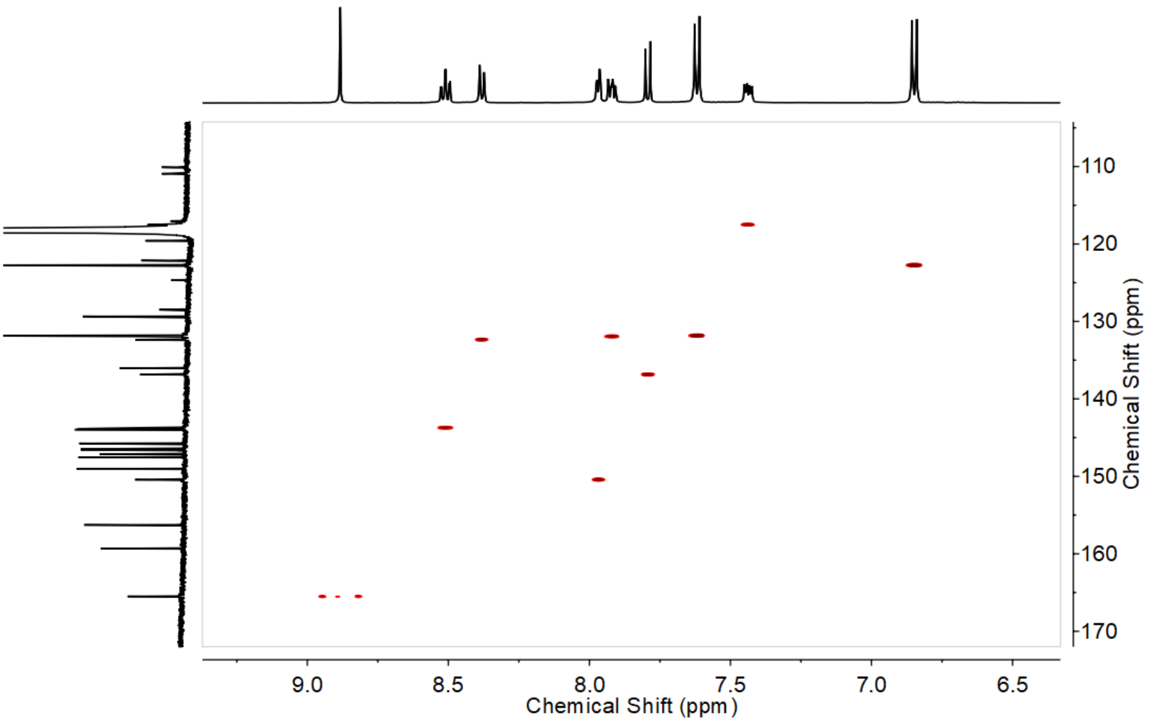

Supplementary Figure 24. ^{1}H-^{13}C HSQC spectrum of $C_{70}\subset$**1** in CD_3CN (298 K).

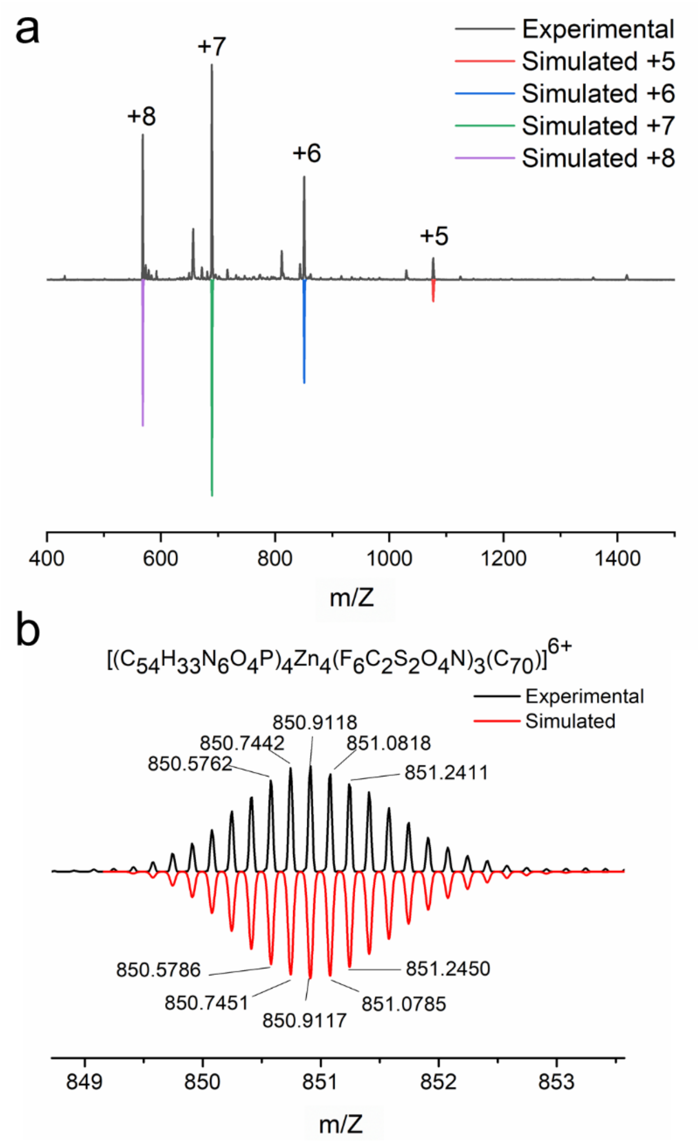

Supplementary Figure 25. ESI-MS spectra of C_{70}⊂**1**. a) Low-resolution ESI-MS spectrum of C_{70}⊂**1**. The coloured peaks are simulated isotopic ion peaks of C_{70}⊂**1** with loss of corresponding numbers of counter ions. b) The +6 peaks of high-resolution ESI-MS of C_{70}⊂**1**.

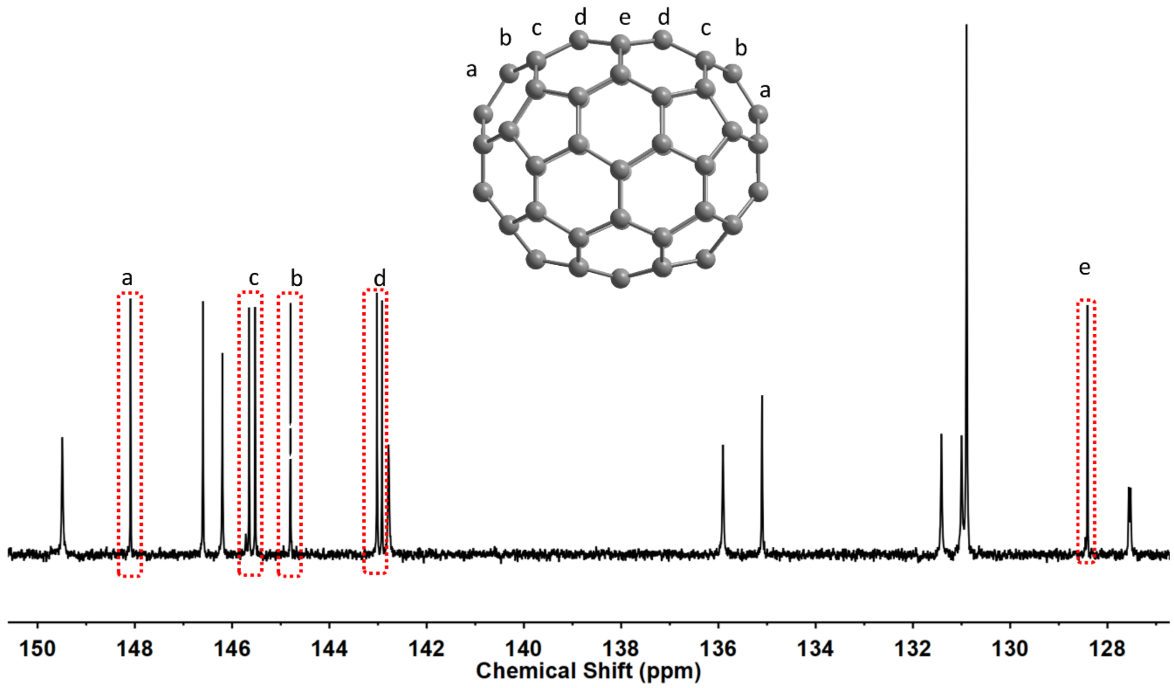

Supplementary Figure 26. Detailed assignment of ^{13}C NMR spectrum of C_{70}⊂**1** in CD_3CN with peaks of encapsulated C_{70} labelled by red boxes.

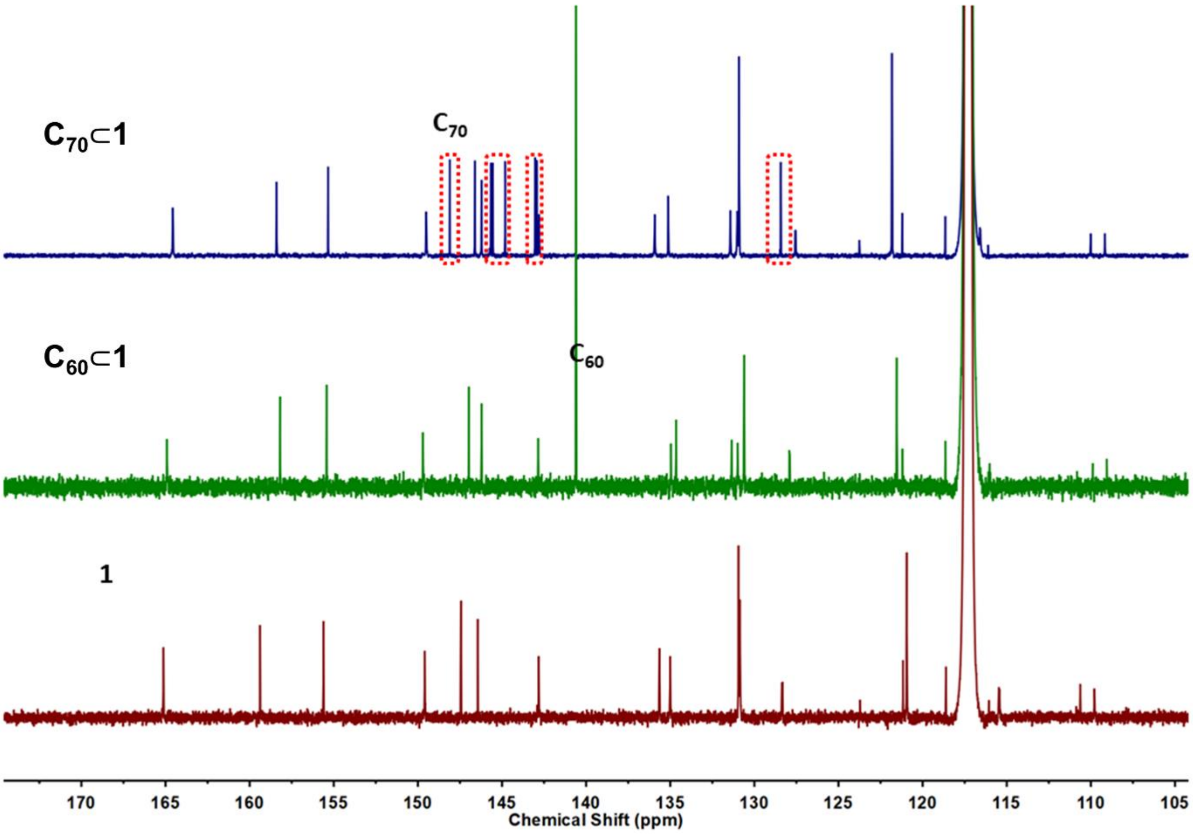

Supplementary Figure 27. Comparison of ^{13}C NMR spectra of **1**, **C_{60}⊂1**, **C_{70}⊂1** in CD_3CN (298K)

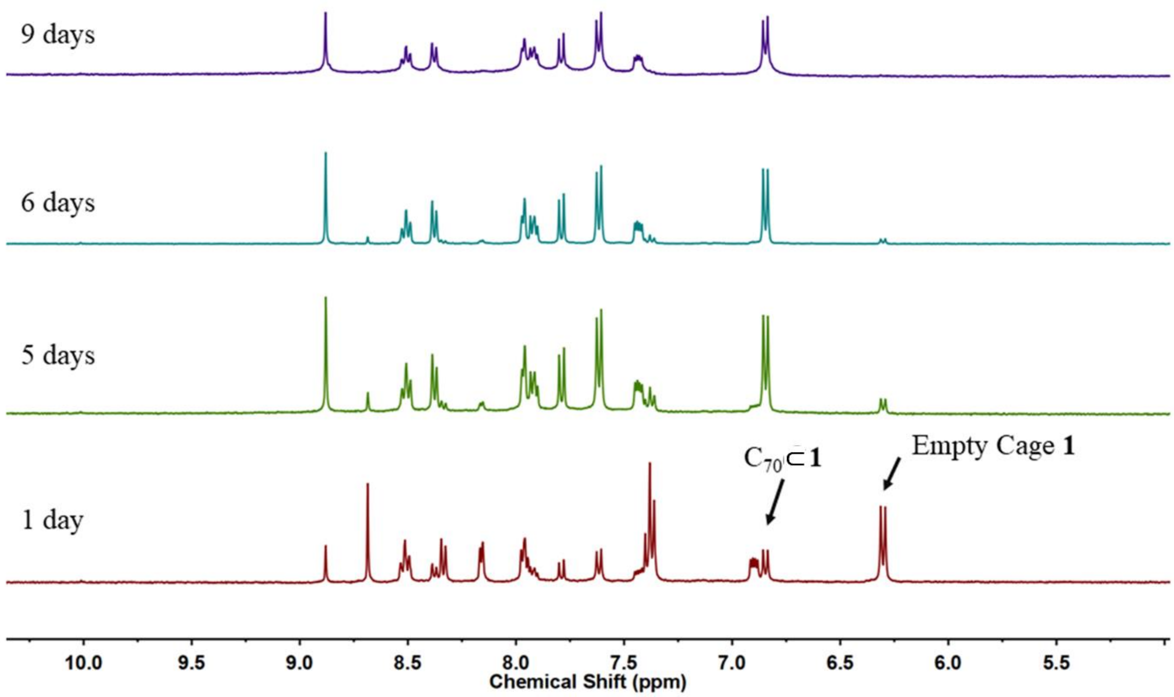

Supplementary Figure 28. Time-dependent ^{1}H NMR spectra of the reaction mixture of Cage **1** with C_{70}. The reaction was kept at 70°C except during NMR measurement.

2.5 Host-guest chemistry of 1 with polyaromatic hydrocarbons

2 mg of cage **1** was dissolved in 0.4 mL CD_3CN, and then different polyaromatic hydrocarbons (5-10 equivalents) were added, respectively. The solutions were kept at 70°C overnight. The samples were cooled to room temperature for 1 hour before NMR determination. For corannulene and pyrene, 50 equivalents of the guests were added to get better NMR spectra for characterisation. Samples with 5-10 equivalents corannulene or pyrene also gave clear evidence for the formation of the host-guest complexes as distinguished from the empty cage.

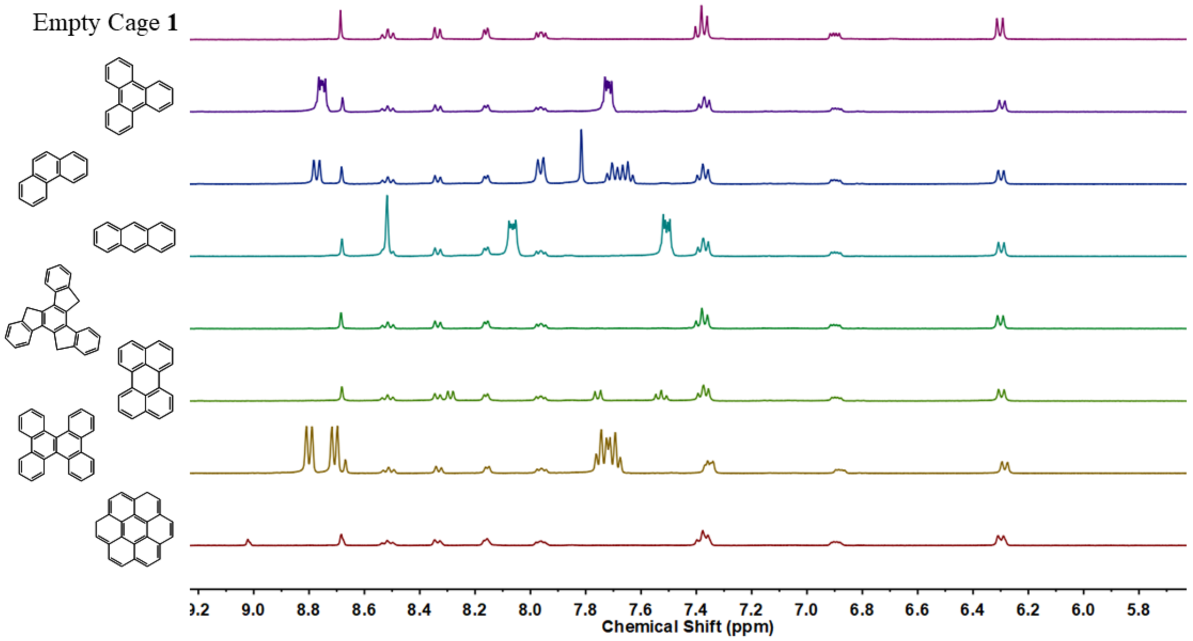

Supplementary Figure 29. ^{1}H NMR spectra of cage **1** and its mixture with different polyaromatic hydrocarbons (5-10 equivalents), respectively, in CD_3CN (400 MHz, 298 K). No obvious changes in the host **1** signals were observed, indicating no interaction with these prospective guests.

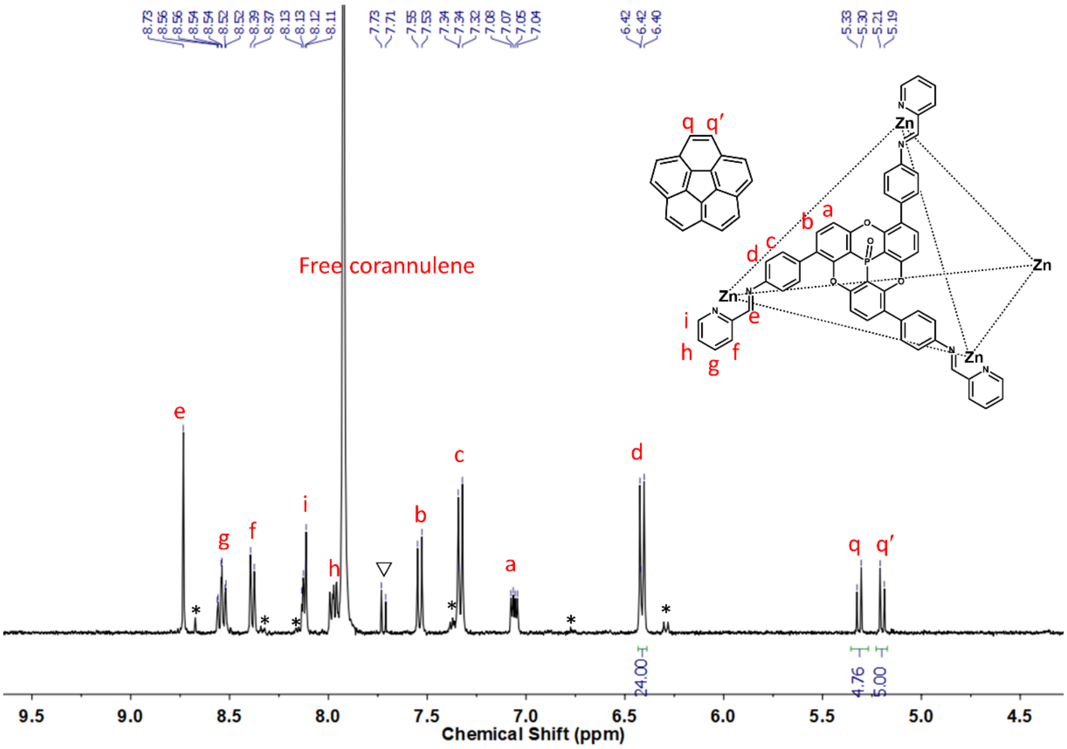

Supplementary Figure 30. ^{1}H NMR spectrum of cage **1** mixed with excess corannulene (about 50 equivalents) in CD_3CN (400 MHz, 298K). The asterisks (*) represent empty **1**. ▽ represents the ^{13}C

satellite signals of the corannulene peak which are also present in the spectrum of free corannulene, see the following figure.

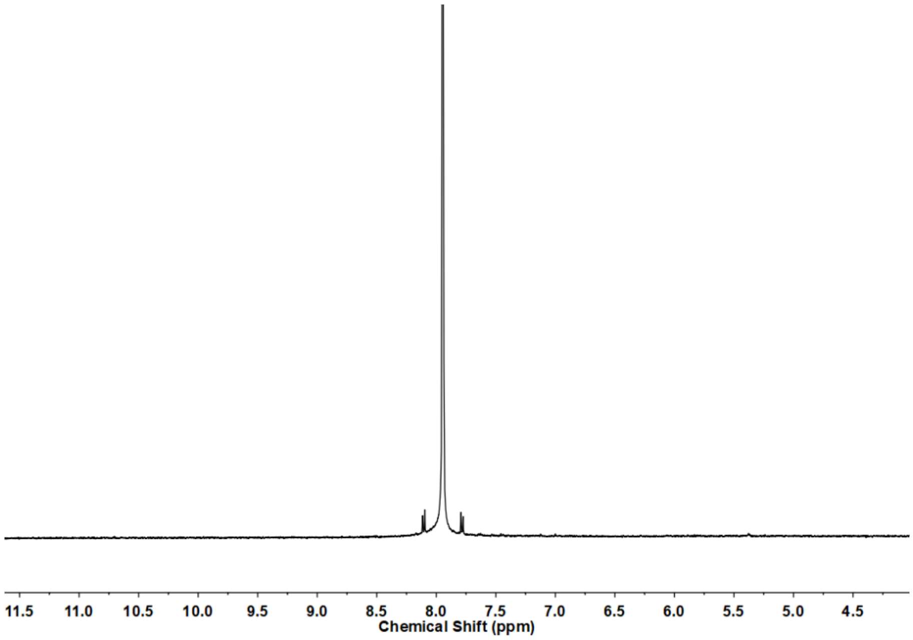

Supplementary Figure 31. ^{1}H NMR spectrum of corannulene in CD_3CN (500 MHz, 298K).

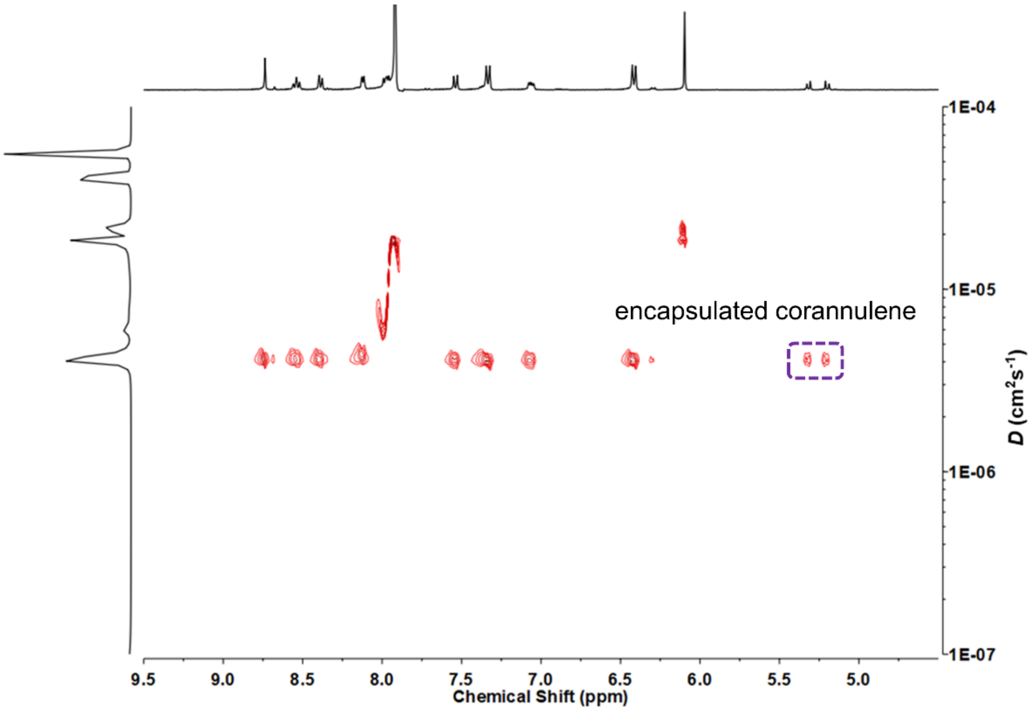

Supplementary Figure 32. ^{1}H DOSY spectrum of cage **1** mixed with excess corannulene in CD_3CN (298K). The encapsulated corannulene has the same diffusion coefficient as cage **1**.

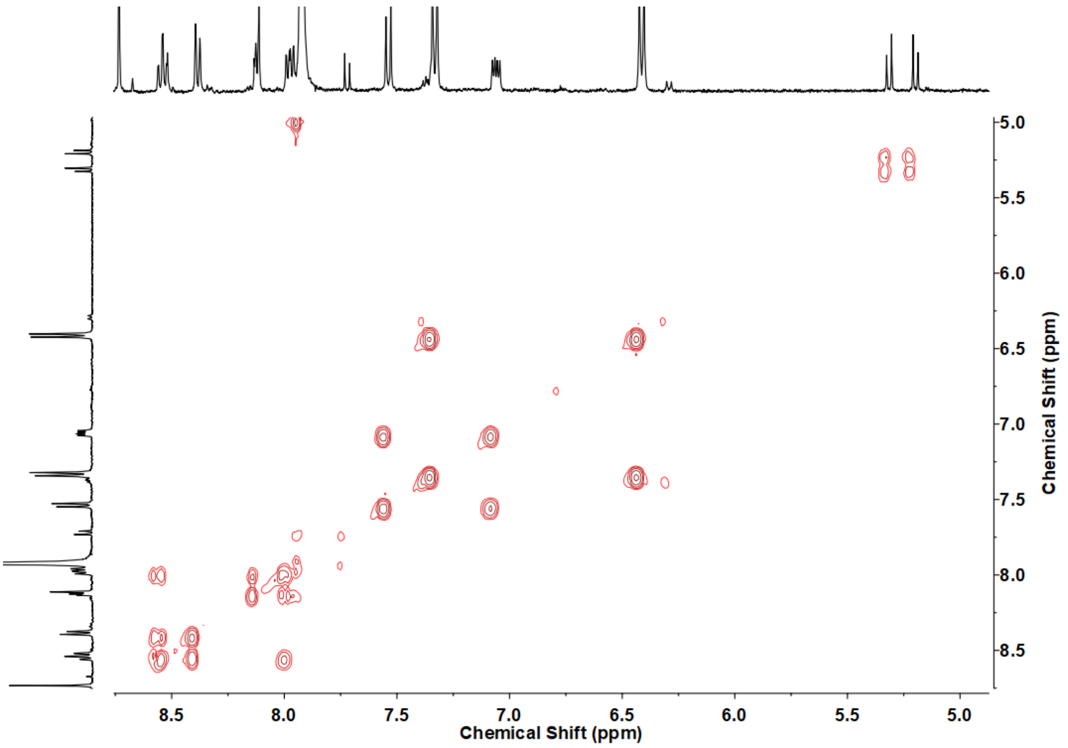

Supplementary Figure 33. ^1H-^1H COSY spectrum of **1** mixed with excess corannulene in CD_3CN (298K).

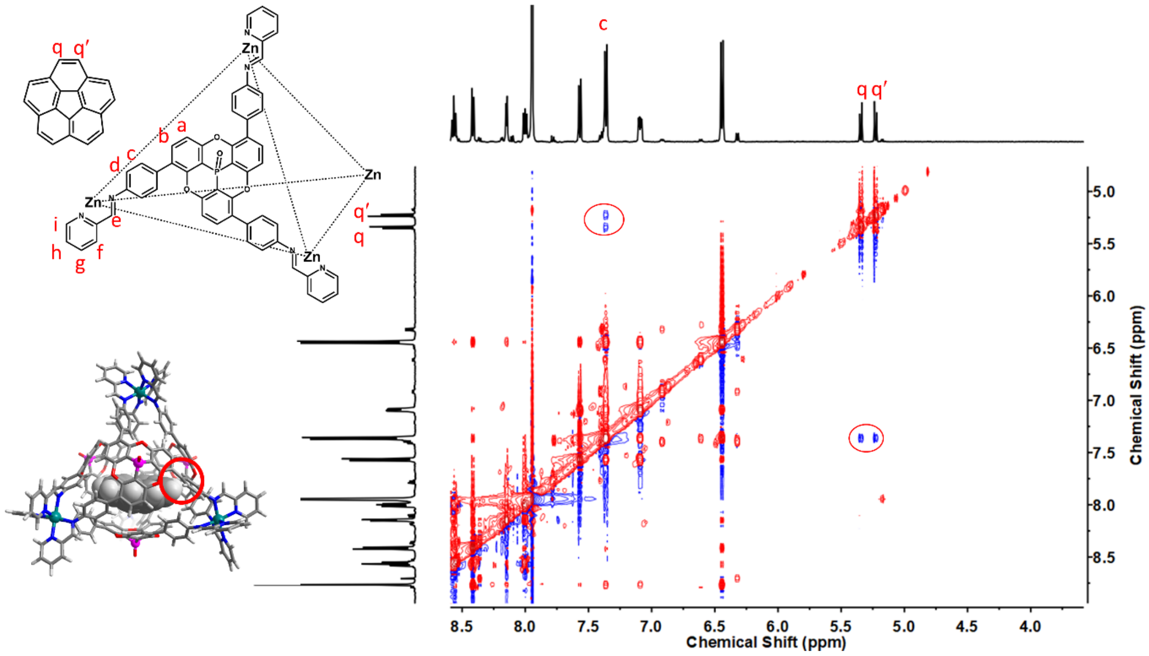

Supplementary Figure 34. ^1H-^1H NOESY spectrum of **1** mixed with excess corannulene in CD_3CN (298K). The red circles highlight the close interactions of encapsulated corannulene with the ligand arms of the cage.

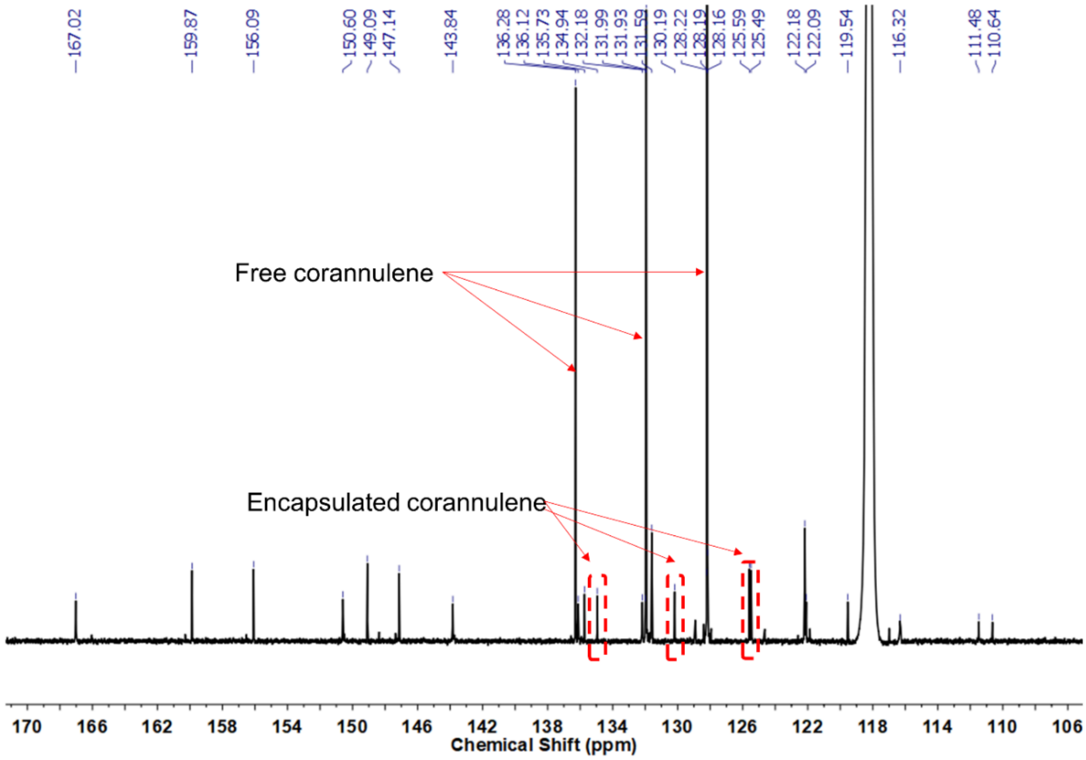

Supplementary Figure 35. ^{13}C NMR spectrum of **1** mixed with excess corannulene in CD_3CN (298K). The free and encapsulated corannulene are labelled. Detailed comparisons with the free cage **1 a**nd hetero-guest complex are shown in **Supplementary Figure 60**.

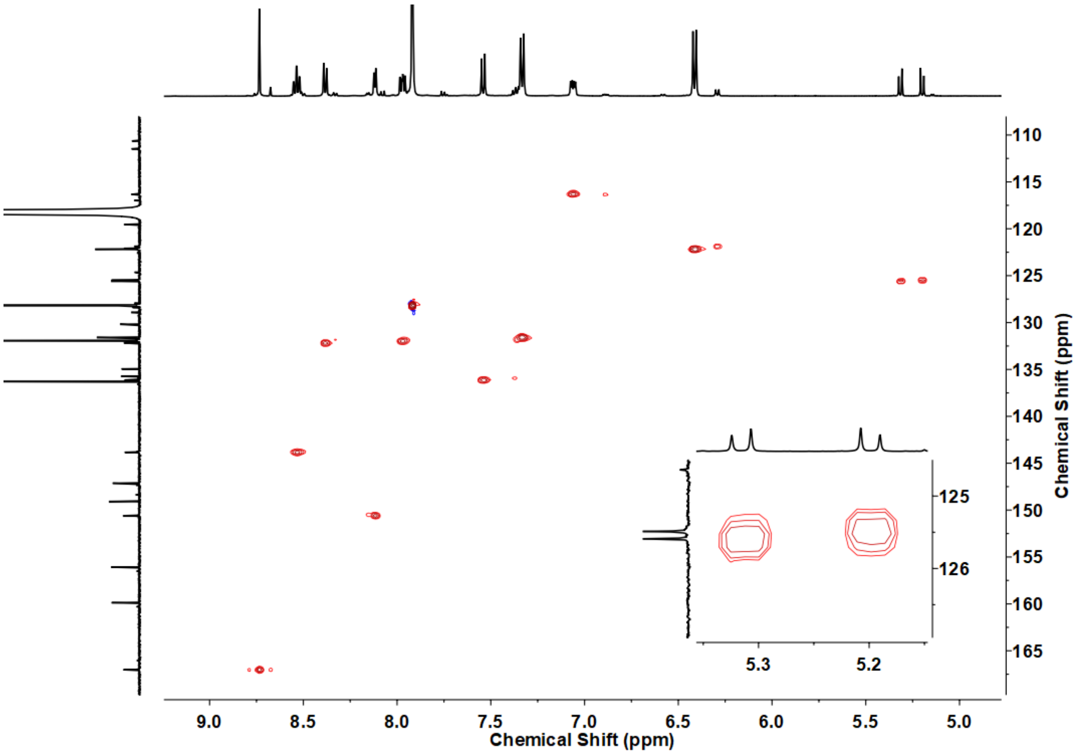

Supplementary Figure 36. ^{1}H-^{13}C HSQC NMR spectrum of **1** mixed with excess corannulene in CD_3CN (298K). Inset: expansion of the signals for the encapsulated corannulene.

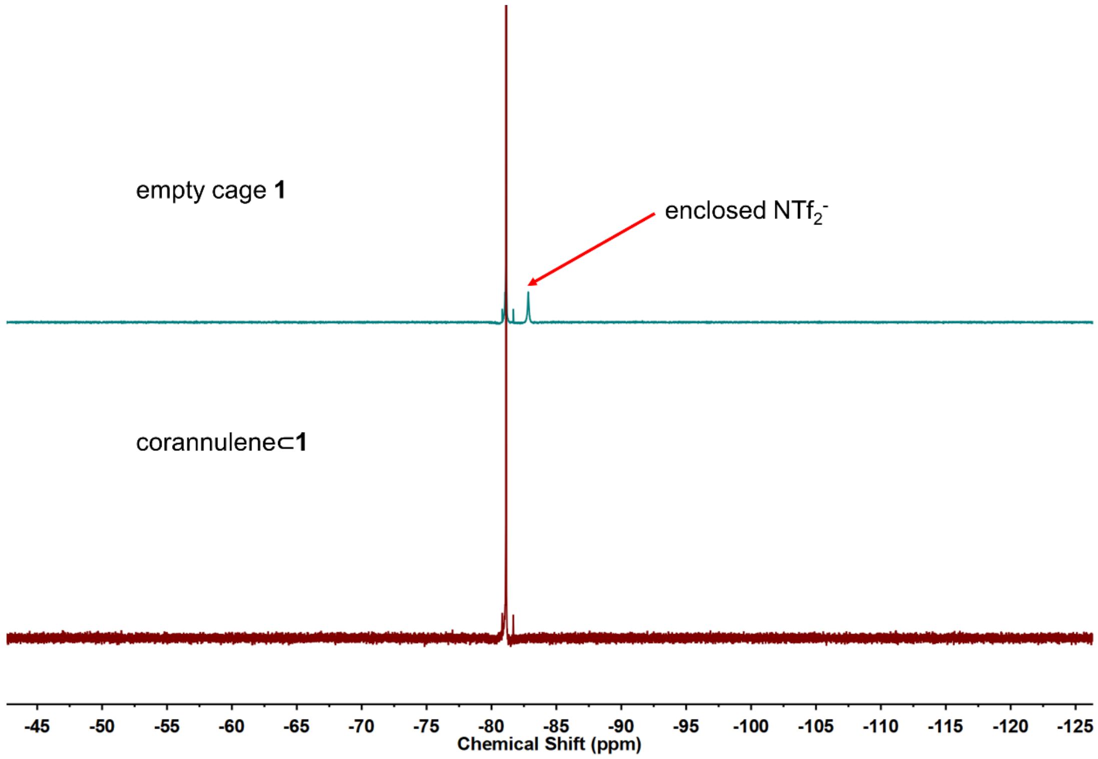

Supplementary Figure 37. Comparison of ^{19}F NMR spectra of **1** (top) and corannulene⊂**1** (bottom) in CD_3CN (298K).

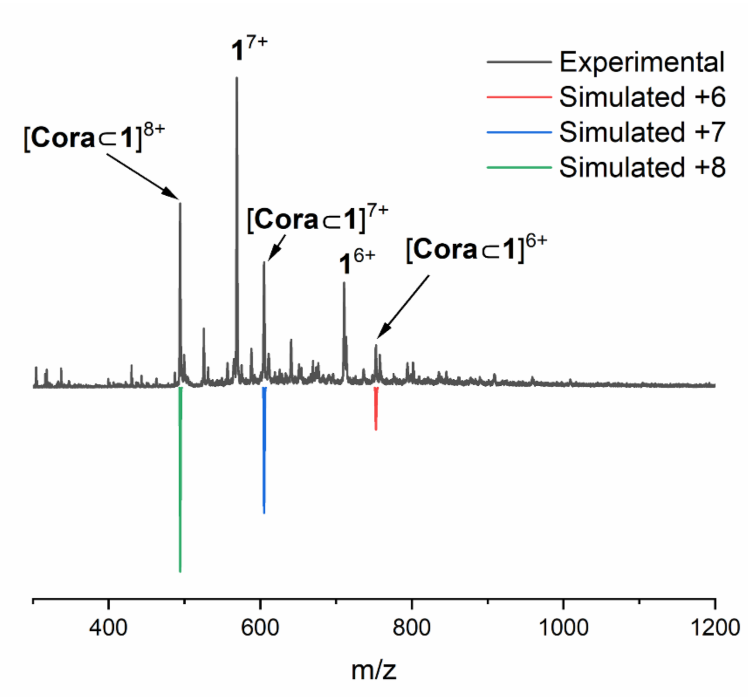

Supplementary Figure 38. Low-resolution ESI-MS of corannulene⊂**1**. The coloured peaks are simulated isotopic ion peaks of corannulene⊂**1** with loss of corresponding numbers of counter ions. **Cora** stands for corannulene.

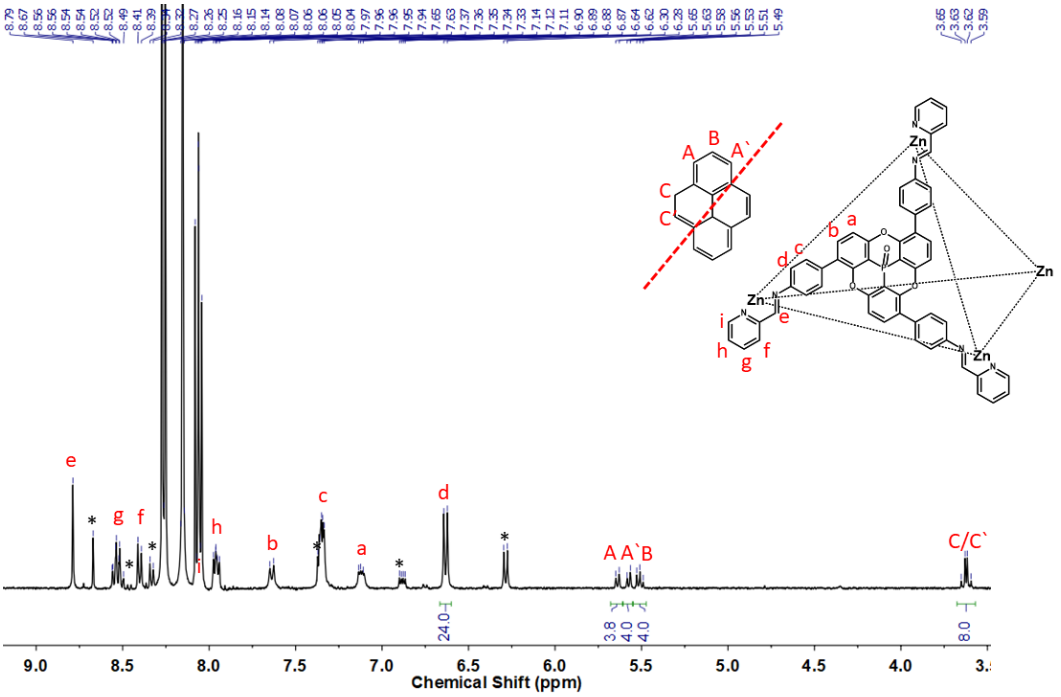

Supplementary Figure 39. ^{1}H NMR spectrum of cage **1** mixed with excess pyrene (about 50 equivalents) in CD_3CN (400 MHz, 298K). The asterisks (*) represent empty **1**. According to the integration, there are two guests inside one host.

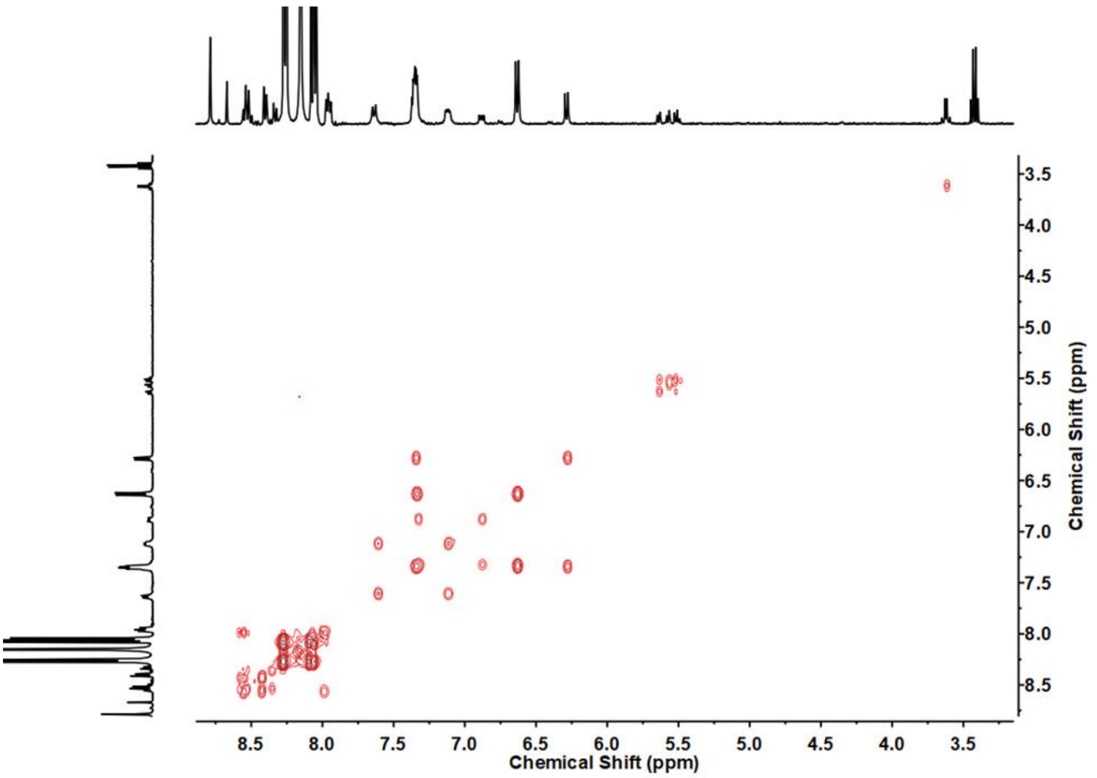

Supplementary Figure 40. ^{1}H-^{1}H COSY spectrum of **1** mixed with excess pyrene in CD_3CN (298K).

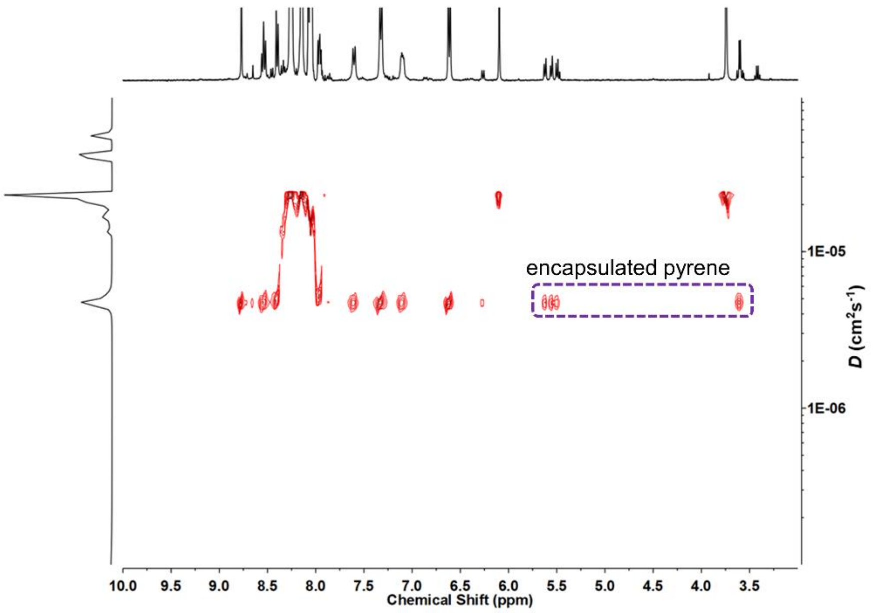

Supplementary Figure 41. ^{1}H DOSY spectrum of cage **1** mixed with excess pyrene in CD_3CN (298K). The encapsulated pyrene signals have the same diffusion coefficient as cage **1**.

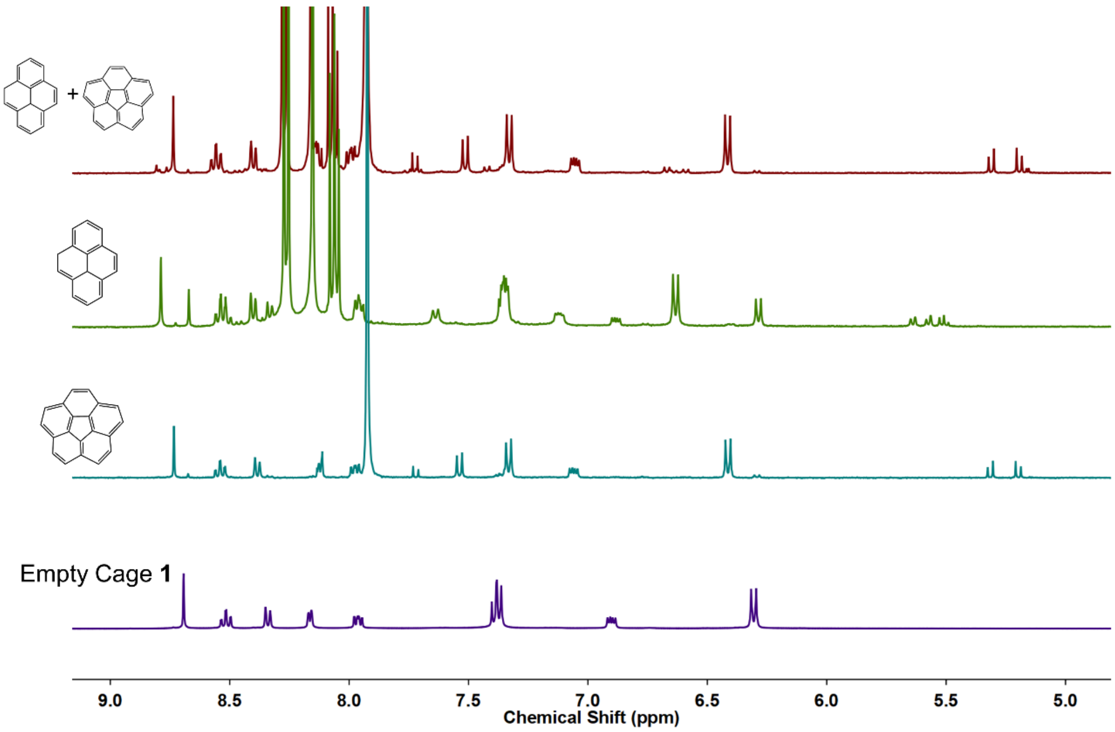

Supplementary Figure 42. The competitive binding experiment conducted with a mixture of the same amount of corannulene and pyrene with Cage **1** in CD_3CN solution (400 MHz, 298 K). The spectra show that when both corannulene and pyrene are present together in the solution, most of the host-guest complexes will be corannulene⊂**1**, indicating **1** has higher affinity for corannulene.

2.6 Host-guest chemistry of 1 with corannulene and cycloalkanes as second guests

A 2 mg cage **1** sample was dissolved in 0.4 mL CD_3CN, and then about 50 equivalents corannulene and 200 equivalents of the cycloalkane were added. The solution was kept at 70°C overnight. The sample was cooled to room temperature for 1 hour before NMR determination.

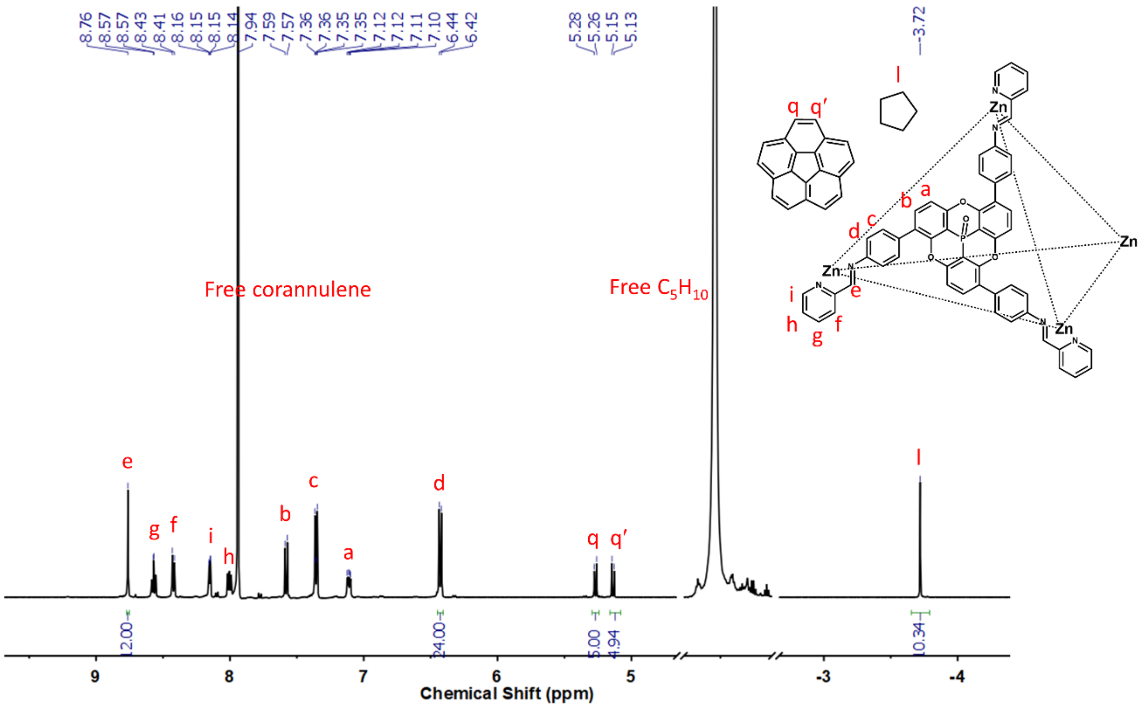

Supplementary Figure 43. ^{1}H NMR spectrum of cage **1** mixed with excess corannulene (about 50 equivalents) and excess cyclopentane (about 200 equivalents) in CD_3CN (400 MHz, 298K).

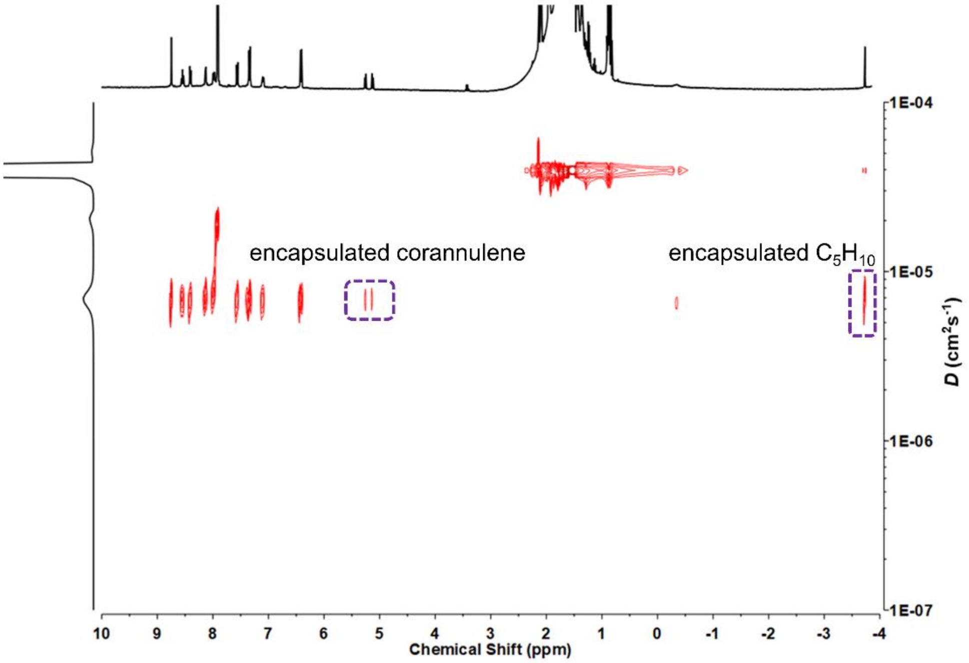

Supplementary Figure 44. ^{1}H DOSY spectrum of cage **1** mixed with excess corannulene and cyclopentane (C_5H_{10}) in CD_3CN (298K). The encapsulated corannulene and cyclopentane signals have the same diffusion coefficient as cage **1**.

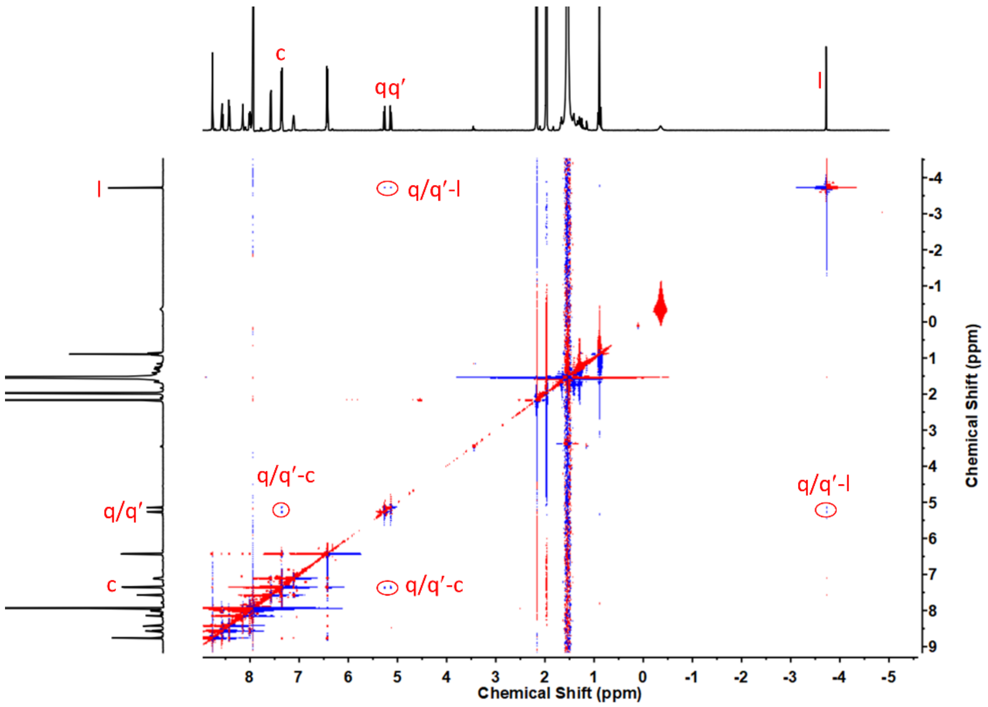

Supplementary Figure 45. ^{1}H-^{1}H NOESY spectrum of **1** mixed with excess corannulene and cyclopentane in CD_3CN (298K). The red circles highlight the close interactions of encapsulated corannulene with the ligand arms of the cage and the interaction of encapsulated corannulene with encapsulated cyclopentane.

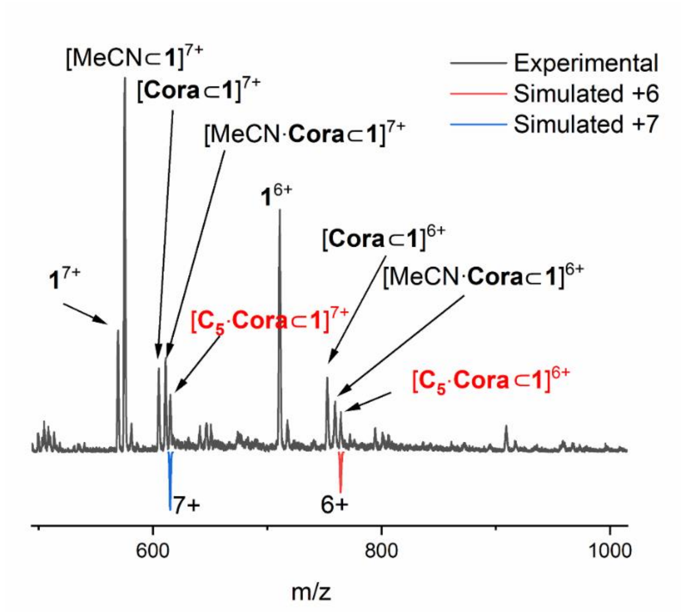

Supplementary Figure 46. Low-resolution ESI-MS of C_5H_{10}•corannulene⊂**1**. The coloured peaks are simulated isotopic ion peaks of C_5H_{10}•corannulene⊂**1** following loss of corresponding numbers of counter ions. **Cora** stands for corannulene, **C**$_5$ stands for cyclopentane.

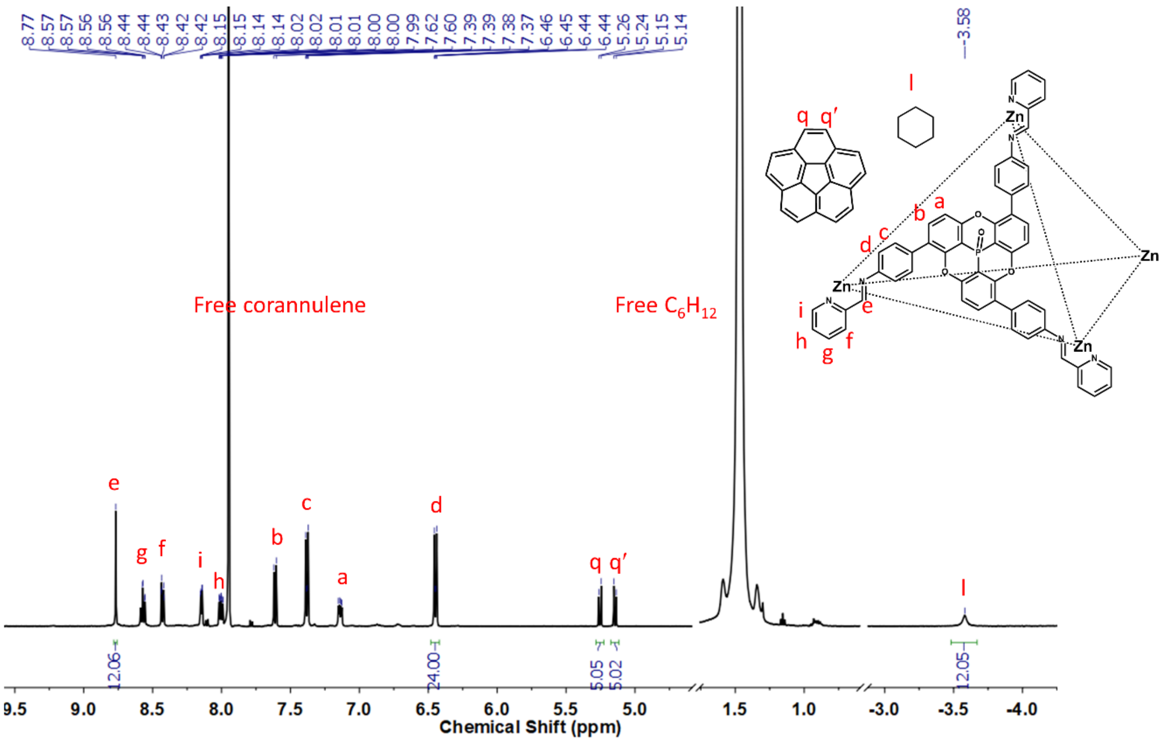

Supplementary Figure 47. ^{1}H NMR spectrum of cage **1** mixed with excess corannulene (about 50 equivalents) and excess cyclohexane (about 200 equivalents) in CD_3CN (400 MHz, 298K).

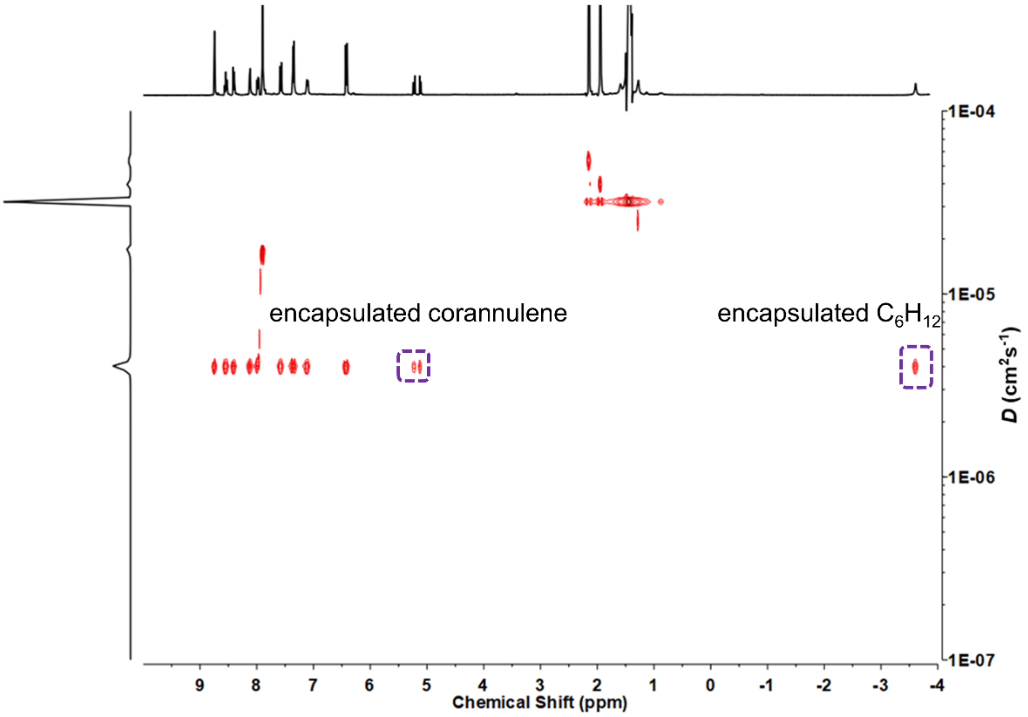

Supplementary Figure 48. ^{1}H DOSY spectrum of cage **1** mixed with excess corannulene and cyclohexane in CD_3CN (298K). The encapsulated corannulene and cyclohexane have the same diffusion coefficient as cage **1**.

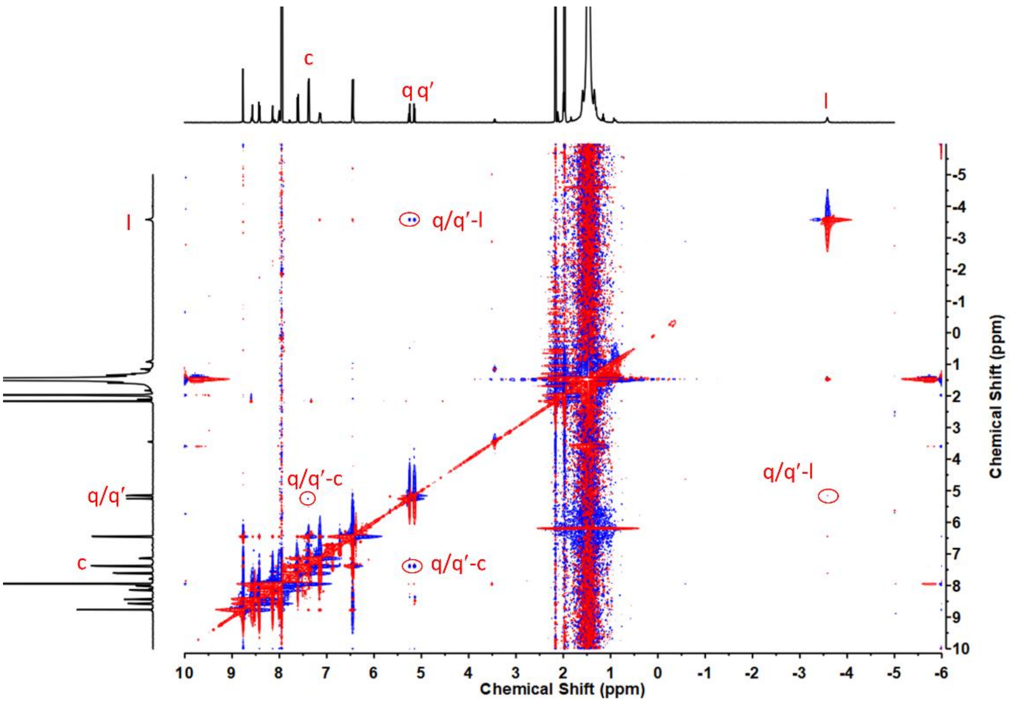

Supplementary Figure 49. ^{1}H-^{1}H NOESY spectrum of **1** mixed with excess corannulene and cyclohexane in CD_3CN (298K). The red circles highlight the close interactions of encapsulated corannulene with the ligand arms of the cage and the interaction of encapsulated corannulene with encapsulated cyclohexane.

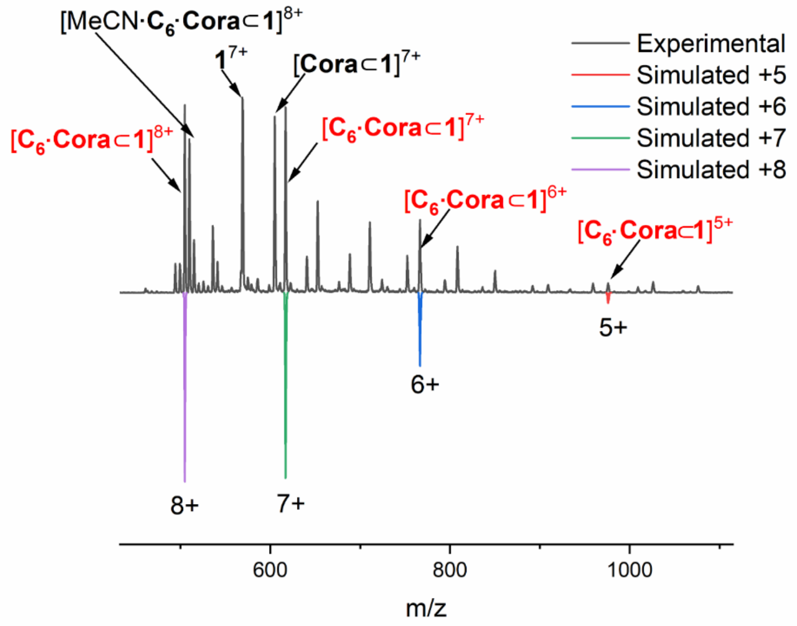

Supplementary Figure 50. Low-resolution ESI-MS of C_6H_{12}•corannulene⊂**1**. The coloured peaks are simulated isotopic ion peaks of C_6H_{12}•corannulene⊂**1** following loss of corresponding numbers of counter ions. **Cora** stands for corannulene, **C$_6$** stands for cyclohexane.

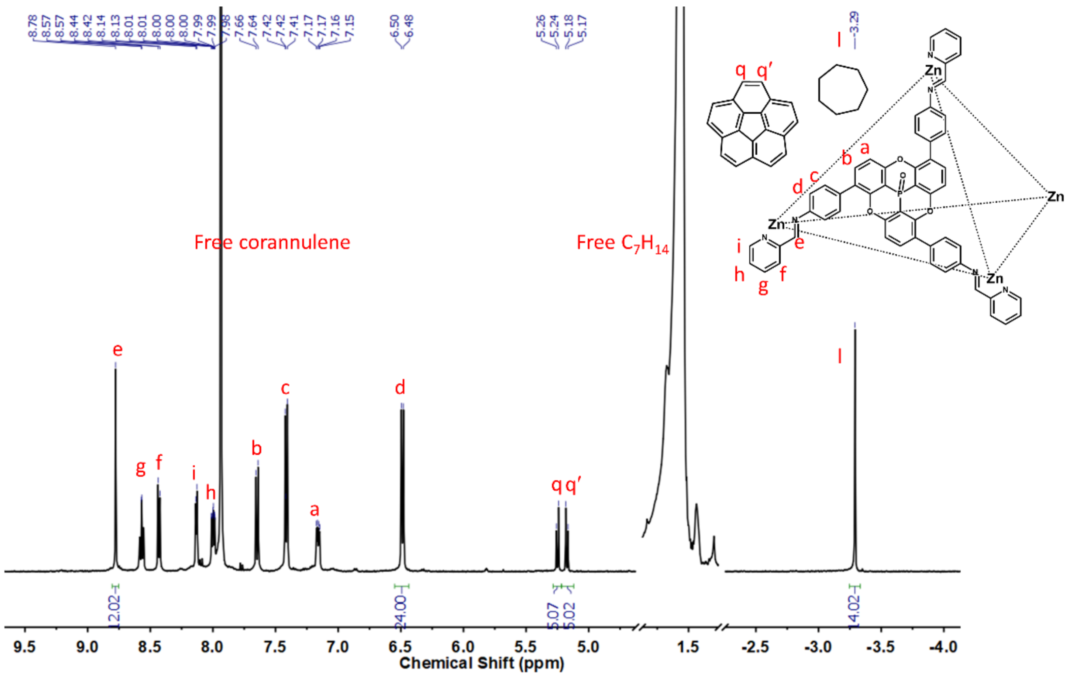

Supplementary Figure 51. ^{1}H NMR spectrum of cage **1** mixed with excess corannulene (about 50 equivalents) and excess cycloheptane (about 200 equivalents) in CD_3CN (400 MHz, 298K).

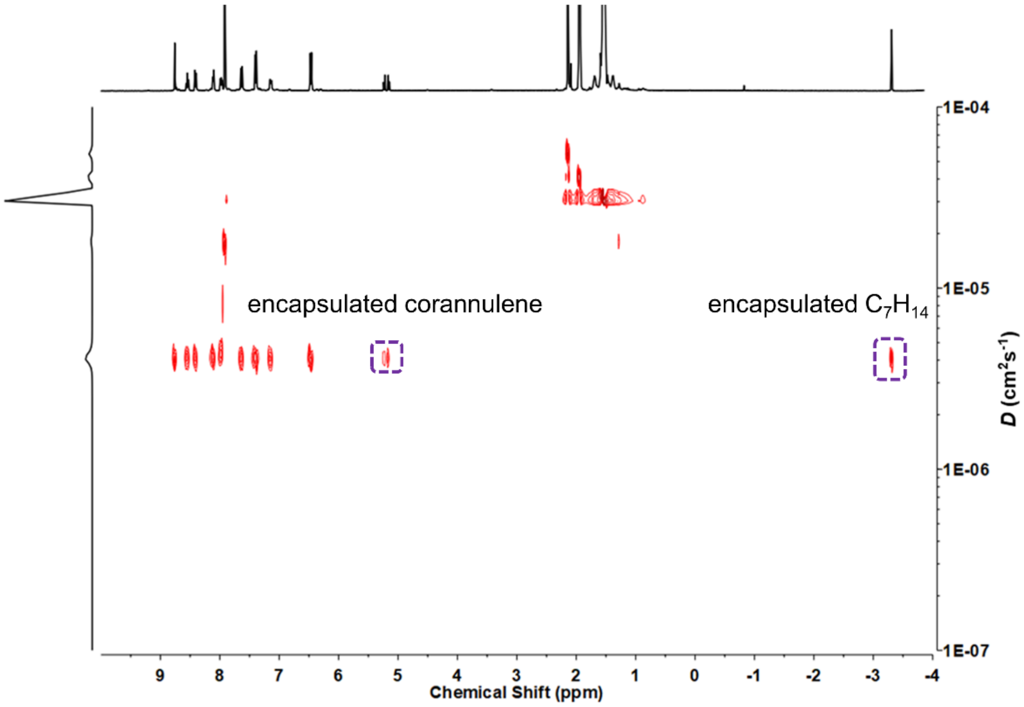

Supplementary Figure 52. ^{1}H DOSY spectrum of cage **1** mixed with excess corannulene and cycloheptane in CD_3CN (298K). The encapsulated corannulene and cycloheptane have the same diffusion coefficient as cage **1**.

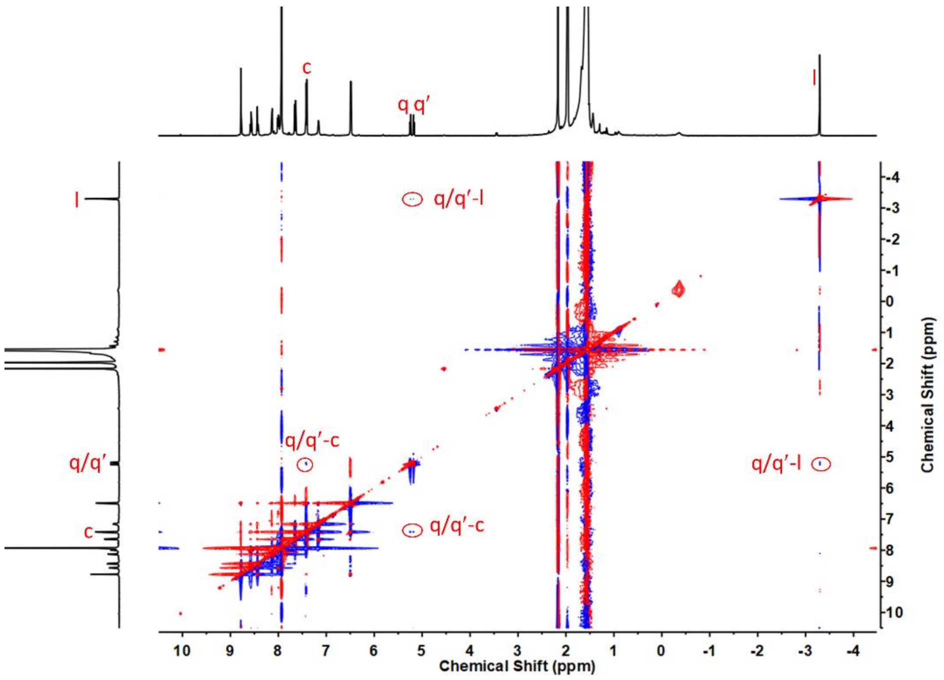

Supplementary Figure 53. ^{1}H-^{1}H NOESY spectrum of **1** mixed with excess corannulene and cycloheptane in CD_3CN (298K). The red circles highlight the close interactions of encapsulated corannulene with the ligand arms of the cage and the interaction of encapsulated corannulene with encapsulated cycloheptane.

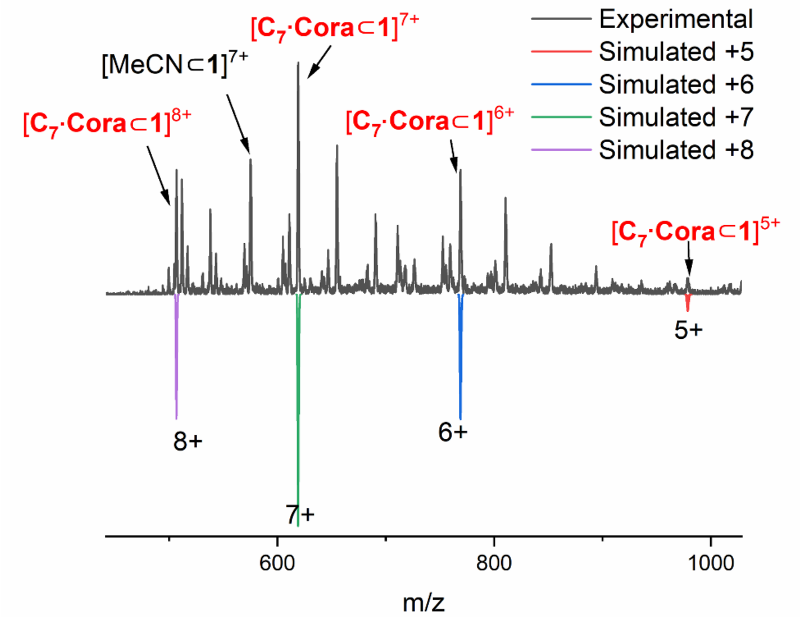

Supplementary Figure 54. Low-resolution ESI-MS spectrum of C_7H_{14}•corannulene⊂**1**. The coloured peaks are simulated isotopic ion peaks of C_7H_{14}•corannulene⊂**1** following loss of corresponding numbers of counter ions. **Cora** stands for corannulene, **C_7** stands for cycloheptane.

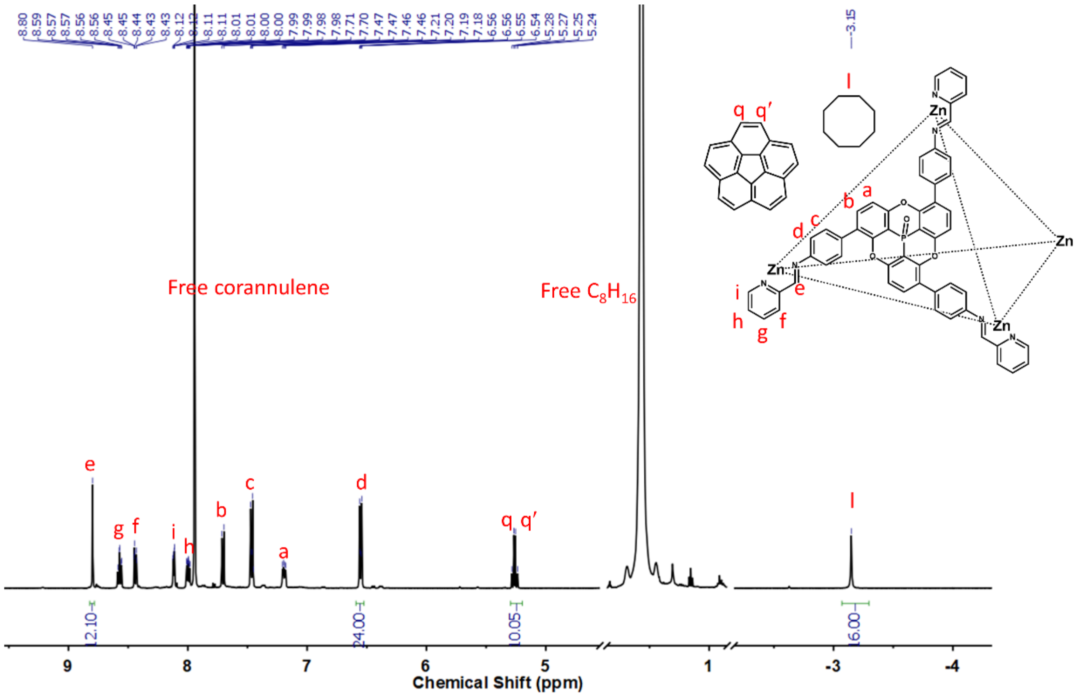

Supplementary Figure 55. ^{1}H NMR spectrum of cage **1** mixed with excess corannulene (about 50 equivalents) and excessive cyclooctane (C_8, about 200 equivalents) in CD_3CN (400 MHz, 298K).

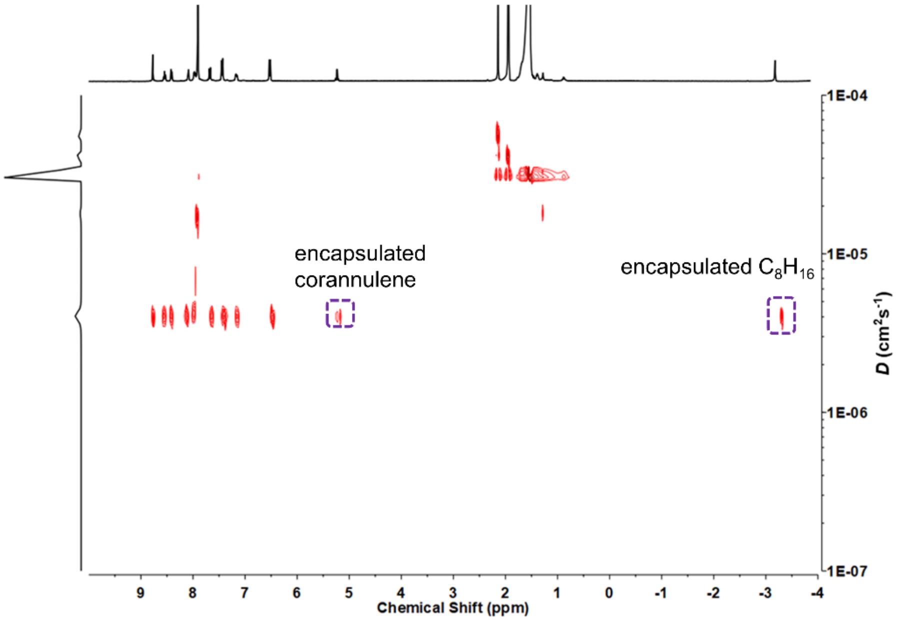

Supplementary Figure 56. ^{1}H DOSY spectrum of cage **1** mixed with excess corannulene and cyclooctane in CD_3CN (298K). The encapsulated corannulene and cyclooctane have the same diffusion coefficient as the cage **1**.

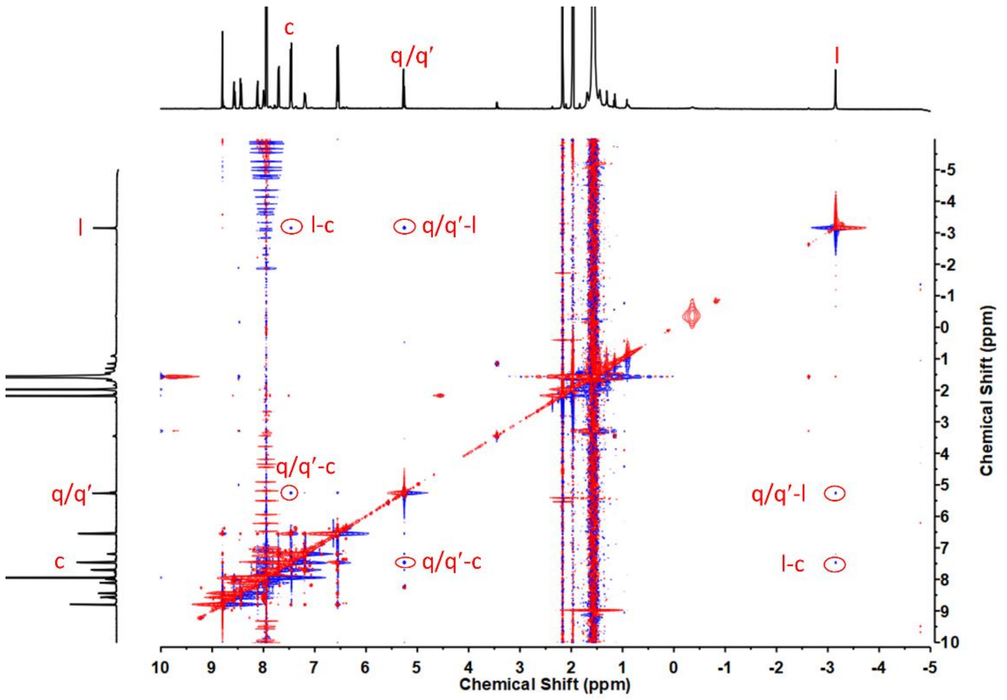

Supplementary Figure 57. ^{1}H-^{1}H NOESY spectrum of **1** mixed with excess corannulene and cyclooctane in CD_3CN (298K). The red circles highlight the close interactions of encapsulated corannulene with the ligand arms of the cage and the interaction of encapsulated corannulene with encapsulated cyclooctane.

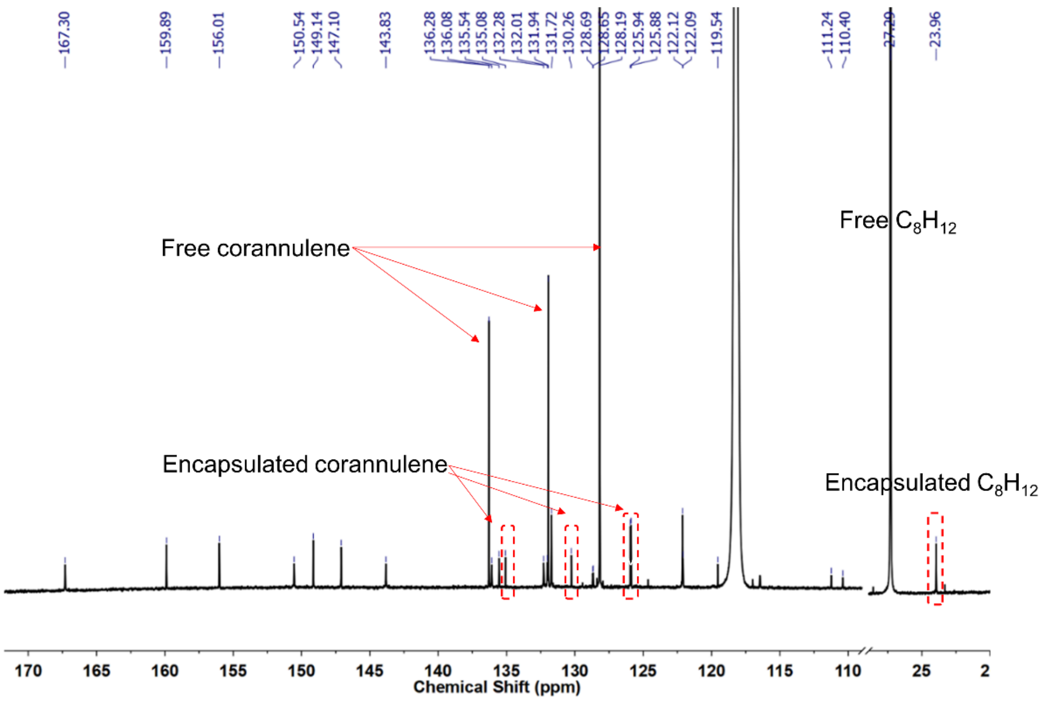

Supplementary Figure 58. ^{13}C NMR spectrum of **1** mixed with excess corannulene and cyclooctane in CD_3CN (298K).

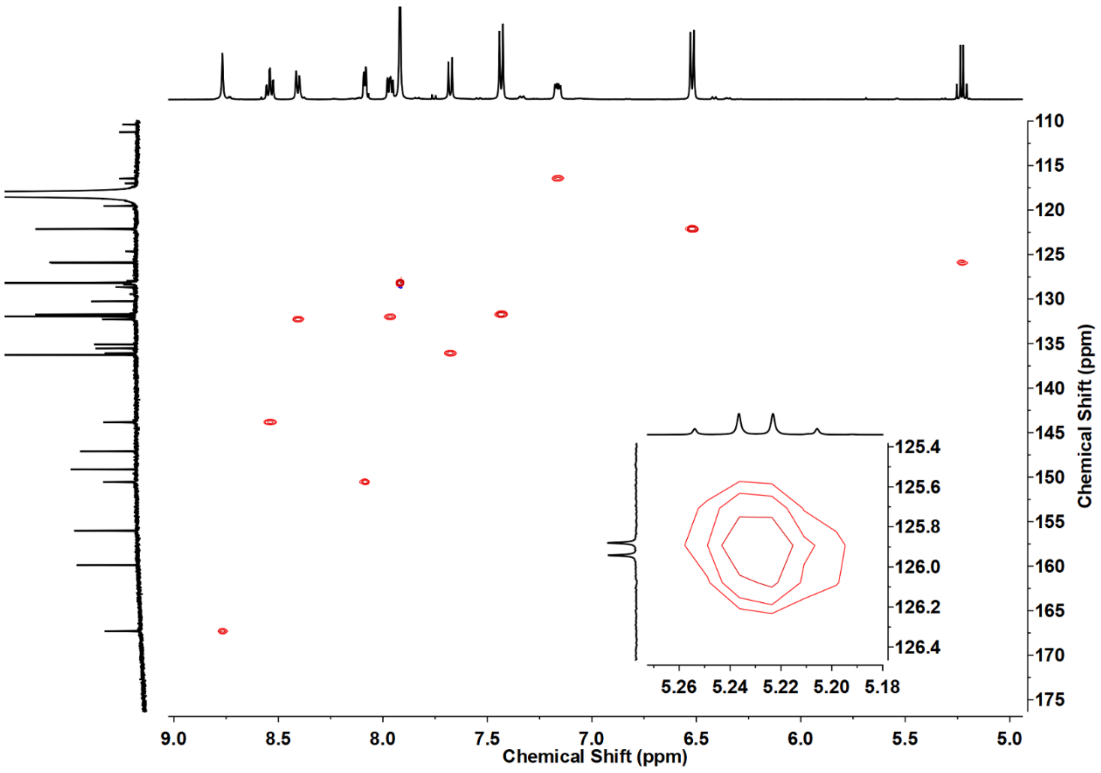

Supplementary Figure 59. ^1H-^{13}C HSQC spectrum of **1** mixed with excess corannulene and cyclooctane in CD_3CN (298K). The inset shows the magnification of the peak for bound corannulene.

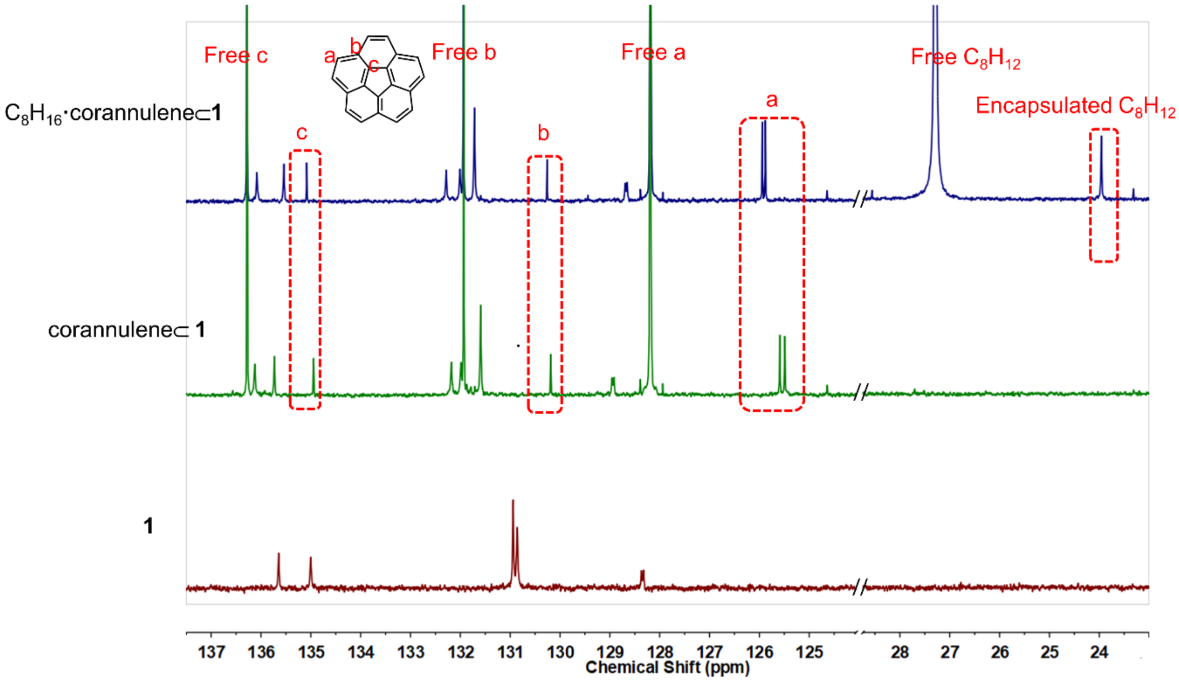

Supplementary Figure 60. Comparison of the ^{13}C NMR spectra of **1**, corannulene⊂**1** and C_8H_{16}•corannulene⊂**1** showing the region with the corannulene peaks and the C_8H_{16} peaks. The red

boxes highlight the encapsulated corannulene and C_8H_{16}. The splitting of the carbons a (C_a) within the chiral environment is in line with their corresponding protons.

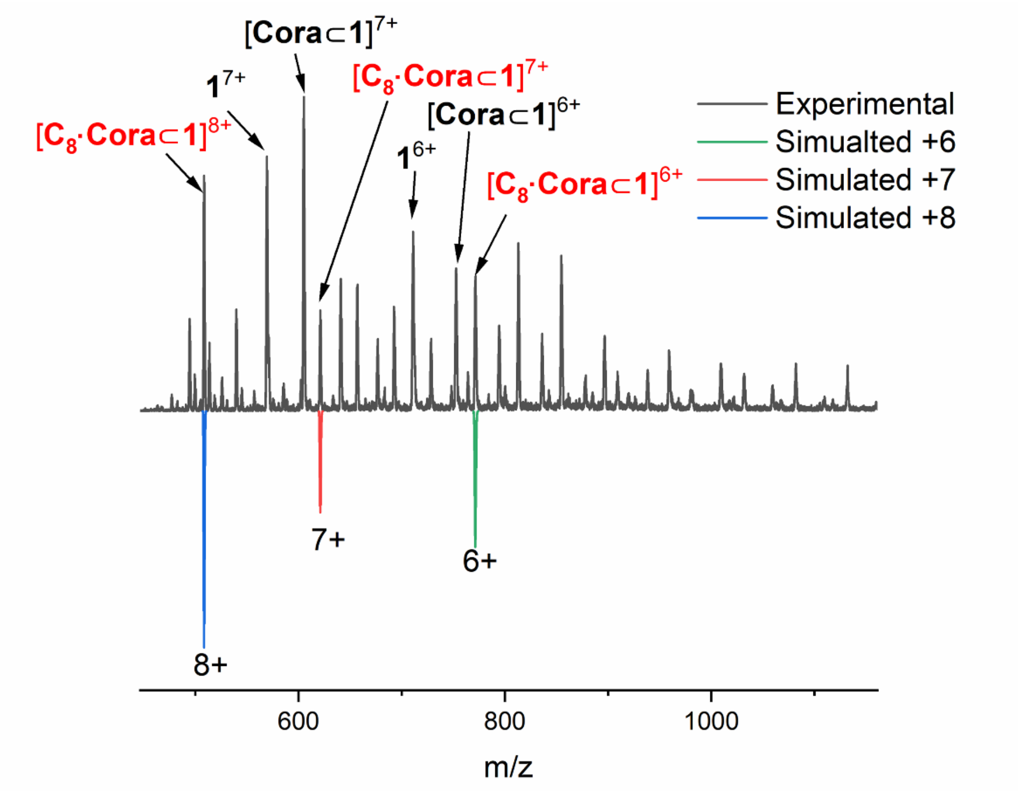

Supplementary Figure 61. Low-resolution ESI-MS of C_8H_{16}•corannulene⊂**1**. The coloured peaks are simulated isotopic ion peaks of C_8H_{16}•corannulene⊂**1** following loss of corresponding numbers of counter ions. **Cora** stands for corannulene, **C_8** stands for cyclooctane.

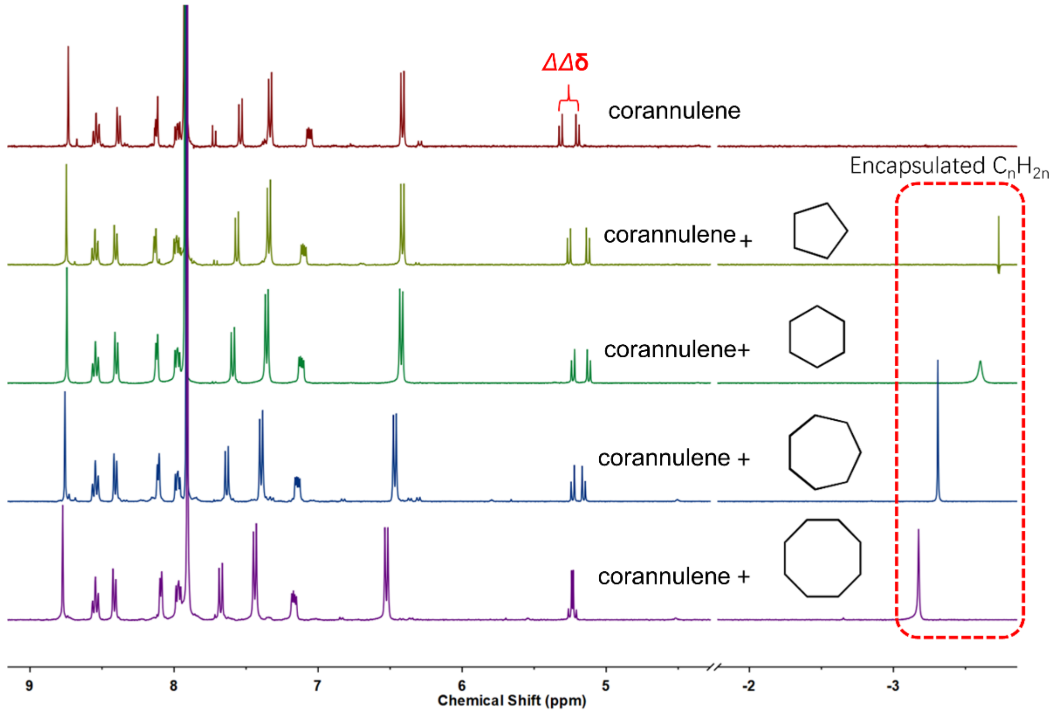

Supplementary Figure 62. Comparison of the ^{1}H NMR spectra (CD_3CN, 400 MHz, 298 K) of corannulene⊂**1**, C_5H_{10}•corannulene⊂**1**, C_6H_{12}•corannulene⊂**1**, C_7H_{14}•corannulene⊂**1** and C_8H_{16}•corannulene⊂**1**.

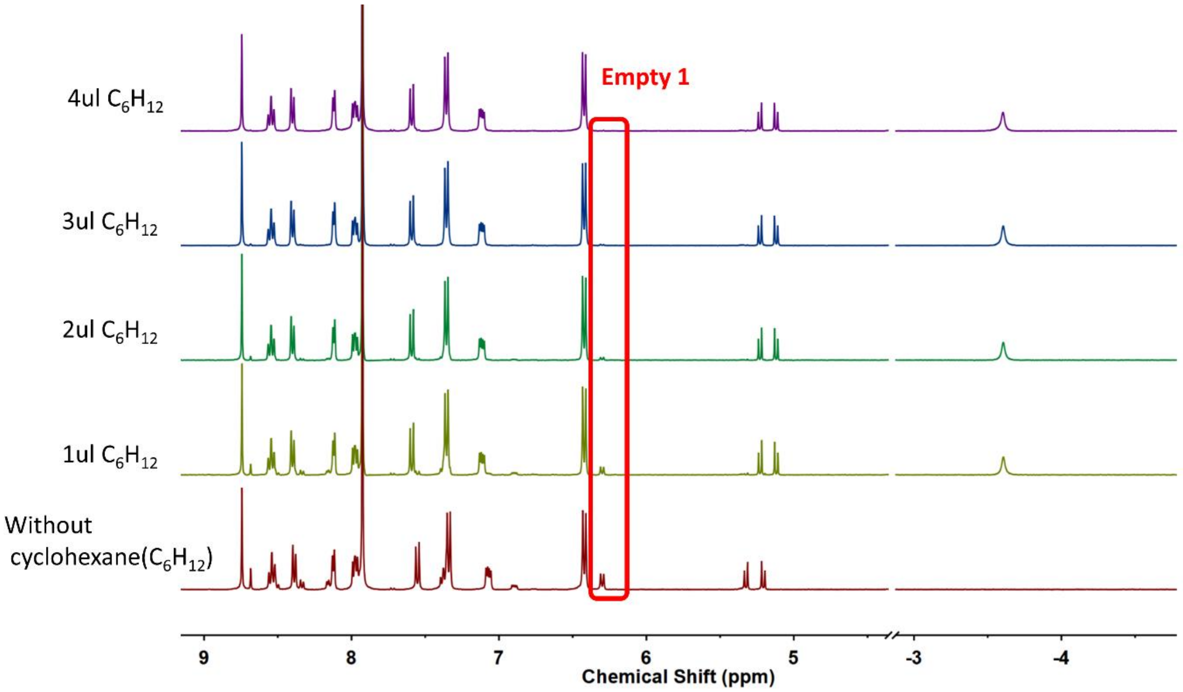

Supplementary Figure 63. ^{1}H NMR spectra (CD_3CN, 400 MHz, 298 K) of sequential addition of cyclohexane into a solution of cage **1** mixing with 5.5 equivalents of corannulene. More cyclohexane

promotes the encapsulation of corannulene. Without cyclohexane, 18% of the cages are empty. After the addition of 4 μL cyclohexane (ca. 300 equivalents), no empty cage is observed.

2.7 Host-guest chemistry of 1 with corannulene and chiral 3-methyl-2-butanol as the second guest

1.5 mg (0.00025 mmol) cage **1** sample was dissolved in 0.4 mL CD_3CN, and then about 0.3 mg (0.0012 mmol, 4.8 equivalents) corannulene and 5 μl (ca. 270 equivalents) *R*-3-methyl-2-butanol were added. The solution was kept at 70°C overnight. The sample was cooled to room temperature for 1 hour before NMR determination.

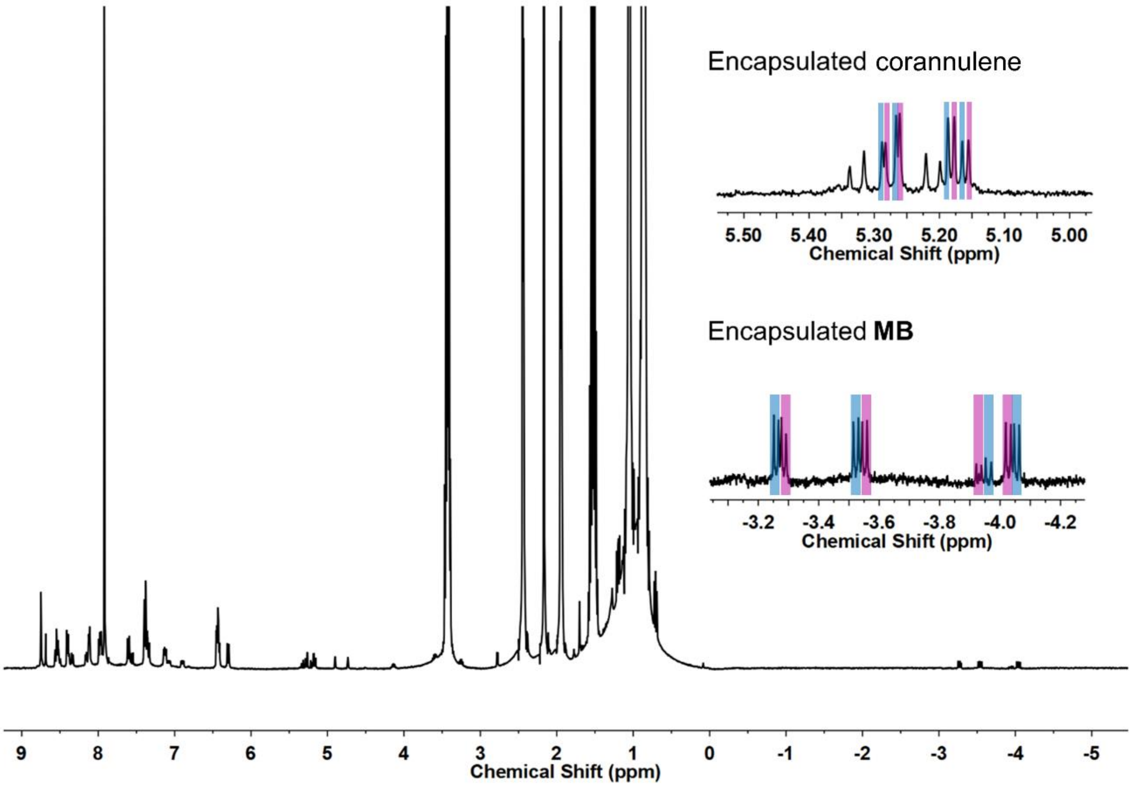

Supplementary Figure 64. ^{1}H NMR spectra following addition of *R*-3-methyl-2-butanol (*R*-**MB**) into a mixture of **1** and corannulene in CD_3CN (400 MHz, 298 K). Two set of peaks for the encapsulated corannulene and **MB** are observed (labelled with different colours).

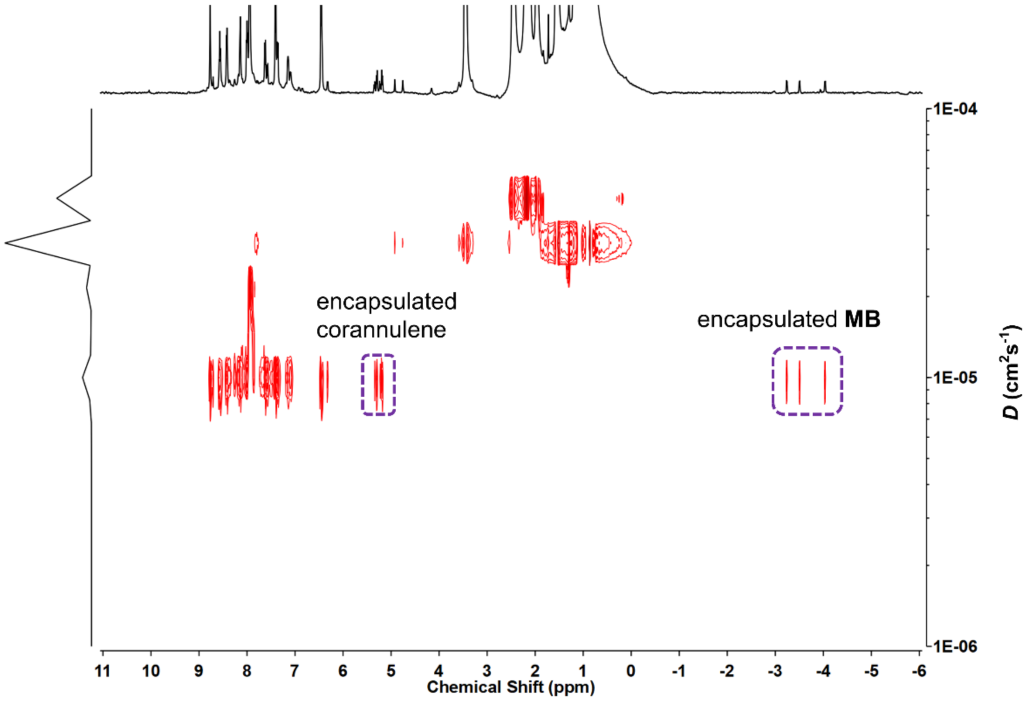

Supplementary Figure 65. ^{1}H DOSY spectrum of cage **1** mixed with excess corannulene and *R*-3-methyl-2-butanol (**MB**) in CD_3CN (298K). The encapsulated corannulene and *R*-3-methyl-2-butanol have the same diffusion coefficient as cage **1**.

3. Determination of the binding Constants *K*a.

3.1 Binding constant for corannulene and pyrene

In general, a 0.53 mM stock solution of cage **1** was combined with different amounts of corannulene or pyrene guest, together with a known amount of trimethoxybenzene as an internal standard. The samples were kept at 70°C for one day. After cooling down to room temperature for 1 hour, they were measured by NMR. The peaks of empty host **1**, guest corannulene/pyrene and the host-guest complexes (corannulene⊂**1** and (pyrene)$_2$⊂**1**) were well separated in the ^{1}H NMR spectra. The peaks used for concentration determination are labelled in the following figures. The concentrations were calibrated by integration relative to the trimethoxybenzene standard.

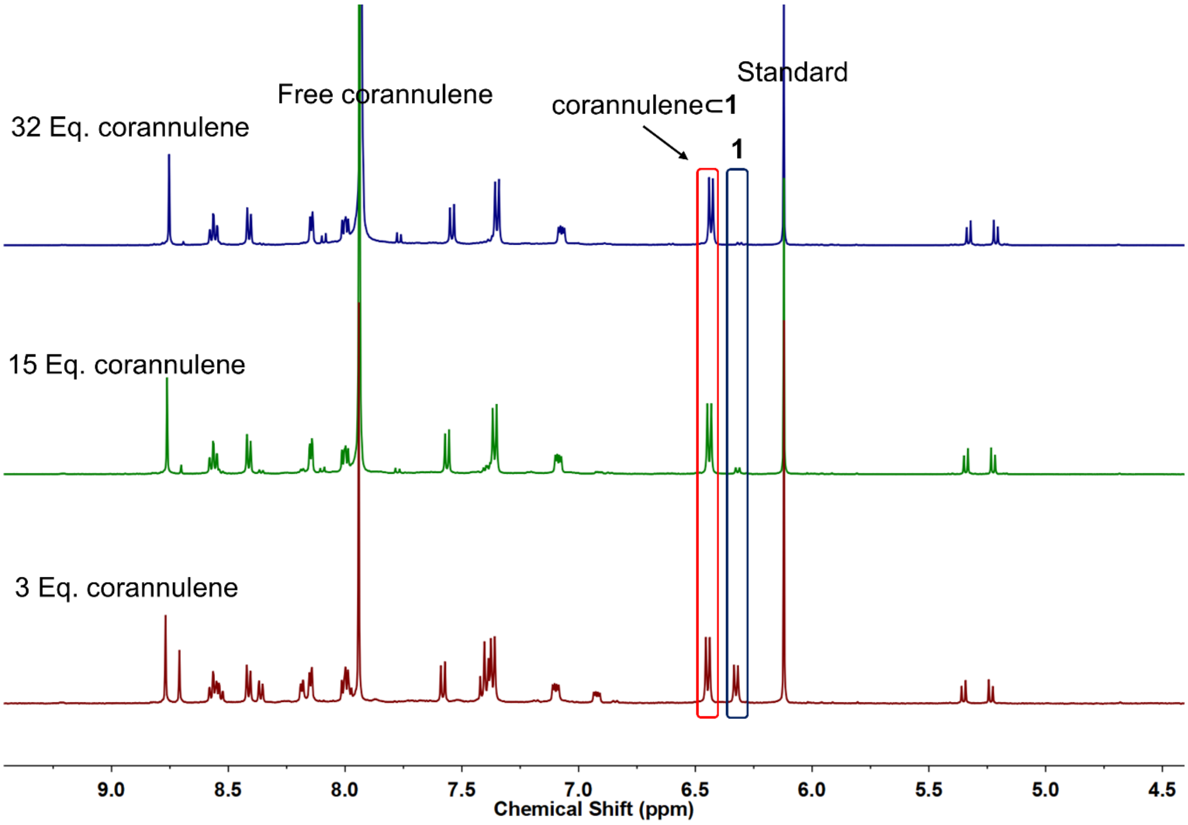

Supplementary Figure 66. ^{1}H NMR spectra of cage **1** (0.53 mM) with different amounts of corannulene guest (CD_3CN, 500 MHz, 298 K). The peaks of free corannulene, empty cage **1**, host-guest complex corannulene⊂**1** and the trimethoxybenzene standard are labelled. The averaged binding constant $K_a = \frac{[HG]}{[H][G]}$ was evaluated to be (1.13±0.12) ×10^3 M^{-1}.

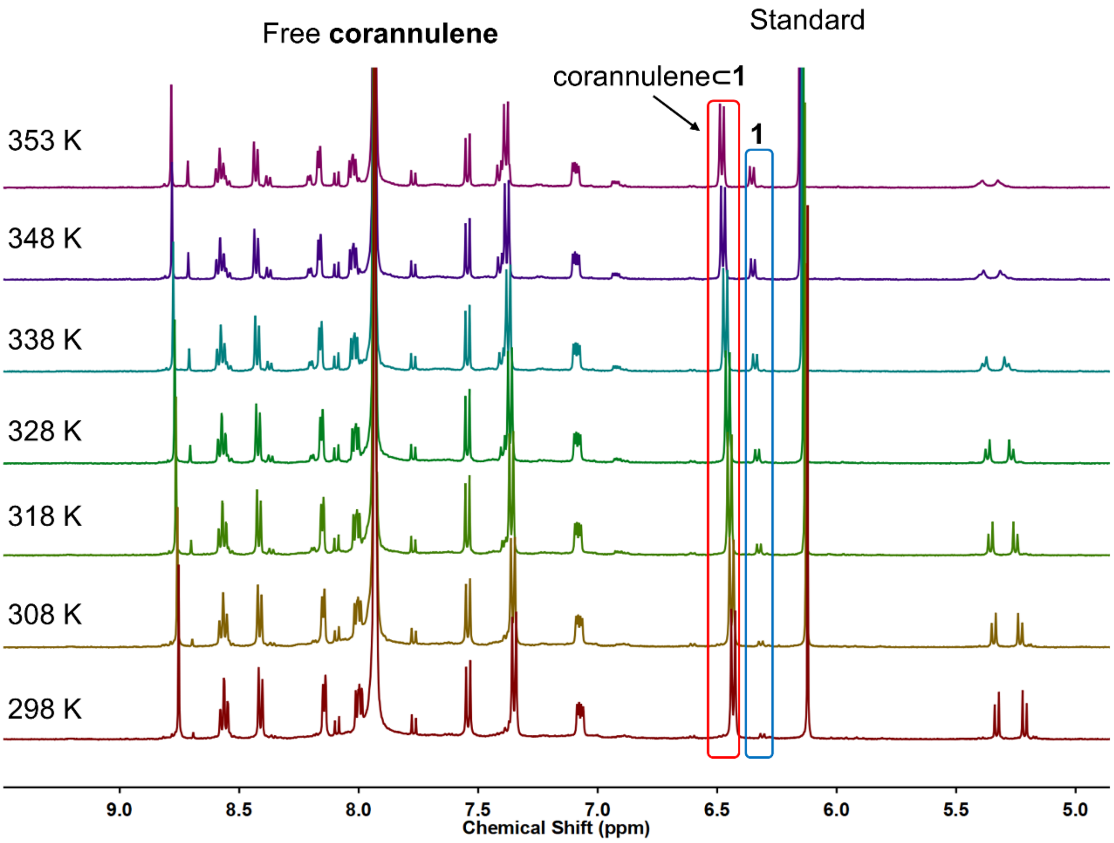

Supplementary Figure 67 Variable temperature ^{1}H NMR spectra of cage **1** (0.53 mM) with corannulene (32 Eq.) guest (CD_3CN, 500 MHz). The peaks of free corannulene, empty cage **1**, host-guest complex corannulene⊂**1** and the trimethoxybenzene standard are labelled.

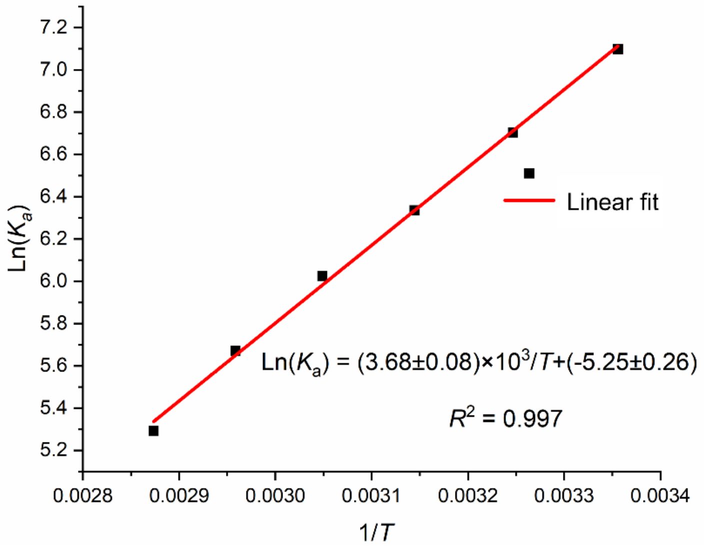

Supplementary Figure 68. Van't Hoff plot for the binding of corannulene by cage **1**. According to the van't Hoff equation $\ln(K_a) = -\Delta H/(RT) + \Delta S/R$, the ΔH and ΔS is determined to be -30.6±0.4 kJ·mol^{-1} and 43.6±1.4 J·K^{-1}·mol^{-1}, respectively.

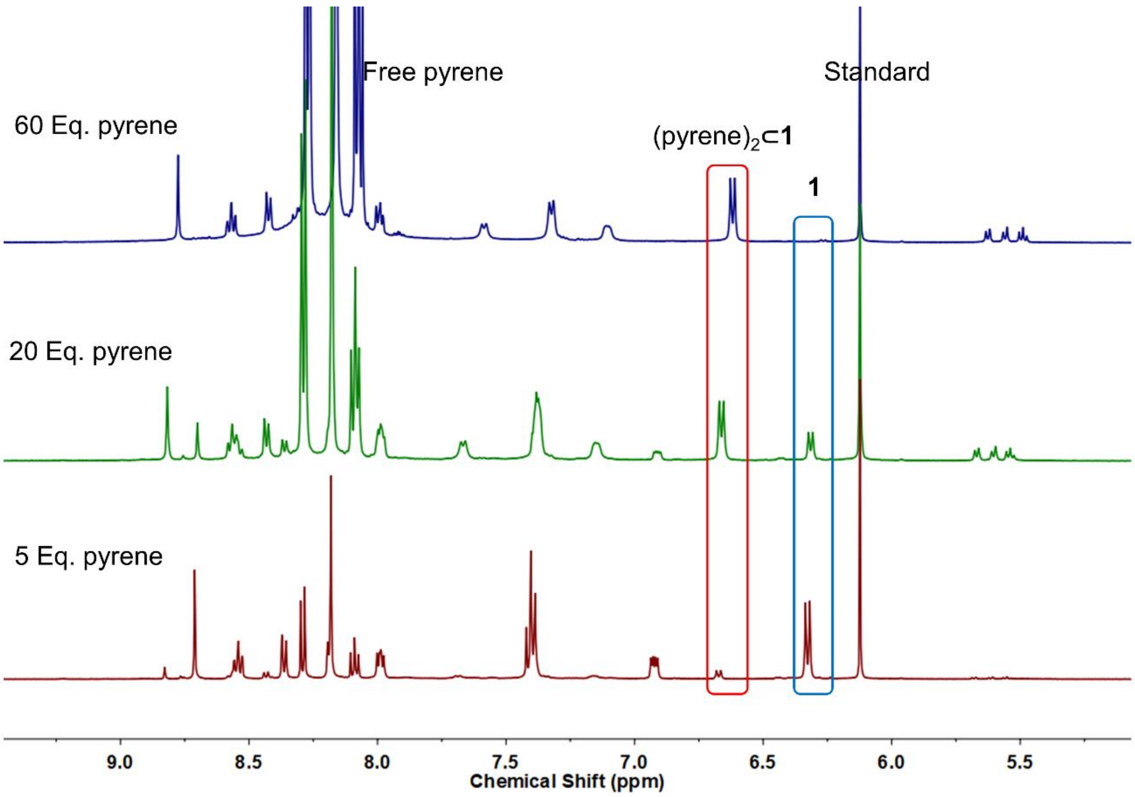

Supplementary Figure 69. ^{1}H NMR spectra of cage **1** (0.53 mM) with different amounts of pyrene guest (CD_3CN, 500 MHz, 298 K). The peaks of free pyrene, empty cage **1**, host-guest complex (pyrene)$_2$⊂**1** and the trimethoxybenzene standard are labelled. According the integration, there are two guests inside one host. Only peaks for the empty host and 2:1 H-G complex were observed with no evidence of a 1:1 H-G complex suggesting the binding of pyrene is cooperative. The average binding constant $K_a = \frac{[HG]}{[H][G]^2}$ was evaluated to be (1.66±0.46)×10^4 M^{-2}.

3.2 Binding constant for hetero-guest systems

In general, a 0.42 mM stock solution of cage **1** was combined with 5 equivalents corannulene guest and different amounts (0.25 - 1.5 μL) of cycloalkanes (C_5H_{10} – C_8H_{16}) guests, together with a known amount of trimethoxybenzene as the internal standard. The samples were kept at 70°C for two days. After cooling down to room temperature for 1 hour, they were measured by NMR. The peaks of empty host **1**, guest corannulene, cycloalkanes (C_5H_{10} – C_8H_{16}) guests and the host-guest complexes (corannulene⊂**1** and C_nH_{2n}•corannulene⊂**1**) are well separated in the ^{1}H NMR spectra. The peaks used for concentration determination are labelled in the following figures and additional notes are provided in the captions of the figures. All the concentrations were calibrated by integration relative to the trimethoxybenzene standard. The binding constants K_a are determined by averaging three samples with different concentrations as shown in Supplementary Figures 70-73. The errors correspond to the estibated standard deviations between samples.

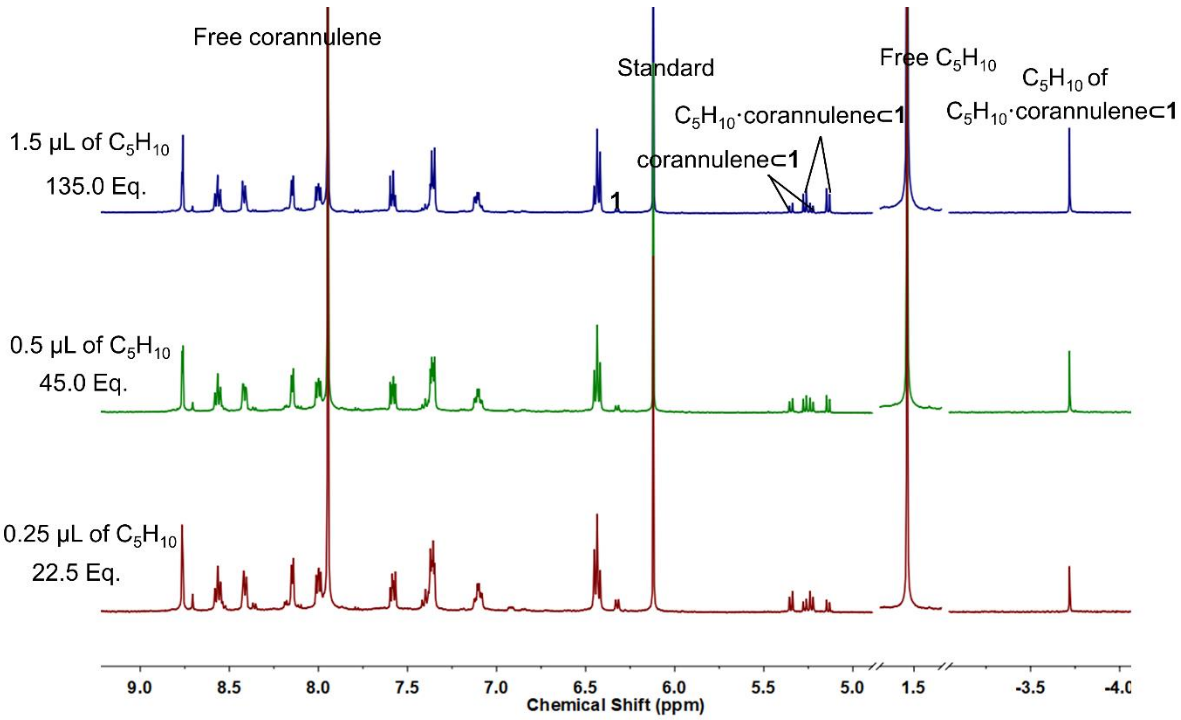

Supplementary Figure 70. ^{1}H NMR spectra of cage **1** (0.42 mM) with 5 equivalent corannulene guest and different amounts of cyclopentane (C_5H_{10}) guest (CD_3CN, 500 MHz, 298 K). The peaks of free corannulene, empty cage **1**, free C_5H_{10}, host-guest complexes corannulene⊂**1**, C_5H_{10}•corannulene⊂**1** and the trimethoxybenzene standard are labelled. The peaks of the host in corannulene⊂**1** and C_5H_{10}•corannulene⊂**1** are overlapped. However, the peaks for encapsulated corannulene in corannulene⊂**1** and C_5H_{10}•corannulene⊂**1** are distinguishable from each other. They were used to

determine the concentrations of corannulene⊂**1** and C_5H_{10}•corannulene⊂**1**. The average binding constants $K_{a1} = \frac{[HG1]}{[H][G1]}$ and $K_{a2} = \frac{[HG1G2]}{[HG1][G2]}$, where the G1 is corannulene and G2 is C_5H_{10}, were evaluated to be $(1.69\pm0.29)\times10^3$ M^{-1} and 220 ± 21 M^{-1}.

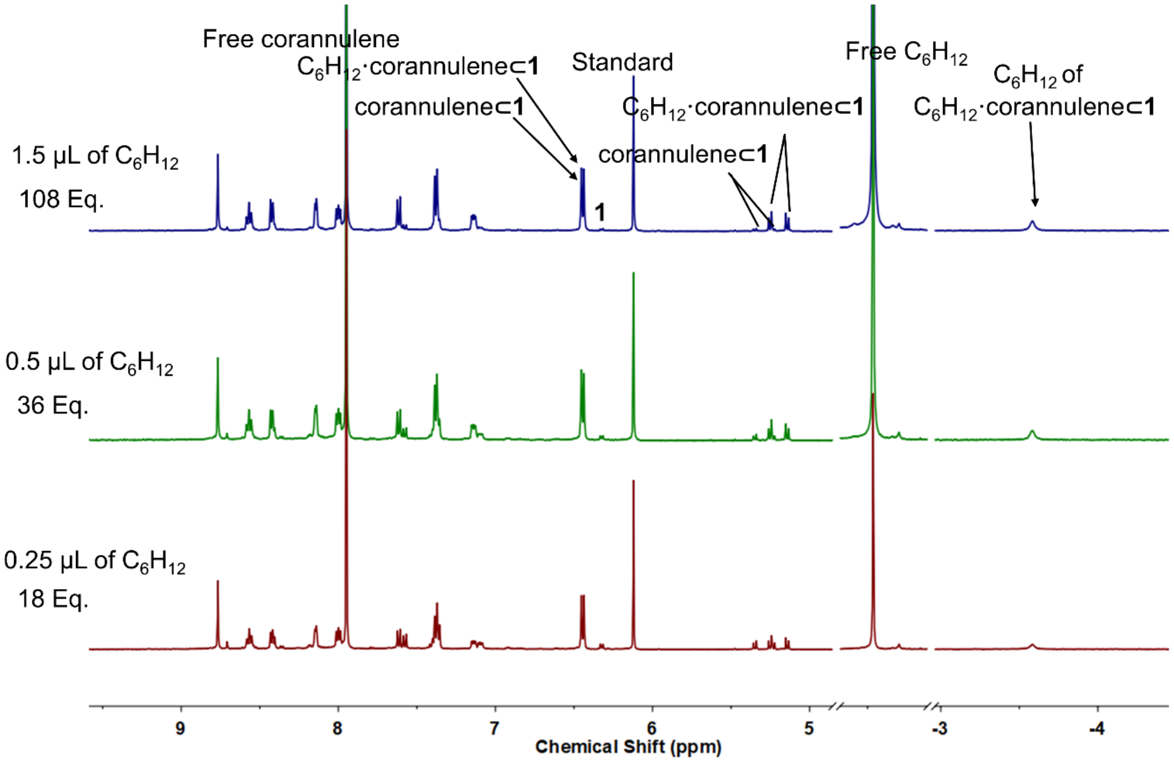

Supplementary Figure 71. ^{1}H NMR spectra of cage **1** (0.42 mM) with 5 equivalents of corannulene guest and different amounts of cyclohexane (C_6H_{12}) guest (CD_3CN, 500 MHz, 298 K). The peaks of free corannulene, empty cage **1**, free C_6H_{12}, host-guest complexes corannulene⊂**1**, C_6H_{12}•corannulene⊂**1** and the trimethoxybenzene standard are labelled. The peaks for the hosts of corannulene⊂**1** and C_6H_{12}•corannulene⊂**1** are overlapped. However, the peaks for the encapsulated corannulene of corannulene⊂**1** and C_6H_{12}•corannulene⊂**1** are distinguishable from each other. They were used to determine the concentrations of corannulene⊂**1** and C_6H_{12}•corannulene⊂**1**. The average binding constants $K_{a1} = \frac{[HG1]}{[H][G1]}$ and $K_{a2} = \frac{[HG1G2]}{[HG1][G2]}$, where the G1 is corannulene and G2 is C_6H_{12}, were evaluated to be $(1.05\pm0.14)\times10^3$ M^{-1} and 611 ± 66 M^{-1}.

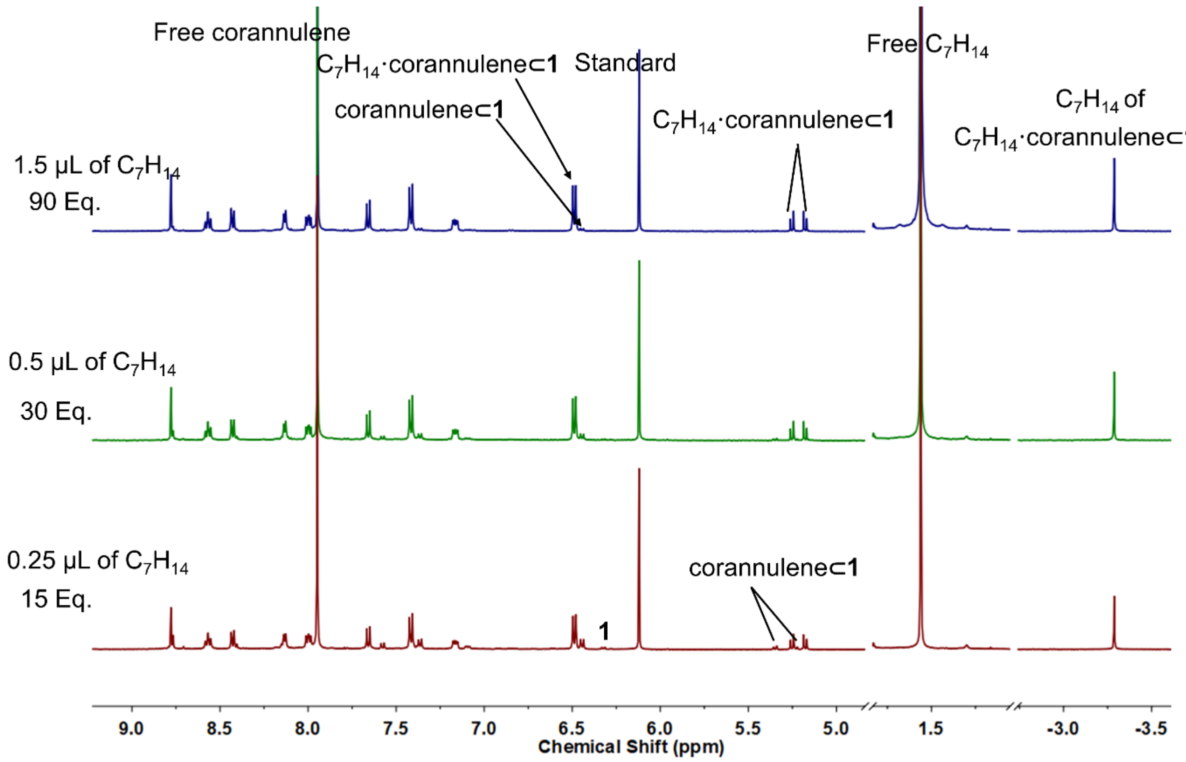

Supplementary Figure 72. ^{1}H NMR spectra of cage **1** (0.42 mM) with 5 equivalents of corannulene guest and different amounts of cycloheptane (C_7H_{14}) guest (CD_3CN, 500 MHz, 298 K). The peaks of free corannulene, empty cage **1**, free C_7H_{14}, host-guest complexes corannulene⊂**1**, C_7H_{14}•corannulene⊂**1** and trimethoxybenzene standard are labelled. The peaks of the hosts in corannulene⊂**1** and C_7H_{14}•corannulene⊂**1** are distinguishable from each other. They were used to determine the concentrations of corannulene⊂**1** and C_7H_{14}•corannulene⊂**1**. The average binding constants $K_{a1} = \frac{[HG1]}{[H][G1]}$ and $K_{a2} = \frac{[HG1G2]}{[HG1][G2]}$, where the G1 is corannulene and G2 is C_7H_{14}, were evaluated to be $(1.61 \pm 0.27) \times 10^{3}$ M^{-1} and 883 ± 170 M^{-1}.

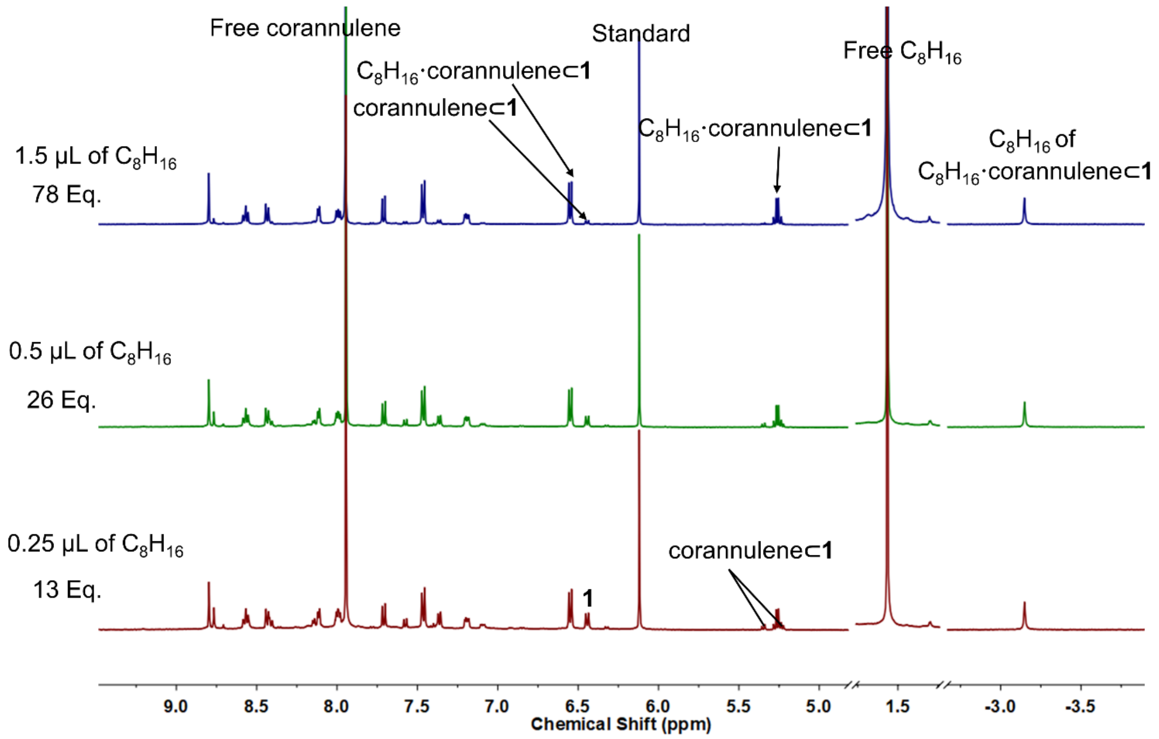

Supplementary Figure 73. ^{1}H NMR spectra of cage **1** (0.42 mM) with 5 equivalents corannulene guest and different amounts of cyclooctane (C_8H_{16}) guest (CD_3CN, 500 MHz, 298 K). The peaks of free corannulene, empty cage **1**, free C_8H_{16}, host-guest complexes corannulene⊂**1**, C_8H_{16}•corannulene⊂**1** and the trimethoxybenzene standard are labelled. The peaks of the hosts of corannulene⊂**1** and C_8H_{16}•corannulene⊂**1** are distinguishable from each other. They were used to determine the concentrations of corannulene⊂**1** and C_8H_{16}•corannulene⊂**1**. The average binding constants $K_{a1} = \frac{[HG1]}{[H][G1]}$ and $K_{a2} = \frac{[HG1G2]}{[HG1][G2]}$, where the G1 is corannulene and G2 is C_8H_{16}, were evaluated to be $(1.98\pm 0.31)\times10^3$ M^{-1} and 547 ± 46 M^{-1}.

Supplementary Table 1. The summarized binding constants of corannulene (G_1) and cycloalkanes (G_2: C_5H_{10} - C_8H_{16}).

	C_5H_{10}·corannulene⊂1	C_6H_{12}·corannulene⊂1	C_7H_{14}·corannulene⊂1	C_8H_{16}·corannulene⊂1
K_{a1} (M^{-1})	$(1.69\pm0.29)\times10^3$	$(1.05\pm0.14)\times10^3$	$(1.61\pm0.27)\times10^3$	$(1.98\pm0.31)\times10^3$
K_{a2} (M^{-1})	220 ± 21	611 ± 66	883 ± 170	547 ± 46

Note: The average binding constants are defined as $K_{a1} = \frac{[HG1]}{[H][G1]}$ and $K_{a2} = \frac{[HG1G2]}{[HG1][G2]}$, where the G1 is corannulene, G2 is the cycloalkane (C_5H_{10} - C_8H_{16}), HG1 is the host-guest complex with G1 as guest and HG1G2 is host-guest complex with G1 and G2 as guest.

4. Dynamic studies of the guests inside cage 1

In this part, variable-temperature ^{1}H NMR has been performed for each sample. Line-shape analysis has been applied to estimate the exchange between protons using Bruker Topspin 3.2.[2] The protons H_q and $H_{q`}$ belong to the two independent bond-connected neighbouring protons of the encapsulated corannulene. When the bowl inverts, they will exchange their positions relative to the host. The simulation is based on two coupled nuclei exchanging their positions. Due to the low boiling point of the acetonitrile (355 K), we could not observe total coalescence of the peaks. But the data allow estimation of the activation energy of inversion. At high temperature, the guests are partially released from the host (K_a is smaller). The broadening of the guest signals does not relate to the guest exchange. Firstly, when the signals for the encapsulated corannulene broaden, the host signals of corannulene⊂**1** remain sharp. Secondly, in the hetero-guest ternary systems, at higher temperature, corannulene is also observed to be partially released from the host (with smaller K_a), but the signals of the encapsulated corannulene remain sharp while the second guests reduce the bowl-to-bowl inversion. Finally, EXSY (NOESY) experiments are consistent with the exchange of the H_q and $H_{q`}$ protons, but no exchange between protons of empty **1** and corannulene⊂**1** is observed. If the guest exchange was occurring, we would expect to see cross-peaks between the host and the host-guest complex in EXSY.[3]

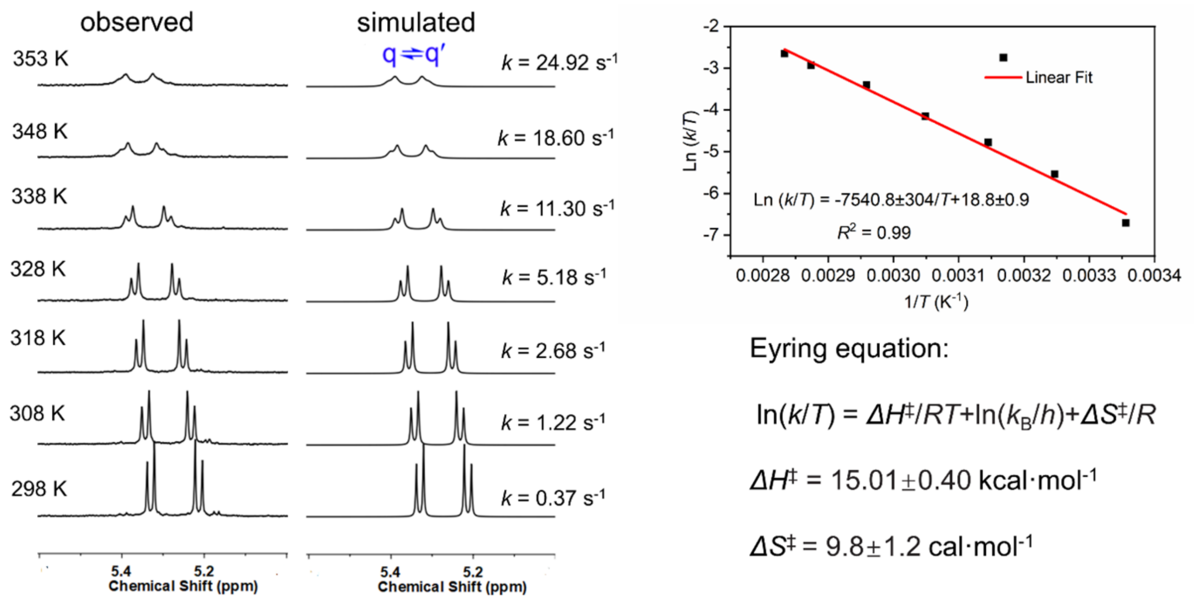

Eyring equation:

$\ln(k/T) = \Delta H^{\ddagger}/RT + \ln(k_B/h) + \Delta S^{\ddagger}/R$

$\Delta H^{\ddagger}$ = 15.01±0.40 kcal·mol^{-1}

$\Delta S^{\ddagger}$ = 9.8±1.2 cal·mol^{-1}

Supplementary Figure 74. Right: The encapsulated corannulene region of the variable temperature ^{1}H NMR spectra (CD_3CN, 500 MHz) of Cage **1** with corannulene guest (parts of **Supplementary Figure 67**) and the corresponding simulated ones obtained by line-shape analyses. The line-shape analyses were obtained using Bruker Topspin 3.2. Left: for the inversion of corannulene in corannulene⊂**1**.

According to the Eyring equation ln(k/T) = $\Delta H^{‡}/RT$+ln(k_B/h)+$\Delta S^{‡}/R$, the $\Delta H^{‡}$ and $\Delta S^{‡}$ were determined to be 15.01±0.40 kcal·mol^{-1} and -9.8±1.2 kcal·K^{-1}·mol^{-1}, respectively. According to $\Delta G^{‡} = \Delta H^{‡}-T\Delta S^{‡}$, we could estimate the $\Delta G^{‡}$(298K) = 17.94±0.27 kcal·mol^{-1}.

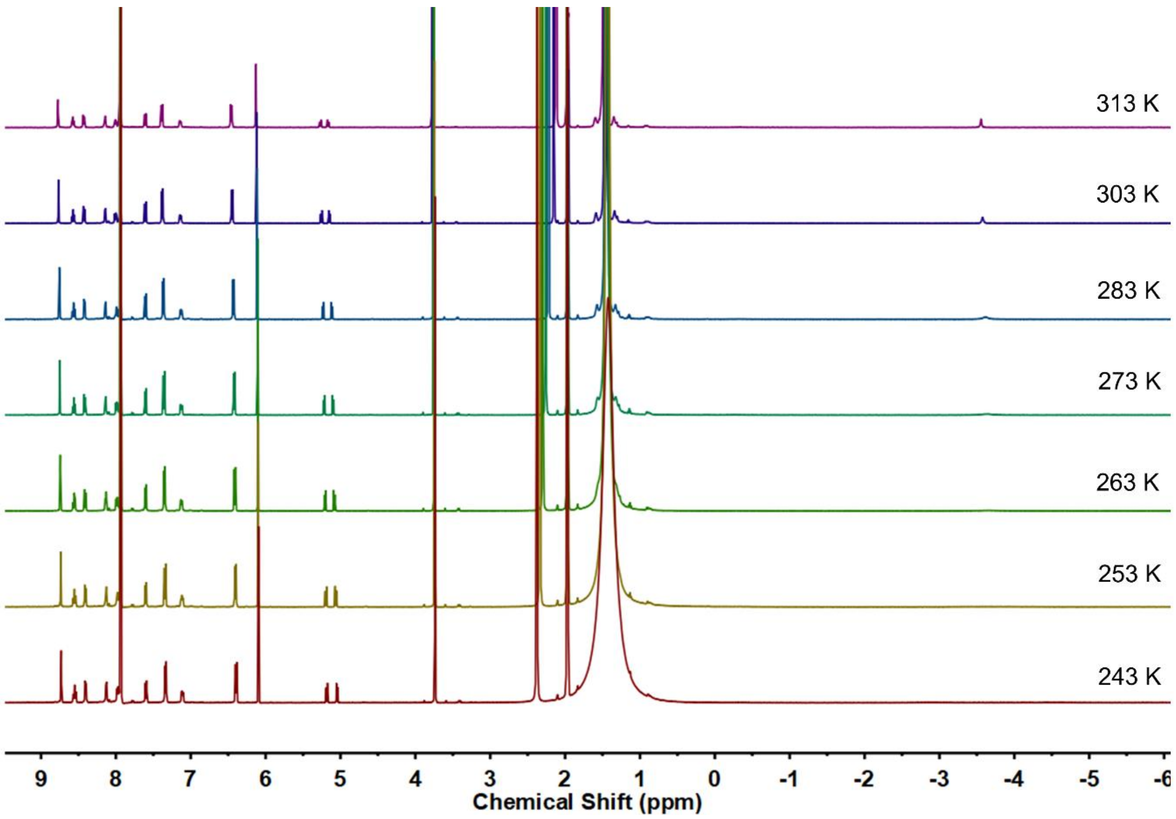

Supplementary Figure 75. Low temperature VT ^{1}H NMR spectra of C_6H_{12}•corannulene⊂**1** (CD_3CN, 500 MHz). The concentration of the **1** is 0.5 mM. And there are 14 equivalents of corannulene and about 280 equivalents of C_6H_{12} in the solution.

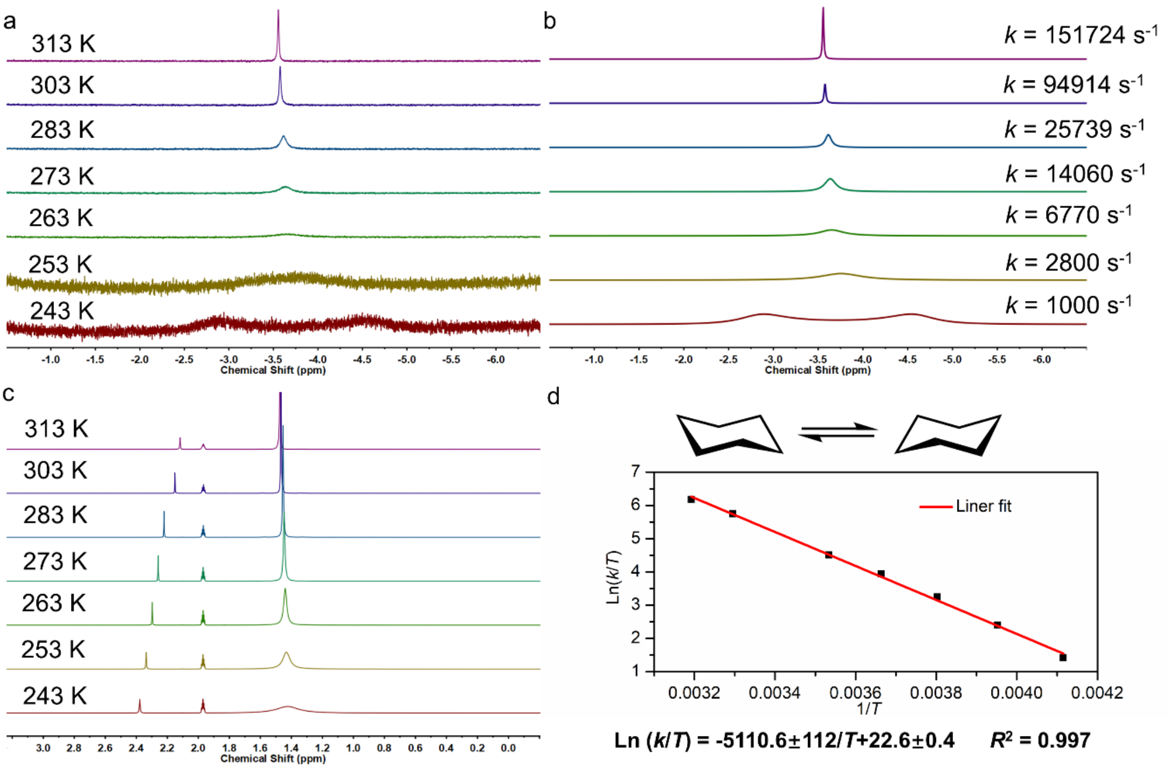

Supplementary Figure 76. Enlargements of the VT ^{1}H NMR spectra of C_6H_{12}•corannulene⊂**1** for a) the region of encapsulated cyclohexane, b) the corresponding simulated ones obtained by line-shape analysis, c) the region of the excess free cyclohexane (CD_3CN, 500 MHz). d) Eyring plot for cyclohexane chair flipping inside C_6H_{12}•corannulene⊂**1**. According to the Eyring equation $\ln(k/T) = \Delta H^{‡}/RT + \ln(k_B/h) + \Delta S^{‡}/R$, the $\Delta H^{‡}$ and $\Delta S^{‡}$ were determined to be 10.17±0.15 kcal·mol^{-1} and -2.35±0.53 cal·K^{-1}·mol^{-1}, respectively. According to $\Delta G^{‡} = \Delta H^{‡}-T\Delta S^{‡}$, we could estimate $\Delta G^{‡}$(206.5K) = 10.65±0.03 kcal·mol^{-1}. This is a hypothetical value since this temperature cannot be reached in MeCN. We observed that the free C_6H_{12} in the same NMR spectra remained a singlet (**Supplementary Figure 76**c). For comparison, the only reported data of free C_6H_{12} is that C_6H_{12} splits at 206.5 K in CS_2 with an energy barrier of $\Delta G^{‡}$(206.5K) = 9.70 kcal·mol^{-1}.[4]

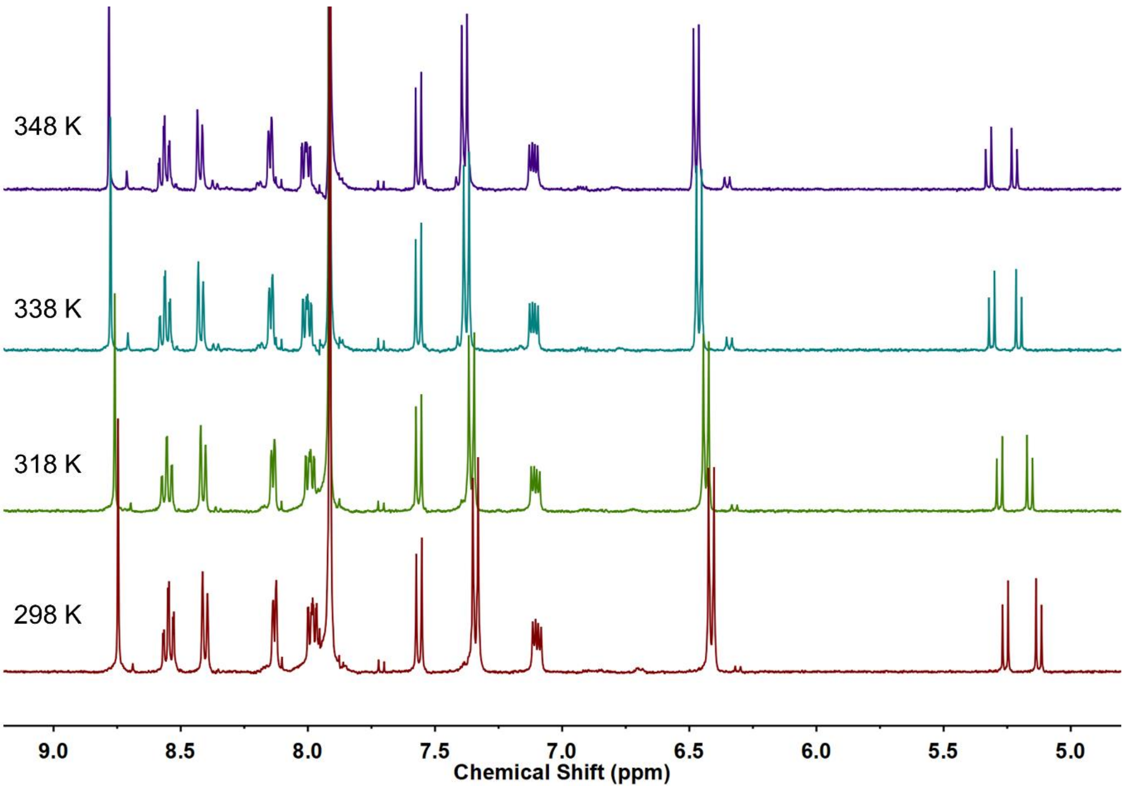

Supplementary Figure 77. High-temperature VT ^{1}H NMR spectra of C_5H_{10}•corannulene⊂**1** (CD_3CN, 400 MHz).

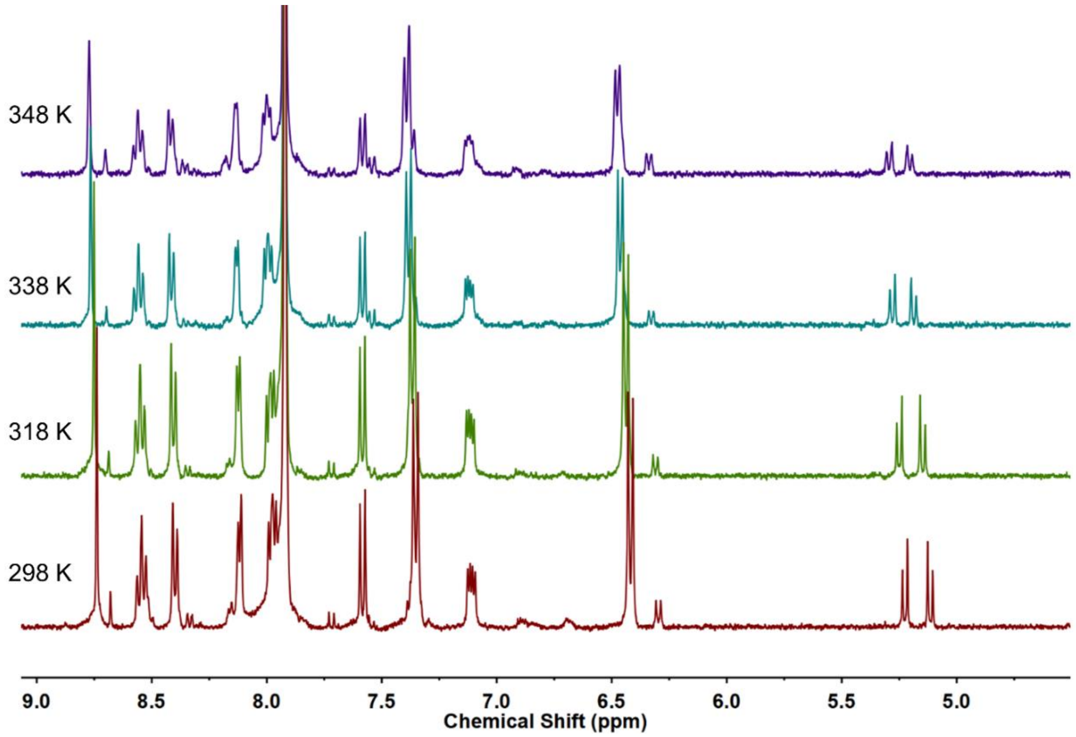

Supplementary Figure 78. High-temperature VT ^{1}H NMR spectra of C_6H_{12}•corannulene⊂**1** (CD_3CN, 400 MHz).

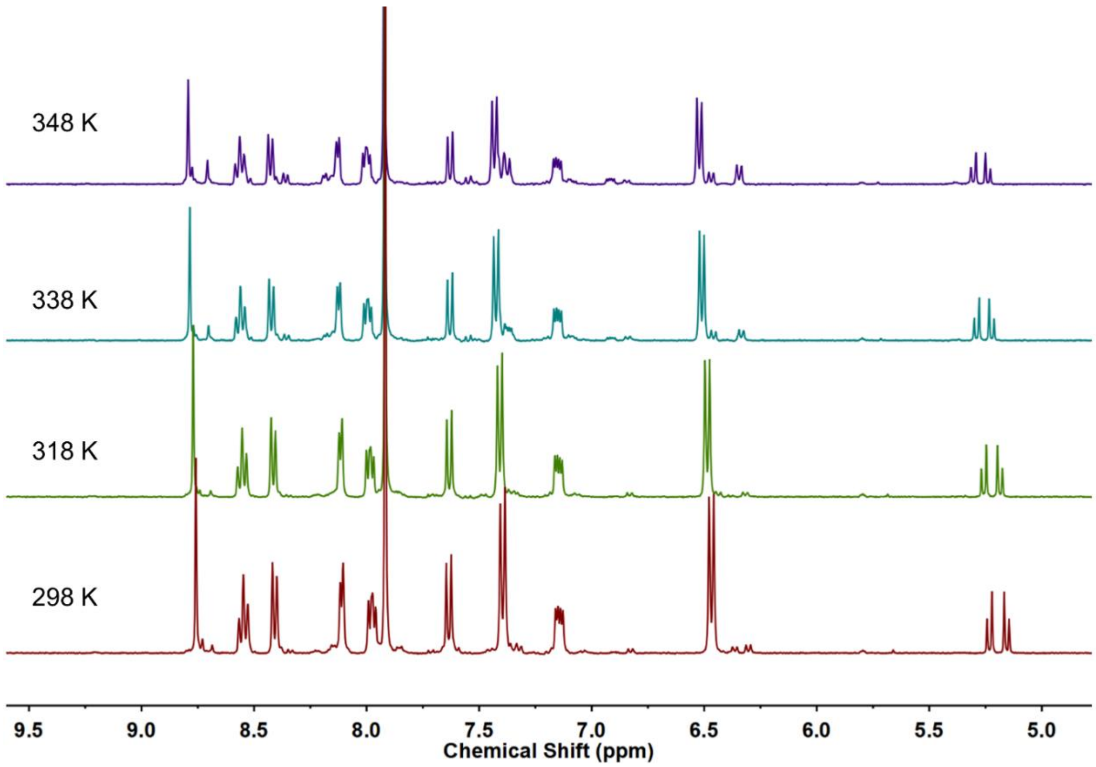

Supplementary Figure 79. High-temperature VT ^{1}H NMR spectra of C_7H_{14}•corannulene⊂**1** (CD_3CN, 400 MHz).

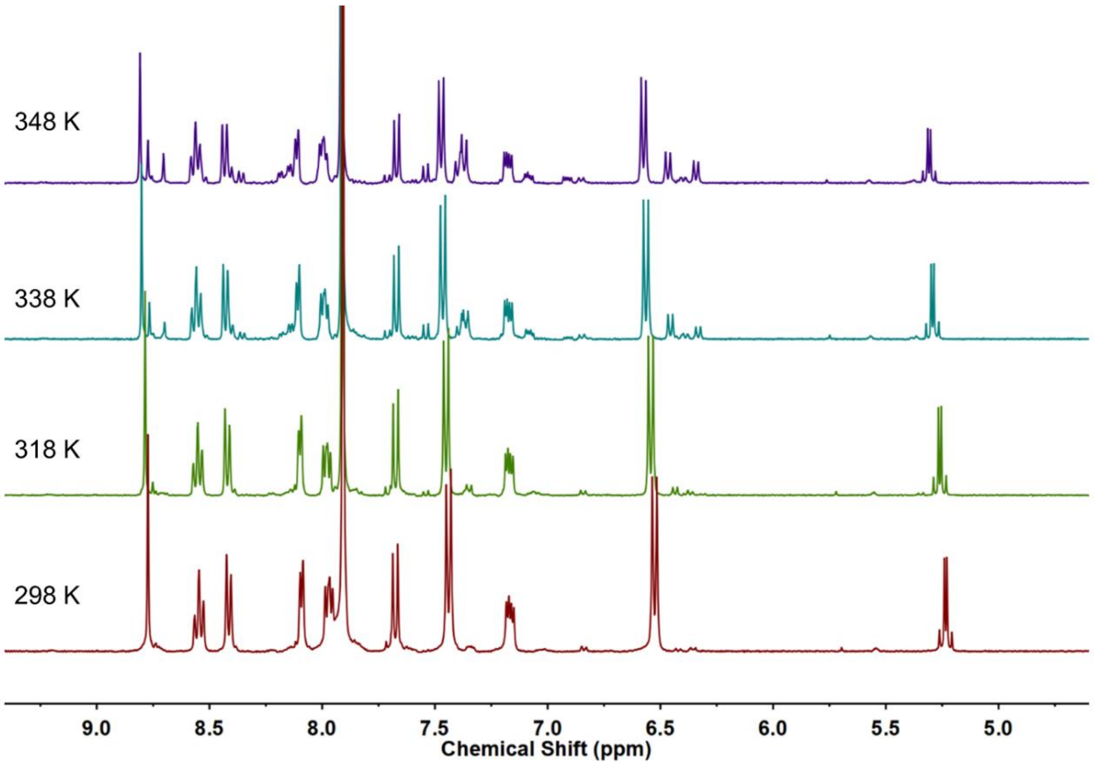

Supplementary Figure 80. High-temperature VT ^{1}H NMR spectra of C_8H_{16}•corannulene⊂**1** (CD_3CN, 400 MHz).

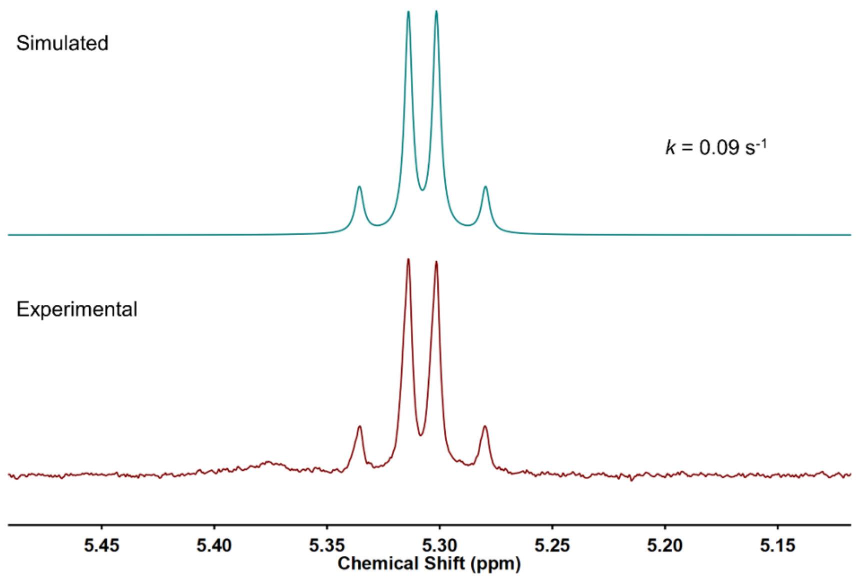

Supplementary Figure 81. Comparison of the ^{1}H NMR region of the encapsulated corannulene of C_8H_{16}•corannulene⊂**1** at 348 K (bottom) and the corresponding simulated one obtained by line-shape analysis (top). No obvious broadening of the signals of encapsulated corannulene was observed by VT NMR. The changes of the exchange constant *k* are very small, thus making preventing the construction of a meaningful Eyring plot. However, we could roughly estimate the activation energy by using the Eyring equation ($\Delta G^{\ddagger} = -RT \ln (kh/k_B T)$ where, *h* is Planck's constant, k_B is Boltzmann constant, *R* is gas constant). The simulated spectrum gives an exchange rate constant *k* of 0.09 s^{-1}. According to the Eyring equation, the $\Delta G^{\ddagger}$ (348 K) is calculated to be 22.17 kcal·mol^{-1}. The value of corannulene⊂**1** at 348 K is calculated by $\Delta G^{\ddagger} = \Delta H^{\ddagger}-T\Delta S^{\ddagger}$ (detailed in **Supplementary Figure 76**) to be 18.43±0.22 kcal·mol^{-1}. Thus, the $\Delta\Delta G^{\ddagger}$ (348 K) is 3.74±0.22 kcal·mol^{-1}.

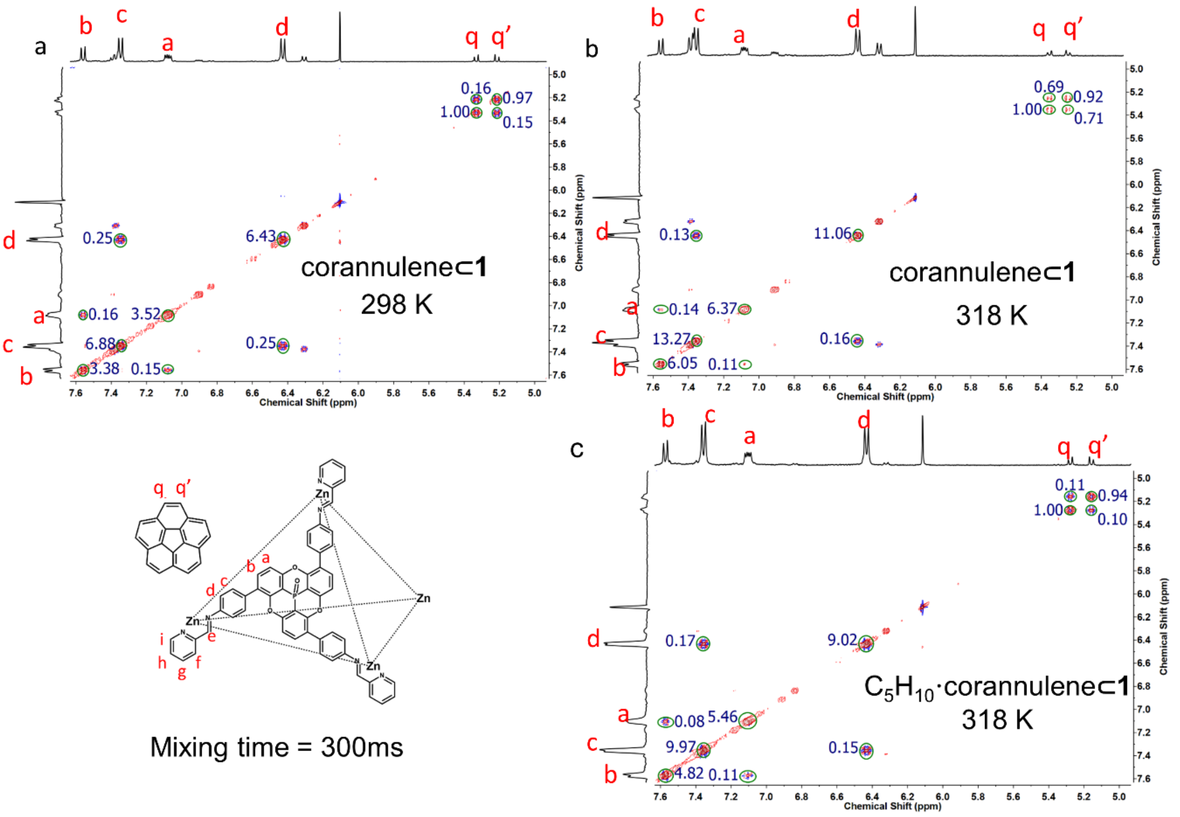

Supplementary Figure 82. Comparison of the 2D exchange spectroscopy (EXSY) of corannulene⊂**1** at 298 K (a), 318 K (b) and C_5H_{10}•corannulene⊂**1** at 318 K (c). The spectra were obtained using the same parameters as the NOESY, but adjusting the mixing time (T_m) to be 300 ms. The dynamic exchange rate constants (k) of two protons are related to the 2D integrated intensities (I) of the diagonal peaks, by equation $k = \frac{1}{Tm} \ln \frac{r+1}{r-1}$ in which $r = (I_{aa}+I_{bb})/(I_{ab}+I_{ba})$.[5] According to this equation, the larger the I_{ab} and I_{ba}, the smaller r will be. The smaller r, the larger k will be. However in our case, it is more complicated to quantify the k by EXSY. Since the two protons H_q and $H_{q'}$ are adjacent, the NOE effects and scalar coupling can interfere with the EXSY signals. Nonetheless the spectra here clearly show that there is the exchange between H_q and $H_{q'}$. Qualitative analysis is also informative. The r value of H_q and $H_{q`}$ is obviously much smaller than that of the referenced peaks (H_a and H_b) and (H_c and H_d). Comparing **Supplementary Figure 82a** and **Supplementary Figure 82b**, upon raising the temperature, the r of H_q and $H_{q'}$ becomes smaller, leading to larger k, in line with the observation from the line shape analyses. Comparing **Supplementary Figure 82b** and **Supplementary Figure 82c**, in the presence of the second guest cyclopentane, the exchange rate k is suppressed with a larger r, also in line with the observation from the line shape analyses. The second guest thus suppresses the exchange of H_q and $H_{q`}$.

5. Enantiomeric excess value determinations by NMR

5.1 Procedure for determination of the *e.e.* value of 3-methyl-2-butanol by NMR

1. Preparation of enantiopure cage (P_4-Λ_4)**1**.

   The procedure is the same as the racemic cage, using enantiopure *P*-**A** in place of the racemic subcomponent.

2. Encapsulation of the *R*/*S*-3-methyl-2-butanol inside (P_4-Λ_4)**1**.

1.0 mg (0.00017 mmol) (P_4-Λ_4)**1** was dissolved in 0.35 mL CD_3CN, and 0.3 mg (0.0012 mmol, 7 equivalents) corannulene was added. A 50 μL solution of CD_3CN containing 4 μL (325 equivalents) of *R*/*S*-3-methyl-2-butanol was added to the above solution. The solution was kept at 70°C overnight. The sample was cooled to room temperature for 1 hour before NMR determination.

3. NMR determinations.

The samples were analysed using a high-resolution Bruker 500MHz TCI Cryoprobe NMR. The receiver gain (RG) was set at double the value determined by the automatic receiver gain (RGA), to increase the signal to noise ratio of the encapsulated guest. The accurate integrations of the peaks of the encapsulated guests were obtained by deconvolutions of the ^{1}H NMR spectra, as shown in the following figure.

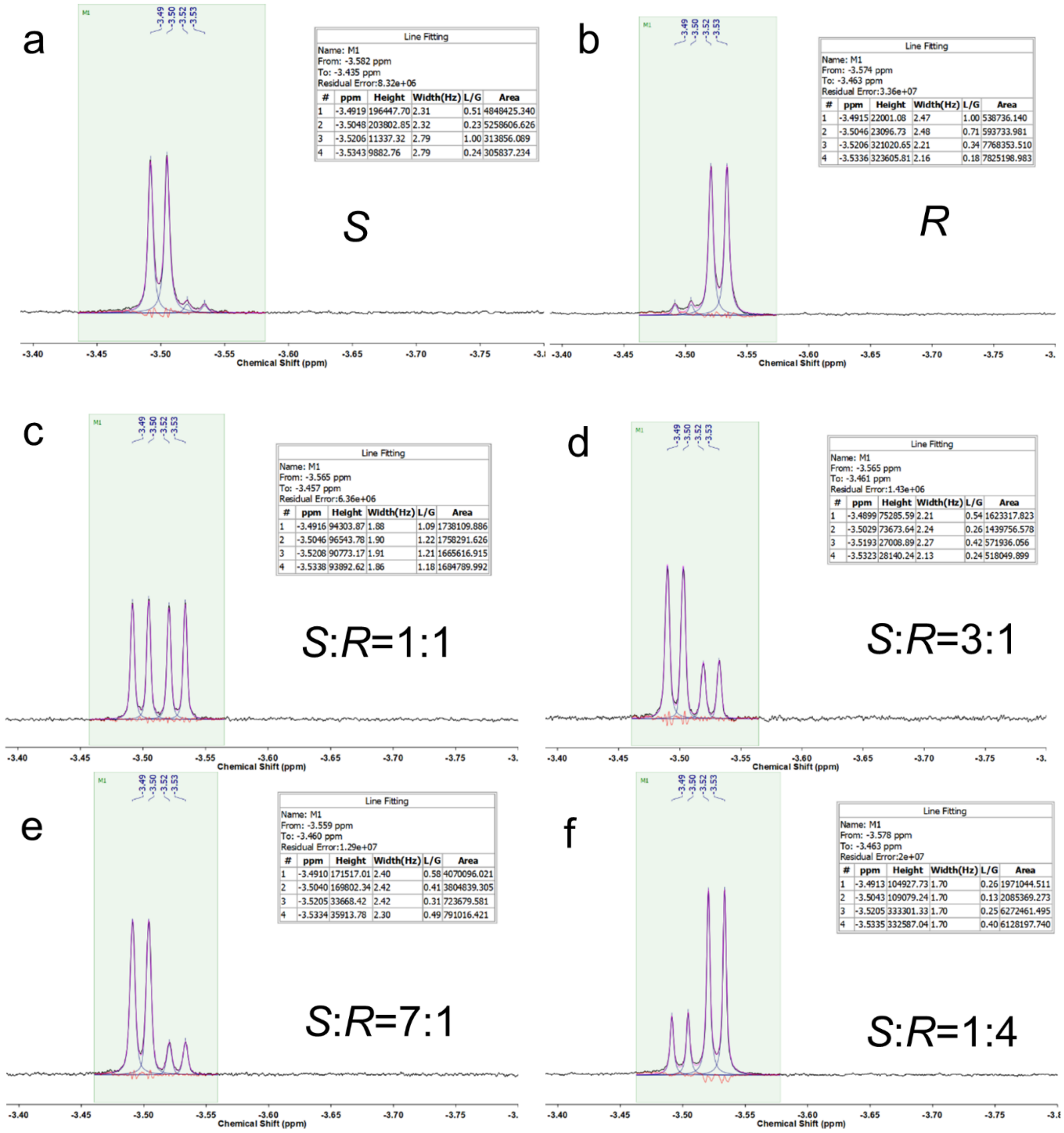

Supplementary Figure 83. Deconvolution of the ^{1}H NMR spectra of the encapsulated 3-methyl-2-butanol inside enantiopure cage corannulene⊂(P_4-Λ_4)**1**. The area values were used as integrations to calculate the *R*:*S* ratio. The enantiopurity of the chiral ligands was confirmed by Chiral HPLC, to be more than 99% (see Supplementary Section 8.2 below). Thus the addition of commercial *S*-3-methyl-2-butanol showed two sets of peaks due to the presence of a small amount of *R*-3-methyl-2-butanol inside. According to the integrations, the commercial *S*-3-methyl-2-butanol contains 94.2% *S*-3-methyl-2-butanol and 5.8% *R*-3-methyl-2-butanol. Likewise the commercial *R*-3-methyl-2-butanol, was found to contain 93.2% *R*-3-methyl-2-butanol and 6.8% *S*-3-methyl-2-butanol. The calculated *e.e.* values based on the expected enantiopurities of the different samples of 3-methyl-2-butanol and the determined *e.e.* values by NMR integration are shown in the following **Supplementary Table 2**.

Supplementary Table 2. Comparison of the calculated *e.e.* values based on the purities of 3-methyl-2-butanol and the determined *e.e.* values by NMR integration.

Volume Ratio (*S*:*R*)	Cal. *e.e.* Value (%)	NMR Integrated Ratio (*S*:*R*)	NMR *e.e.* Value (%)	*e.e.* Difference (%)
1:1	1.0	1.04:1	2.0	1
3:1	48.1	2.81	47.5	0.6
7:1	71.7	5.20	67.7	4
1:4	-55.5	3.06	-50.7	4.8

Note: Pure *S*-3-methyl-2-butanol is defined to have *e.e.* 100% and pure *R*-3-methyl-2-butanol is defined to have *e.e.* -100%. The purity of commercial *R*-3-methyl-2-butanol and *S*-3-methyl-2-butanol is about 93.2% and 94.2%, respectively, according to the integrations of enantiopure cage (P_4-Λ_4)**1** interacting with them. The calculated *e.e.* Value is based on these purities.

6. Circular Dichroism studies

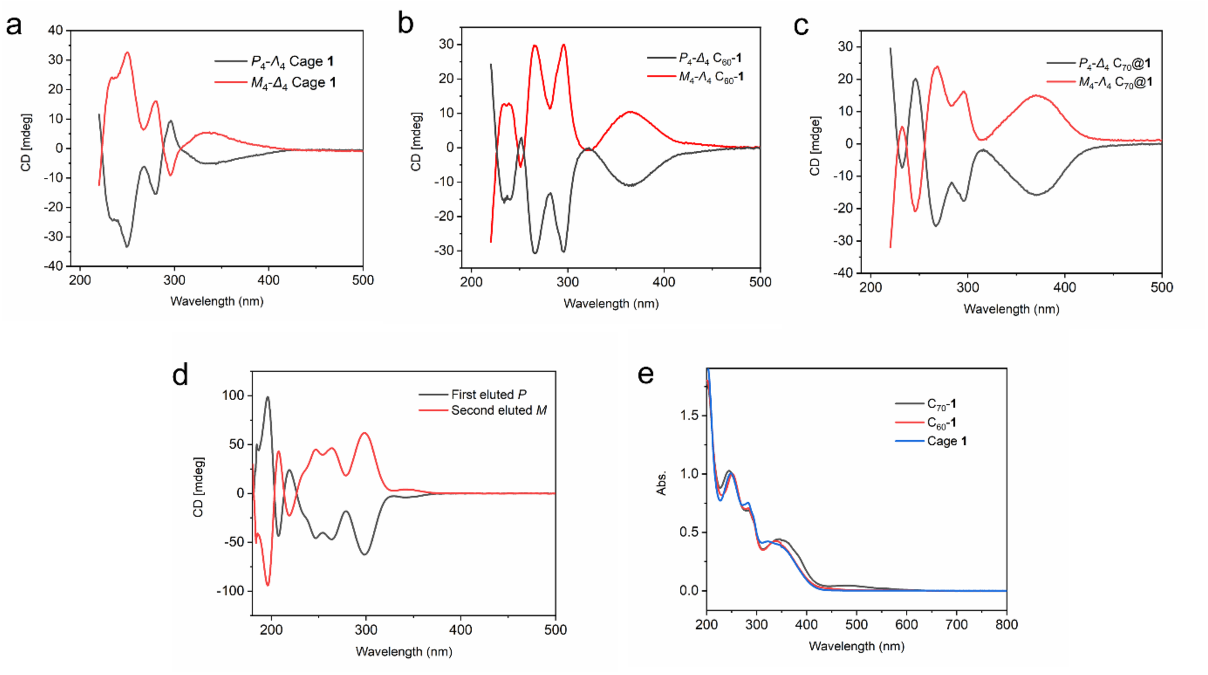

Supplementary Figure 84. CD spectra of 0.03 mM acetonitrile solutions of cage **1** assembled with a) the first eluted *P* enantiomeric ligands (red, 1st eluted P_4-Λ_4 Cage **1**) and the second eluted *M* enantiomeric ligands (black, 2nd eluted M_4-Δ_4 Cage **1**); the corresponding cages encapsulating b) C_{60} inside and c) C_{70} inside; d) the CD spectra of first (black, *P* isomer) and second (red, *M* isomer) eluted enantiomeric ligands **A** (0.202 mM in acetonitrile); e) the UV-vis absorption spectra of the corresponding cages and host-guest complexes. The second eluted enantiomeric ligand was determined to be the *M* isomer by DFT calculation (see section 8).

7. Volume calculations

The voids enclosed by the host frameworks have been determined by VOIDOO calculations,[6] based on their crystal structures. For C_{60}⊂**1** and C_{70}⊂**1**, the fullerene guests have been removed. A virtual probe with a radius of 1.7 Å was employed in all cases. Probes smaller than 1.7 Å were observed to exit the cavity during the calculation for C_{60}⊂**1** and C_{70}⊂**1**. The standard parameters tabulated below were used in all cases, following the previously published procedure.[7]

Maximum number of volume-refinement cycles: 30

Minimum size of secondary grid: 3

Grid for plot files: 0.2

Primary grid spacing: 0.1

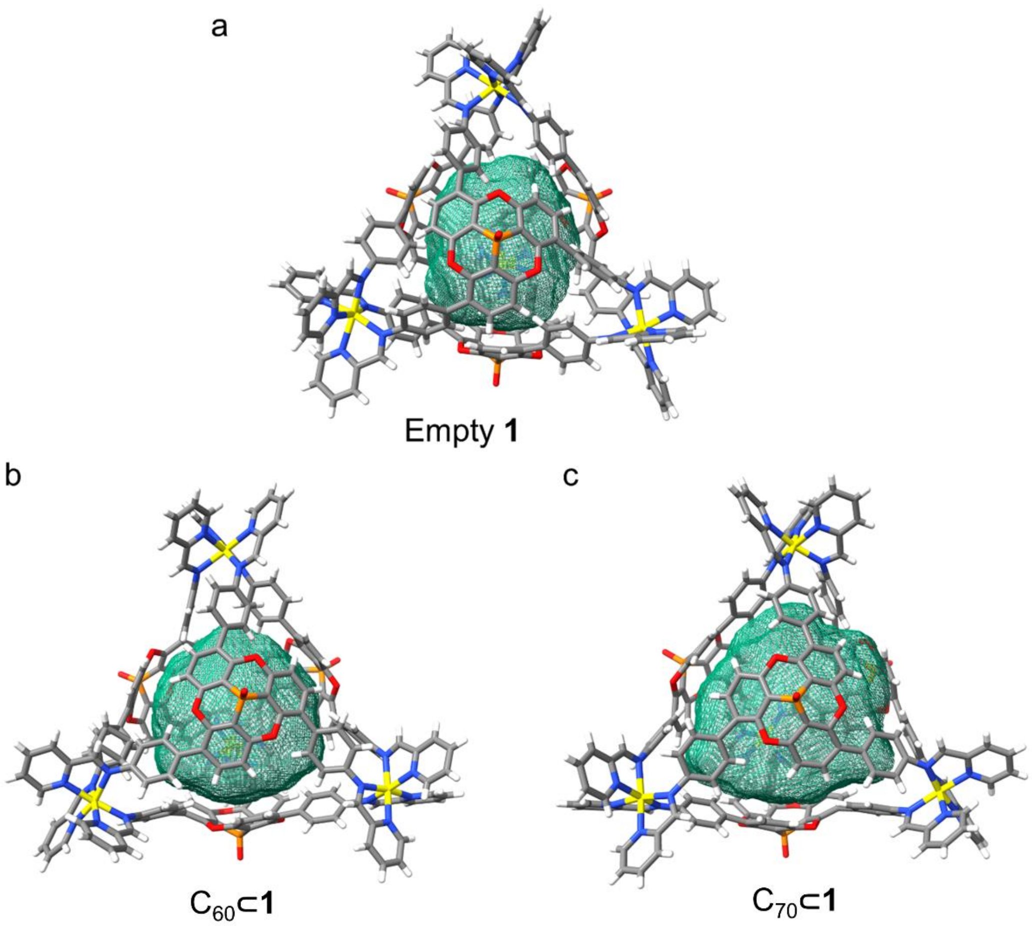

Supplementary Figure 85. VOIDOO-calculated void space (green mesh) within a) empty **1** (volume: 498.9 Å^3); b) C_{60}⊂**1** (volume: 718.4 Å^3); c) C_{70}⊂**1** (volume: 924.9 Å^3) based on their corresponding crystal structures with internal guests removed. Only one of the crystallographically distinct cages in the structure of empty **1** is shown and used for the calculation.

8. Chiral resolution of subcomponent A and assignment of the configurations

8.1 Analytical chiral HPLC separation for compound A

The separation procedure: The racemic subcomponent **A** was dissolved in dichloromethane, injected onto the chiral column, and detected with a UV detector at 254 nm, a polarimeter and circular dichroism at 254 nm. The flow-rate was 1 mL·min^{-1}.

Column	Mobile Phase	t1	k1	t2	k2	α	Rs
**Chiralpak IA**	Heptane / ethanol / dichloromethane (30/30/40)	4.84 (-)	0.64	6.24 (+)	1.11	1.74	4.46

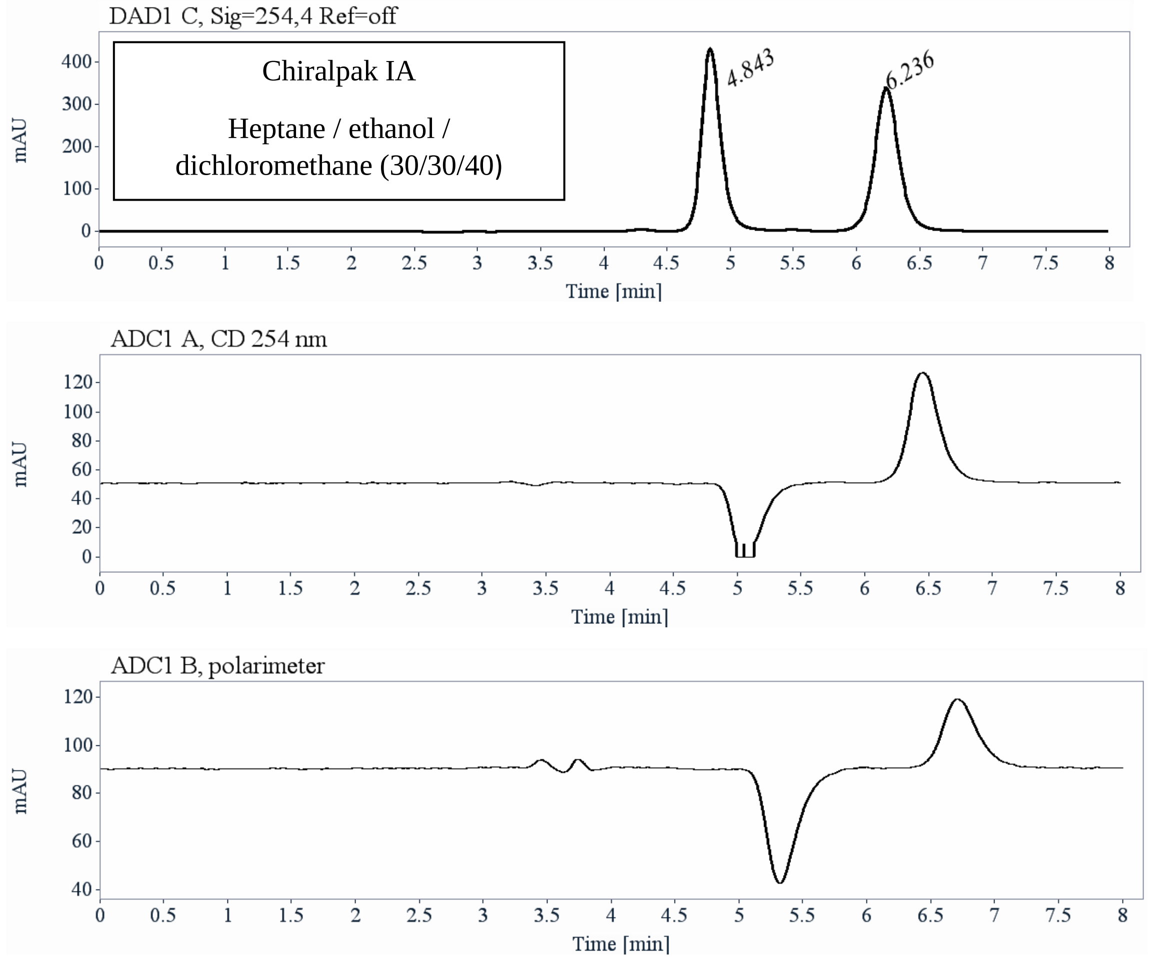

RT [min]	Area	Area%	Capacity Factor	Enantioselectivity	Resolution (USP)
4.84	4650	49.81	0.64		
6.24	4685	50.19	1.11	1.74	4.46
Sum	9334	100.00			

8.2 Preparative separation for compound A:

The separation procedure: 1) Sample preparation: About 26 mg of compound **A** was dissolved in 20 mL of a mixture of ethanol / dichloromethane (50/50). 2) Chromatographic conditions: Chiralpak IA (250 x 10 mm), hexane / ethanol / dichloromethane (30/30/40) as mobile phase, flow-rate = 5 mL·min^{-1}, UV detection at 254 nm.3) Injections (stacked): 34 times 600 µL, every 7 minutes.

Result

1) First fraction (*P*-L): 11 mg of the first eluted enantiomer with ee > 99.5%

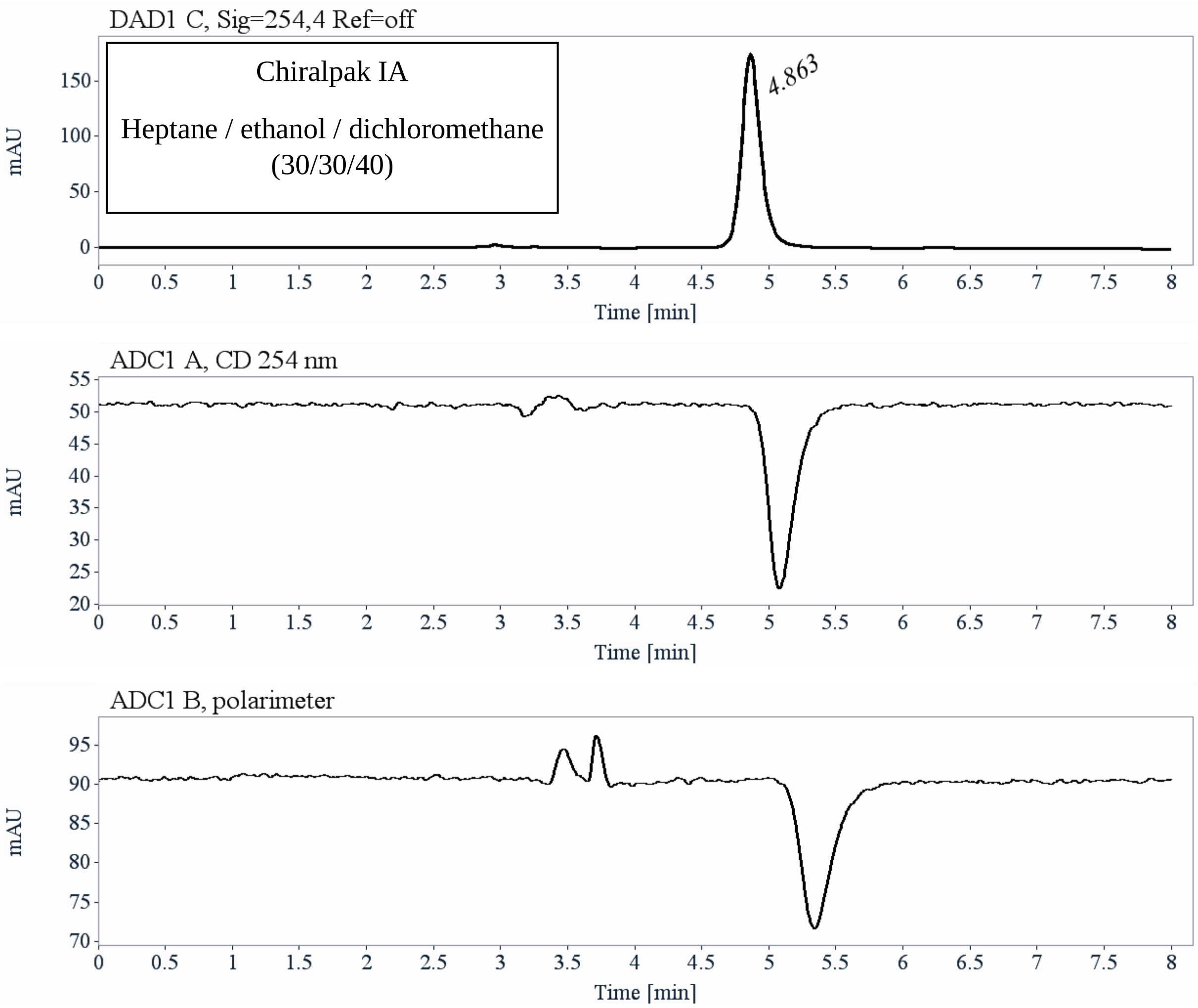

RT [min]	Area	Area%
4.86	1776	100.00
Sum	1776	100.00

2) Second fraction (*M*-**A**): 11 mg of the second eluted enantiomer with ee > 99.5%

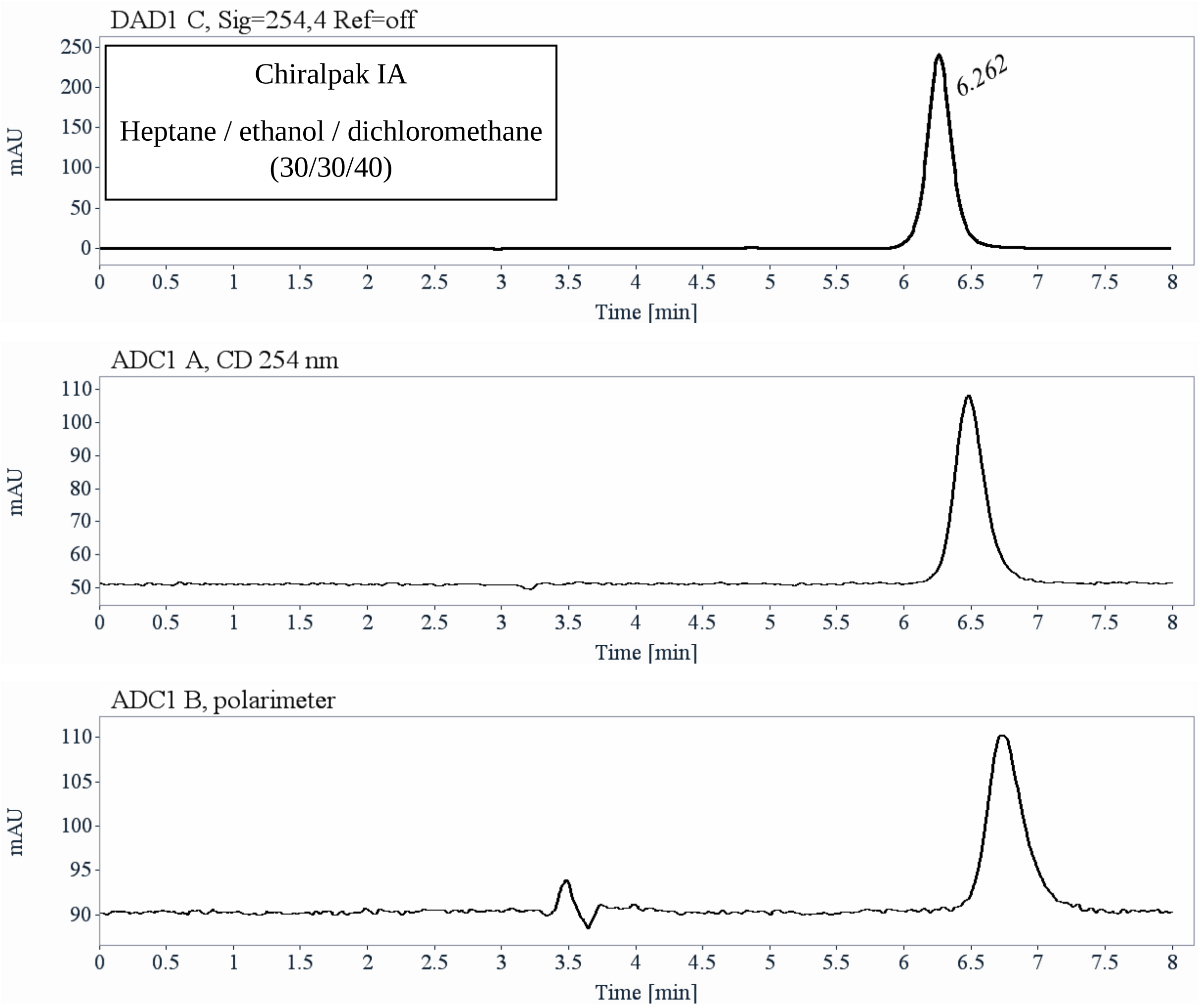

RT [min]	Area	Area%
6.26	3221	100.00
Sum	3221	100.00

8.3 Optical rotations

Optical rotations were measured on a Jasco P-2000 polarimeter with a halogen lamp (589, 578, 546 and 436 nm), in a 10 cm cell, thermostated at 25 °C with a Peltier controlled cell holder.

Supplementary Table 3. Optical rotation parameters for *P*-**A** and *M*-**A**.

λ (nm)	*P*-**A**<br>First eluted enantiomer on Chiralpak IA<br>$[\alpha]_{\lambda}^{25}$ (CH_2Cl_2, c =0.15)	*M*-**A**<br>Second eluted enantiomer on Chiralpak IA<br>$[\alpha]_{\lambda}^{25}$ (CH_2Cl_2, c =0.165)
589	- 870	+ 870
578	- 920	+ 920
546	- 1095	+ 1095
436	- 2210	+ 2210

8.4 Electronic Circular Dichroism

ECD and UV spectra were measured on a JASCO J-815 spectrometer equipped with a JASCO Peltier cell holder PTC-423 to maintain the temperature at 25.0±0.2°C. A CD quartz cell of 1 mm of optical pathlength was used. The CD spectrometer was purged with nitrogen before recording each spectrum, which was baseline subtracted. The baseline was always measured for the same solvent and in the same cell as the samples. The spectra are presented without smoothing and further data processing.

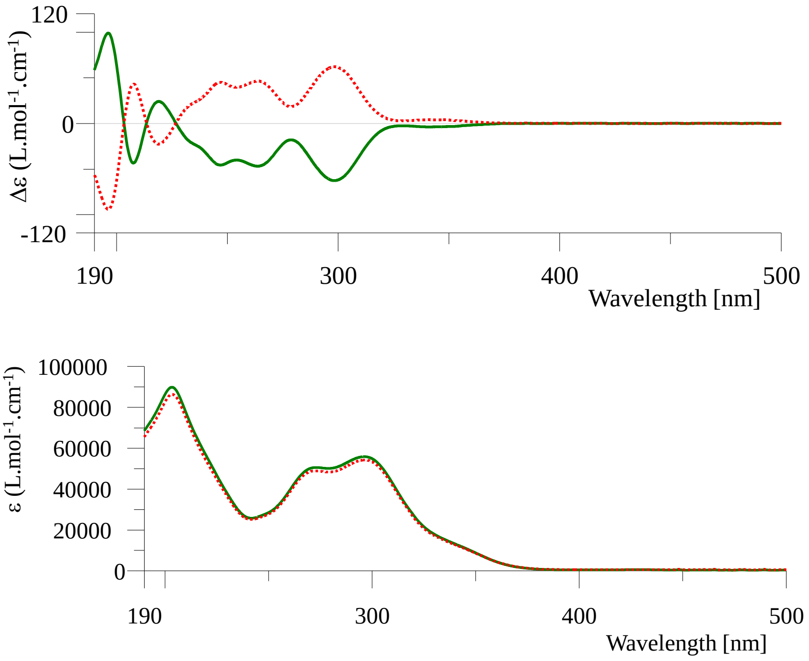

Supplementary Figure 86: Top: CD spectrum of first eluted enantiomer (green solid line, 0.202 mM in acetonitrile) and second eluted enantiomer (red dotted line, 0.187 mM in acetonitrile). Bottom: The corresponding UV-Vis absorbance.

8.5 Absolute configuration determination by comparison of calculated and experimental ECD spectra

According to the following ECD spectra calculations, the second eluted enantiomer on Chiralpak IA, with $[\alpha]_D^{25}$ (CH_2Cl_2, c =0.165) = + 870, is the (*M*)-enantiomer.

Calculations

DFT and TD-DFT calculations were performed using Gaussian 16 package[8], with the default parameters for the solvent used in SMD. Spectra were plotted with Specdis v. 1.71[9] as sum of Gaussians with σ as half the bandwidth at 1/*e* peak height.

Four conformer geometries were found for the (*M*)-enantiomer after a conformational analysis with molecular mechanics and DFT at the SMD(CH_3CN)/PBE0 /Def2TZVPP level.

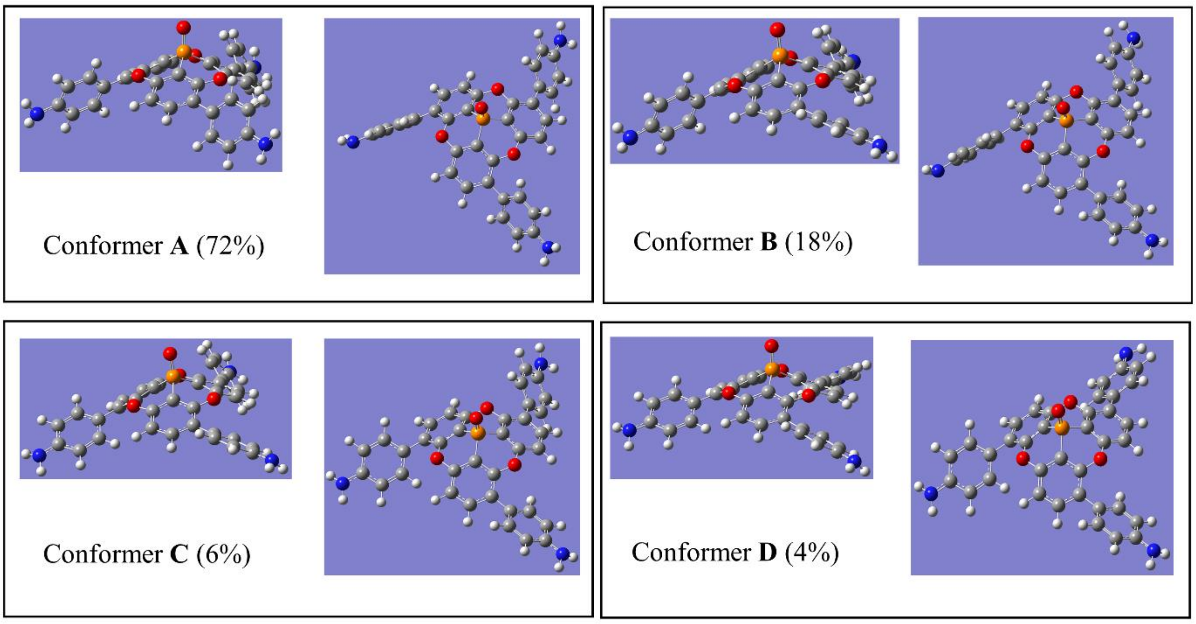

Supplementary Figure 87. Four conformer geometries for the (*M*)-enantiomer by DFT.

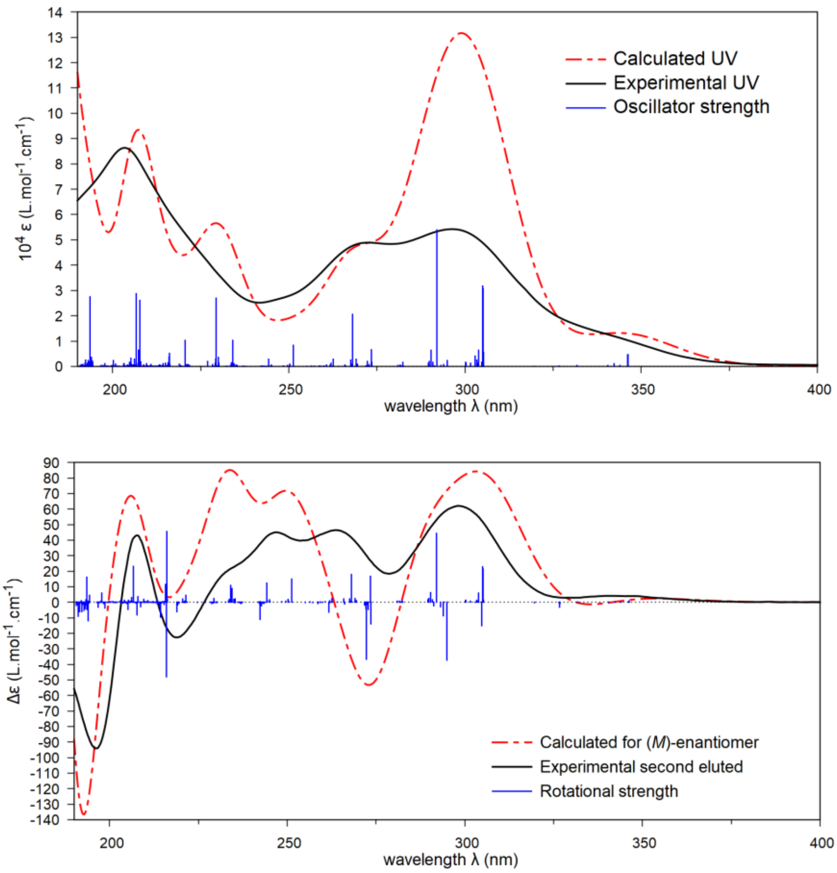

Supplementary Figure 88. Comparison of UV (top) and ECD (bottom) experimental spectra in acetonitrile for the second eluted enantiomer on Chiralpak IA and TD-DFT calculated spectra (σ = 0.20 eV, no shift). Vertical bars are oscillator and rotational strengths calculated for the conformers with arbitrary unit. Frequency calculations and TD-DFT calculations at SMD(CH_3CN)/PBE0/Def2TZVPP level: 150 excited stated were computed.

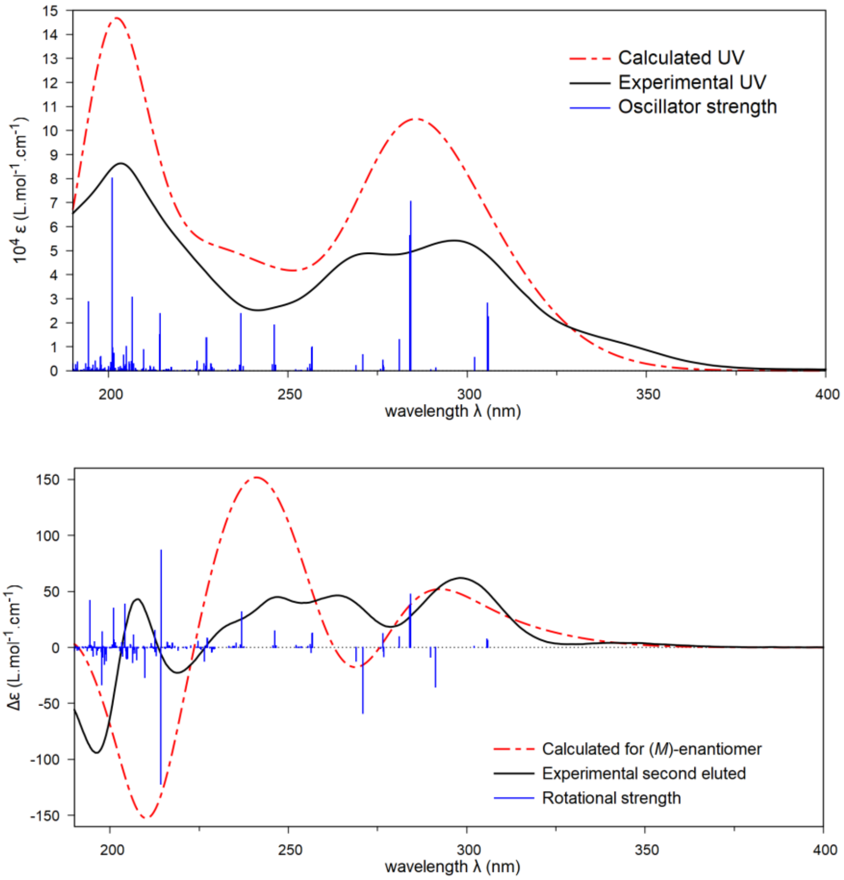

Supplementary Figure 89. Comparison of UV (top) and ECD (bottom) experimental spectra in acetonitrile for the second eluted enantiomer on Chiralpak IA and TD-DFT calculated spectra (σ = 0.35 eV, shifted by 10 nm). Vertical bars are oscillator and rotational strengths calculated for the conformers with arbitrary unit. Frequency calculations at SMD(CH_3CN) / B3LYP3 / 6-311G(3d,3p) level : conformer A (75%), conformer B (19%), conformer C (4%),and conformer D (2%). TD-DFT calculations at SMD(CH_3CN) / CAM-B3LYP / 6-31++G(3d,3p) level: 150 excited stated were computed.

9. X-ray Crystallography

Data were collected at Beamline I19 of Diamond Light Source employing silicon double crystal monochromated synchrotron radiation (0.6889 Å) with ω and ψ scans at 100(2) K.[10] Data integration and reduction were undertaken with Xia2.[11-13] Subsequent computations were carried out using the WinGX-32 graphical user interface[14] or Olex2.[15] Absorption corrections were applied to the data using the AIMLESS[16] tool in the CCP4 suite.[17] The structures were solved by intrinsic phasing using SHELXT[18] then refined and extended with SHELXL.[19] In general, non-hydrogen atoms with occupancies greater than 0.5 were refined anisotropically. Carbon-bound hydrogen atoms were included in idealised positions and refined using a riding model. Disorder was modelled using standard crystallographic methods including constraints, restraints and rigid bodies where necessary. Crystallographic data along with specific details pertaining to the refinement follow. Crystallographic data have been deposited with the CCDC (2068665-2068668).

[C_{70}⊂1]·8NTf$_2$ [+ solvent]

Formula $C_{302}H_{132}F_{48}N_{32}O_{48}P_4S_{16}Zn_4$, *M* 6786.71, Monoclinic, space group P 21/c (#14), *a* 20.9552(5), *b* 46.4983(9), *c* 30.1058(7) Å, β 92.350(2), *V* 29309.9(11) Å^3, D_c 1.538 g cm^{-3}, *Z* 4, crystal size 0.050 by 0.040 by 0.040 mm, colour pale brown, habit block, temperature 100(2) Kelvin, λ(Synchrotron) 0.6889 Å, μ(Synchrotron) 0.527 mm^{-1}, T(Analytical)$_{min,max}$ 0.989854413804384, 1.0, $2\theta_{max}$ 33.36, *hkl* range -17 17, -37 38, -24 23, *N* 58820, N_{ind} 17414(R_{merge} 0.1098), N_{obs} 5034(I > 2σ(I)), N_{var} 2659, residuals* $R1(F)$ 0.1201, $wR2(F^2)$ 0.3350, GoF(all) 1.018, $\Delta\rho_{min,max}$ -0.397, 0.596 e$^-$ Å^{-3}.

$^*R1 = \Sigma||F_o| - |F_c||/\Sigma|F_o|$ for $F_o > 2\sigma(F_o)$; $wR2 = (\Sigma w(F_o^2 - F_c^2)^2/\Sigma(wF_c^2)^2)^{1/2}$ all reflections

$w=1/[\sigma^2(F_o^2)+(0.1400P)^2]$ where $P=(F_o^2+2F_c^2)/3$

Specific refinement details:

The crystals of [C_{70}⊂**1**]·8NTf$_2$ were grown by layering diethyl ether with an acetonitrile solution of the complex in an NMR tube. The crystals employed immediately lost solvent after removal from the mother liquor and rapid handling prior to flash cooling in liquid nitrogen was required to collect data. Despite these measures and the use of synchrotron radiation few reflections at greater than 1.2 Å

resolution were observed and the data were trimmed accordingly. Furthermore there was a significant drop-off in diffraction intensity after around 1.5 Å resolution resulting in a low ratio of observed/unique reflections. Nevertheless, the quality of the data is far more than sufficient to establish the connectivity of the structure. The asymmetric unit was found to contain one complete $C_{70}{\subset}Zn_4L_4$ assembly and associated counterions and solvent molecules.

Due to the limited resolution of the data and high levels of thermal motion throughout the structure, substantial restraints were required in order to obtain a reasonable model for the organic parts of the structure. The GRADE program[20] was thus employed, using the GRADE Web Server,[21] to generate a full set of bond distance and angle restraints (DFIX, DANG, FLAT) for the organic ligands. Thermal parameter restraints (SIMU, RIGU) were applied to all atoms except for zinc and all light atoms were refined with approximate isotropic behaviour (ISOR).

The position of the C_{70} guest was established unequivocally from the electron density map. The C_{70} was found to be rotationally disordered around its longer axis and behaves as a shell of electron density, similar to disorder seen in other fullerene structures.[22-26] To facilitate realistic modelling of the C_{70} a rigid-body constraint based on a well-defined C_{70} from the CCDC (Refcode YOVWEN[27]) was used and the C_{70} was modelled as disordered over two locations with isotropic thermal parameters.

The anions within the structure also show evidence of substantial disorder and only a single triflimide lattice site could be resolved. Further reflecting the solvent loss and poor diffraction properties there is a substantial amount of void volume in the lattice containing smeared electron density from disordered solvent and the 7 unresolved anions per Zn_4L_4 tetrahedron (included as triflimide in the formula). These anions and were significantly disordered and despite numerous attempts at modelling, no satisfactory model for the electron-density associated with them could be found. Consequently the SQUEEZE[28] function of PLATON[29] was employed to remove the contribution of the electron density associated with these remaining anions and further highly disordered solvent, which gave a potential solvent-accessible void of 11008 Å^3 per unit cell (a total of approximately 4491 electrons). Diffuse solvent molecules could not be assigned to acetonitrile or diethyl ether and were therefore not included in the formula. Consequently, the molecular weight and density given above are underestimated.

CheckCIF gives 4 A and 3 B level alerts, mostly resulting from the limited resolution of the data (low ratio of observed /unique reflections, low data to parameter ratio, low bond precision) and isotropic modelling of the disordered C_{70}. Two alerts relating to short contacts of phenyl protons with those of the phosphangulene core appear to be intrinsic features of the structure although it should be noted that there is some uncertainty in the position of the phenyl protons due to thermal motion of these rings.

[$C_{60}\subset$1]·8BF$_4$ [+ solvent]

Formula $C_{276}H_{135}B_8F_{48}N_{24}O_{16}P_4Zn_4$, *M* 5427.04, Monoclinic, space group C2/c (#15), *a* 18.2343(8), *b* 44.765(3), *c* 36.3739(14) Å, β 97.201(4), *V* 29457(3) Å^3, D_c 1.154 g cm^{-3}, *Z* 4, crystal size 0.040 by 0.030 by 0.020 mm, colour light yellow, habit block, temperature 100(2) Kelvin, λ(Synchrotron) 0.6889 Å, μ(Synchrotron) 0.527 mm^{-1}, T(Analytical)$_{min,max}$ 0.9909599229538634, 1.0, $2\theta_{max}$ 40.30, *hkl* range -18 18, -44 44, -3636 *N* 44776, N_{ind} 15297 (R_{merge} 0.0816), N_{obs} 6727 (I > 2σ(I)), N_{var} 1584, residuals* $R1(F)$ 0.0880, $wR2(F^2)$ 0.2926, GoF(all) 0.955, $\Delta\rho_{min,max}$ -0.451, 0.703 e$^-$ Å^{-3}.

*$R1 = \Sigma||F_o| - |F_c||/\Sigma|F_o|$ for $F_o > 2\sigma(F_o)$; $wR2 = (\Sigma w(F_o^2 - F_c^2)^2/\Sigma(wF_c^2)^2)^{1/2}$ all reflections

$w=1/[\sigma^2(F_o^2)+(0.1400P)^2]$ where $P=(F_o^2+2F_c^2)/3$

Specific refinement details:

The crystals of **[$C_{60}\subset$1]·8BF$_4$ [+ solvent]** were grown by vapour diffusion of diethyl ether into an acetonitrile solution of the complex with one drop of saturated ^{n}Bu$_4$N(BF$_4$) acetonitrile solution. The crystals employed immediately lost solvent after removal from the mother liquor and rapid handling prior to flash cooling in liquid nitrogen was required to collect data. Despite these measures and the use of synchrotron radiation few reflections at greater than 1.00 Å resolution were observed and the data were trimmed accordingly. Nevertheless, the quality of the data is far more than sufficient to establish the connectivity of the structure. The asymmetric unit was found to contain half of a $C_{60}\subset Zn_4L_4$ assembly and associated BF_4^- counterions.

Due to the limited resolution of the data, thermal parameter restraints (SIMU, RIGU) were applied to some of the carbon atoms.

Two and a half tetrafluoroborate ions were identified and refined as the counterions. There is a substantial amount of void volume in the lattice containing smeared electron density from disordered solvent and 3 unresolved anions per Zn_4L_4 tetrahedron (included as tetrafluoroborate in the formula). These anions and were significantly disordered and despite numerous attempts at modelling, no satisfactory model for the electron-density associated with them could be found. Consequently the SQUEEZE[28] function of PLATON[29] was employed to remove the contribution of the electron density associated with these remaining anions and further highly disordered solvent, which gave a potential solvent accessible void of 10830 Å^3 per unit cell (a total of approximately 2981 electrons). Diffuse solvent molecules could not be assigned to acetonitrile or diethyl ether and were therefore not included in the formula. Consequently, the molecular weight and density given above are underestimated.

CheckCIF gives 1 A and 4 B level alerts, mostly resulting from the limited resolution of the data (low sine(theta_max)/wavelength, low bond precision) and the inconsistent formulas between the calculated ones and the reported one that includes the unresolved counterions.

[C_6H_{12}•corannulene⊂1]·8NTf_2·2MeCN·0.375Et_2O [+ solvent]

$C_{263.50}H_{163.75}F_{48}N_{34}O_{48.38}P_4S_{16}Zn_4$, *M* 6390.35, Monoclinic, space group P 21/n (#14), *a* 25.9511(6), *b* 46.2999(11), *c* 46.8263(10) Å, *β* 91.649(2), *V* 56240(2) Å^3, D_c 1.509 g cm^{-3}, *Z* 8, crystal size 0.040 by 0.030 by 0.030 mm, colour pale yellow, habit block, temperature 100(2) Kelvin, *λ*(Synchrotron) 0.6889 Å, *μ*(Synchrotron) 0.539 mm^{-1}, T(Analytical)$_{min,max}$ 0.9913169843380004, 1.0, 2θ_{max} 34.86, *hkl* range -22 22, -40 40, -39 40, *N* 113296, N_{ind} 38120(R_{merge} 0.1035), N_{obs} 11267(I > 2σ(I)), N_{var} 6138, residuals* $R1(F)$ 0.1274, $wR2(F^2)$ 0.3469, GoF(all) 1.061, $\Delta\rho_{min,max}$ -0.520, 0.690 e$^-$ Å^{-3}.

*$R1 = \Sigma||F_o| - |F_c||/\Sigma|F_o|$ for $F_o > 2\sigma(F_o)$; $wR2 = (\Sigma w(F_o^2 - F_c^2)^2/\Sigma(wF_c^2)^2)^{1/2}$ all reflections

$w=1/[\sigma^2(F_o^2)+(0.1400P)^2]$ where $P=(F_o^2+2F_c^2)/3$

Specific refinement details:

The crystals of [C_6H_{12}•corannulene⊂**1**]·8NTf_2·2MeCN·0.375Et_2O were grown by layering of diethyl ether with an acetonitrile solution of the complex in an NMR tube. The crystals employed immediately lost solvent after removal from the mother liquor and rapid handling prior to flash cooling in liquid nitrogen was required to collect data. Despite these measures and the use of synchrotron radiation few reflections at greater than 1.15 Å resolution were observed and the data were trimmed accordingly. Furthermore there was a significant drop-off in diffraction intensity after around 1.5 Å resolution resulting in a low ratio of observed/unique reflections. Nevertheless, the quality of the data is far more than sufficient to establish the connectivity of the structure. The asymmetric unit was found to contain two complete Zn_4L_4 assemblies and associated counterions and solvent molecules.

Due to the limited resolution of the data bond lengths and angles within pairs of chemically identical organic ligands were restrained to be similar to each other and thermal parameter restraints (SIMU, RIGU) were applied to all atoms except for zinc. Even with these restraints some thermal parameters

remain larger than ideal as a consequence of the high level of thermal motion throughout the structure. In particular the vertex incorporating Zn4 shows evidence of disorder which could not be resolved due to the limited resolution of the data. Some additional restraints (including DFIX, DANG, FLAT and ISOR) were applied to areas of the structure showing higher levels of thermal motion. Three of the ligand phenyl rings were modelled as disordered over two locations with bond length and angle restraints applied to achieve a reasonable model. These disordered phenyl rings were modelled isotropically.

The positions of the guest molecules were clearly established from the electron density map. However in all cases they showed a high degree of thermal motion and substantial restraints were required to achieve a reasonable refinement. Consequently it is not appropriate to analyse their conformation in detail. The GRADE program[20] was employed, using the GRADE Web Server,[21] to generate a full set of bond distance and angle restraints (DFIX, DANG, FLAT) for the corannulenes and DFIX and DANG restraints were also applied to the cyclohexanes. In all cases the guest molecules show larger than ideal thermal parameters, indicative of further unresolved disorder or thermal motion. No further discrete locations for the disordered guests could be resolved and there was no evidence of their disorder over the other three cage walls. Refining the occupancy of the guests didn’t result in significant improvement to the residuals hence they were refined with full occupancy.

The anions within the structure also show evidence of substantial disorder. Two triflimide anions were modelled as disordered over two locations and several others show evidence of further unresolved disorder. The occupancies of all located anions were freely refined, some were subsequently fixed at the obtained values. Substantial bond length and thermal parameter restraints were applied to facilitate a reasonable refinement of the disordered anions and solvent molecules and most low occupancy moieties were modelled with isotropic thermal parameters. The hydrogen atoms of some acetonitrile solvent molecules could not be located in the electron density map and were therefore not included in the model.

Further reflecting the solvent loss and poor diffraction properties there is a substantial amount of void volume in the lattice containing smeared electron density from disordered solvent and 4.5 unresolved anions per Zn_4L_4 tetrahedron (included as triflimide in the formula). These anions and were significantly disordered and despite numerous attempts at modelling, no satisfactory model for the electron-density associated with them could be found. Consequently the SQUEEZE[28] function of PLATON[29] was employed to remove the contribution of the electron density associated with these remaining anions and further highly disordered solvent, which gave a potential solvent-accessible void of 11552 Å^3 per unit cell (a total of approximately 3703 electrons). Diffuse solvent molecules could not be assigned to

acetonitrile or diethyl ether and were therefore not included in the formula. Consequently, the molecular weight and density given above are underestimated.

CheckCIF gives 2 A and 7 B level alerts, mostly resulting from the limited resolution of the data (low sine(theta_max)/wavelength, low data to parameter ratio, low bond precision), isotropic modelling of the disordered moieties, the presence of significant thermal motion within the structure and acetonitrile for which the hydrogen atoms were not modelled (apparent singly bonded carbons).

[1]·8NTf₂·1.375MeCN [+ solvent]

Formula $C_{234.75}H_{136.12}F_{48}N_{33.38}O_{48}P_4S_{16}Zn_4$, *M* 6002.46, Monoclinic, space group P 21/n (#14), *a* 26.1020(4), *b* 45.8594(7), *c* 45.5859(8) Å, *β* 92.319(2), *V* 54522.6(15) Å^3, D_c 1.462 g cm^{-3}, *Z* 8, crystal size 0.030 by 0.030 by 0.020 mm, colour pale yellow, habit block, temperature 100(2) Kelvin, *λ*(Synchrotron) 0.6889 Å, *μ*(Synchrotron) 0.550 mm^{-1}, T(Analytical)$_{min,max}$ 0.9435683736313124, 1.0, $2\theta_{max}$ 34.86, *hkl* range -22 22, -39 39, -39 39, *N* 117288, N_{ind} 37444(R_{merge} 0.0942), N_{obs} 17266(I > 2σ(I)), N_{var} 5376, residuals* $R1(F)$ 0.1404, $wR2(F^2)$ 0.4192, GoF(all) 1.139, $\Delta\rho_{min,max}$ -0.688, 1.293 e$^-$ Å^{-3}.

*$R1 = \Sigma||F_o| - |F_c||/\Sigma|F_o|$ for $F_o > 2\sigma(F_o)$; $wR2 = (\Sigma w(F_o^2 - F_c^2)^2/\Sigma(wF_c^2)^2)^{1/2}$ all reflections

$w=1/[\sigma^2(F_o^2)+(0.2000P)^2+200.0000P]$ where $P=(F_o^2+2F_c^2)/3$

Specific refinement details:

The crystals of [**1**]·8NTf₂·1.375MeCN were grown by vapour diffusion of diethyl ether into an acetonitrile solution of the complex. The crystals employed immediately lost solvent after removal from the mother liquor and rapid handling prior to flash cooling in liquid nitrogen was required to collect data. Despite these measures and the use of synchrotron radiation few reflections at greater than 1.15 Å resolution were observed and the data were trimmed accordingly. Nevertheless, the quality of the data is far more than sufficient to establish the connectivity of the structure. The asymmetric unit was found to contain two complete Zn_4L_4 assemblies and associated counterions and solvent molecules.

Due to the limited resolution of the data bond lengths and angles within pairs of chemically identical organic ligands were restrained to be similar to each other and thermal parameter restraints (SIMU, RIGU) were applied to all atoms except for zinc. Even with these restraints some thermal parameters remain larger than ideal as a consequence of the high level of thermal motion throughout the structure.

In particular the vertices incorporating Zn6 and Zn7 show evidence of disorder which could not be resolved due to the limited resolution of the data. Some additional restraints (including DFIX, DANG, FLAT and ISOR) were applied to areas of the structure showing higher levels of thermal motion. Two of the ligand phenyl rings were modelled as disordered over two locations with bond length and angle restraints applied to achieve a reasonable model. These disordered phenyl rings were modelled isotropically.

The anions within the structure also show evidence of substantial disorder. One triflimide anion was modelled as disordered over two locations and several others show evidence of further unresolved disorder. The occupancies of all located anions were freely refined. Substantial bond length and thermal parameter restraints were applied to facilitate a reasonable refinement of the disordered anions and solvent molecules and most low occupancy moieties were modelled with isotropic thermal parameters. The hydrogen atoms of some acetonitrile solvent molecules could not be located in the electron density map and were therefore not included in the model.

Further reflecting the solvent loss and poor diffraction properties there is a substantial amount of void volume in the lattice containing smeared electron density from disordered solvent and 6 unresolved anions per Zn_4L_4 tetrahedron (included as triflimide in the formula). These anions and were significantly disordered and despite numerous attempts at modelling, no satisfactory model for the electron-density associated with them could be found. Consequently the SQUEEZE[28] function of PLATON[29] was employed to remove the contribution of the electron density associated with these remaining anions and further highly disordered solvent, which gave a potential solvent accessible void of 18285 Å^3 per unit cell (a total of approximately 5798 electrons). Diffuse solvent molecules could not be assigned to acetonitrile or diethyl ether and were therefore not included in the formula. Consequently, the molecular weight and density given above are underestimated.

CheckCIF gives 2 A and 4 B level alerts, mostly resulting from the limited resolution of the data (low sine(theta_max)/wavelength, low data to parameter ratio, low bond precision) and isotropic modelling of the disordered phenyl rings and the presence of significant thermal motion within the structure.

Supplementary references:

1. Yamamura M, Saito T, Nabeshima T. Phosphorus-containing chiral molecule for fullerene recognition based on concave/convex interaction. *J Am Chem Soc*, **136,** 14299-14306 (2014).

2. Kishida N, Matsumoto K, Tanaka Y, Akita M, Sakurai H, Yoshizawa M. Anisotropic contraction of a polyaromatic capsule and its cavity-induced compression effect. *J Am Chem Soc*, **142,** 9599-9603 (2020).

3. García-Simón C, *et al.* Complete Dynamic Reconstruction of C60, C70, and (C59N)2 Encapsulation into an Adaptable Supramolecular Nanocapsule. *J Am Chem Soc*, **142,** 16051-16063 (2020).

4. Jensen FR, Noyce DS, Sederholm CH, Berlin AJ. The energy barrier for the chair-chair interconversion of cyclohexane. *J Am Chem Soc*, **82,** 1256-1257 (1960).

5. Nikitin K, O'Gara R. Mechanisms and Beyond: Elucidation of Fluxional Dynamics by Exchange NMR Spectroscopy. *Chem Eur J*, **25,** 4551-4589 (2019).

6. Kleywegt GJ, Jones TA. Detection, delineation, measurement and display of cavities in macromolecular structures. *Acta Cryst*, **D50,** 178-185 (1994).

7. Hristova YR, Smulders MMJ, Clegg JK, Breiner B, Nitschke JR. Selective anion binding by a "Chameleon" capsule with a dynamically reconfigurable exterior. *Chem Sci*, **2,** 638-641 (2011).

8. Gaussian 16 RA, M. J. Frisch, G. W. Trucks, H. B. Schlegel, G. E. Scuseria, M. A. Robb, J. R. Cheeseman, G. Scalmani, V. Barone, G. A. Petersson, H. Nakatsuji, X. Li, M. Caricato, A. V. Marenich, J. Bloino, B. G. Janesko, R. Gomperts, B. Mennucci, H. P. Hratchian, J. V. Ortiz, A. F. Izmaylov, J. L. Sonnenberg, D. Williams-Young, F. Ding, F. Lipparini, F. Egidi, J. Goings, B. Peng, A. Petrone, T. Henderson, D. Ranasinghe, V. G. Zakrzewski, J. Gao, N. Rega, G. Zheng, W. Liang, M. Hada, M. Ehara, K. Toyota, R. Fukuda, J. Hasegawa, M. Ishida, T. Nakajima, Y. Honda, O. Kitao, H. Nakai, T. Vreven, K. Throssell, J. A. Montgomery, Jr., J. E. Peralta, F. Ogliaro, M. J. Bearpark, J. J. Heyd, E. N. Brothers, K. N. Kudin, V. N. Staroverov, T. A. Keith, R. Kobayashi, J. Normand, K. Raghavachari, A. P. Rendell, J. C. Burant, S. S. Iyengar, J. Tomasi, M. Cossi, J. M. Millam, M. Klene, C. Adamo, R. Cammi, J. W. Ochterski, R. L. Martin, K. Morokuma, O. Farkas, J. B. Foresman, and D. J. Fox, Gaussian, Inc., Wallingford CT, 2016.

9. Specdis version 1.71. T. Bruhn AS, Y. Hemberger, G. Pescitelli. Berlin, Germany, 2017, https://specdis-software.jimdo.com/.

10. Allan D, *et al.* A Novel Dual Air-Bearing Fixed-χ Diffractometer for Small-Molecule Single-Crystal X-ray Diffraction on Beamline I19 at Diamond Light Source. *Crystals*, **7,** 336 (2017).

11. Evans P. Scaling and assessment of data quality. *Acta Cryst*, **D62,** 72-82 (2006).

12. Winter G. xia2: an expert system for macromolecular crystallography data reduction. *J Appl Crystallogr*, **43,** 186-190 (2010).

13. Winter G, *et al.* DIALS: implementation and evaluation of a new integration package. *Acta Cryst*, **D74,** 85-97 (2018).

14. Farrugia L. WinGX and ORTEP for Windows: an update. *J Appl Crystallogr*, **45,** 849-854 (2012).

15. Dolomanov OV, Bourhis LJ, Gildea RJ, Howard JAK, Puschmann H. OLEX2: a complete structure solution, refinement and analysis program. *J Appl Crystallogr*, **42,** 339-341 (2009).

16. Evans PR, Murshudov GN. How good are my data and what is the resolution? *Acta Cryst*, **D69,** 1204-1214 (2013).

17. Winn MD, *et al.* Overview of the CCP4 suite and current developments. *Acta Cryst*, **D67,** 235-242 (2011).

18. Sheldrick G. SHELXT - Integrated space-group and crystal-structure determination. *Acta Cryst*, **A71,** 3-8 (2015).

19. Sheldrick GM. Crystal structure refinement with SHELXL. *Acta Cryst*, **C71,** 3-8 (2015).

20. Bricogne G, *et al. BUSTER*, 2.11.2 edn. Global Phasing Ltd.: Cambridge, United Kingdom, 2011.

21. Smart OS, Womack TO. *Grade Web Server*. Global Phasing Ltd. , 2014.

22. Bi Y, Liao W, Wang X, Wang X, Zhang H. Mn4-hinged bithiacalix[4]arenes accommodating fullerenes. *Dalton Trans*, **40,** 1849-1851 (2011).

23. Rizzuto FJ, Wood DM, Ronson TK, Nitschke JR. Tuning the Redox Properties of Fullerene Clusters within a Metal–Organic Capsule. *J Am Chem Soc*, **139,** 11008-11011 (2017).

24. Toyota S, Yamamoto Y, Wakamatsu K, Tsurumaki E, Muñoz-Castro A. Nano-Saturn with an Ellipsoidal Body: Anthracene Macrocyclic Ring–C70 Complex. *Bull Chem Soc Jpn*, **92,** 1721-1728 (2019).

25. Nobukuni H, Kamimura T, Uno H, Shimazaki Y, Naruta Y, Tani F. Supramolecular Structures of Inclusion Complexes of C70 and Cyclic Porphyrin Dimers. *Bull Chem Soc Jpn*, **84,** 1321-1328 (2011).

26. Escudero-Adán EC, Bauzá A, Hernández-Eguía LP, Würthner F, Ballester P, Frontera A. Solid-state inclusion of C60 and C70 in a co-polymer induced by metal–ligand coordination of a Zn–porphyrin cage with a bis-pyridyl perylene derivative. *CrystEngComm*, **19,** 4911-4919 (2017).

27. Ghiassi KB, Bowles FL, Chen SY, Olmstead MM, Balch AL. Incorporation of the Similarly Sized Molecules, Diiodine and Carbon Disulfide, into Cocrystals Formed with the Fullerenes, C60 or C70. *Cryst Growth Des*, **14,** 5131-5136 (2014).

28. van der Sluis P, Spek AL. BYPASS: an effective method for the refinement of crystal structures containing disordered solvent regions. *Acta Cryst*, **A46,** 194-201 (1990).

29. Spek AL. *PLATON: A Multipurpose Crystallographic Tool*. Utrecht University: Utrecht, The Netherlands, 2008.
